# Single-Agent Sedation for Behavioral Management in Pediatric Dentistry: An Umbrella Review of Agents, Routes of Administration, Providers, and Clinical Settings

**DOI:** 10.3390/children13030373

**Published:** 2026-03-06

**Authors:** Federica Di Spirito, Francesco Giordano, Giuseppina De Benedetto, Maria Pia Di Palo, Francesco Traino, Colomba Pessolano, Alessia Bramanti, Antonino Fiorino, Carlo Rengo

**Affiliations:** 1 Department of Medicine, Surgery and Dentistry, University of Salerno, Via S. Allende, 84081 Baronissi, Italy; frgiordano@unisa.it (F.G.); giusydb15@gmail.com (G.D.B.); mdipalo@unisa.it (M.P.D.P.); cpessolano@unisa.it (C.P.); abramanti@unisa.it (A.B.); crengo@unisa.it (C.R.); 2 Department of Neuroscience, Reproductive Science and Dentistry, University of Naples Federico II, 80131 Naples, Italy; francesco.traino@unina.it

**Keywords:** sedation, conscious sedation, sedative agent, children, dental treatment, pediatric dentistry, midazolam, ketamine, nitrous oxide, dexmedetomidine

## Abstract

**Highlights:**

**What are the main findings?**

- The present umbrella review synthesized 18 systematic reviews (97 primary studies; 6877 children), identifying 14 single-agent sedation strategies (excluding deep sedation/general anesthesia) administered through eight different routes; sedation was consistently reported as an effective approach for behavioral management and completion of dental treatment in children.
- Agent selection and administration routes appeared to be influenced by the clinical setting (e.g., outpatient clinic or hospital) and provider type (e.g., dentist or anesthesiologist), while evidence concerning acceptability and satisfaction among children, caregivers, and providers remains notably limited.

**What are the implications of the main findings?**

- In appropriately monitored settings, selected single-agent sedation strategies may provide valuable means to support behavior management and dental treatment continuity in pediatric patients, when the chosen pharmacological approach is aligned with the provider’s expertise and available infrastructure.
- The influence of clinical setting and provider type on agent selection and sedation outcomes highlights the importance of focusing on patient-, caregiver-, and provider-related acceptability and satisfaction rates, to ensure high standards of quality in pediatric dental sedation.

**Abstract:**

**Background:** Dental fear and anxiety are highly prevalent in children, resulting in avoidance or incomplete dental treatment; sedation emerges as a possible behavioral management strategy. This umbrella review aimed to provide a structured and critical synthesis of the available knowledge on sedative single-agent efficacy and routes of administration employed for achieving sedation (excluding deep sedation/general anesthesia) during dental procedures in children for behavior management, as well as to evaluate acceptability and satisfaction for child, caregiver, and provider, and to assess the influence of clinical setting and provider. **Methods:** In line with the PRISMA statement, the protocol was registered on PROSPERO (CRD420251043738), and 18 systematic reviews were included and synthesized qualitatively. **Results**: Single-agent sedation was safe and effective for managing behavior in children during dental procedures, with midazolam and nitrous oxide being the most studied agents. Different routes of administration showed distinct characteristics in onset, recovery time, adverse effects and cooperation, while agent selection appeared influenced by clinical setting and provider type. However, data on acceptability and satisfaction from children, caregivers, and providers remains limited. **Conclusions:** Evidence suggests potential effectiveness of selected agents and routes in appropriately monitored settings, but data heterogeneity precludes strong comparative recommendations. Further studies are therefore needed to address the existing gaps in pediatric dental sedation.

## 1. Introduction

Dental fear is an unpleasant emotional state occurring in situations related to dental treatment, while dental anxiety is an unreasonable and excessive negative emotional reaction, and one of the main challenges for the successful management of children in dental settings [1,2]. In children, the prevalence of dental fear and anxiety (DFA) ranges between 10.0 and 29.3% [2] and was associated with patients with poor oral health who avoid dental visits, and a greater risk of not undergoing or completing dental treatment [1,3]. In fact, it was reported that at least 10% of children aged between 0 and 19 years had a level of DFA that hindered their ability to tolerate dental procedures [2]. Fear of painful anesthesia injections was identified as the most common reason for children’s refusal of dental treatment [1].

Several strategies have been proposed for the behavioral management of children with DFA, ranging from non-pharmacological techniques, such as cognitive behavioral therapy, tell–show–do, positive reinforcement, and audiovisual distraction, to pharmacological techniques such as sedation or general anesthesia [2,4].

Children with low or moderate levels of DFA can be effectively managed through non-pharmacological techniques, while in the management of phobic children or those with high DFA levels, pharmacological anesthetic agents should be required [5].

Several pharmacological anesthetic agents can be used through different routes of administration to achieve mild, moderate, or deep levels of sedation [6]. The most common pharmacological agents are midazolam, ketamine, propofol, sevoflurane, meperidine, fentanyl, hydroxyzine, and nitrous oxide–oxygen, which can be used alone or in combination and through the same or different route of administration [6].

In order to meet the need for DFA control, various degrees of sedation can be achieved, which, in accordance with the Ramsey scale, can be divided into anxiolysis, moderate sedation, deep sedation, and general anesthesia [7].

The most common indications for deeper sedation or general anesthesia comprise mental and psychological disorders, and complex or prolonged dental procedures, especially in children [8]. The management of more complex cases needing general anesthesia requires an equipped operating room and an anesthesiologist. While cardiovascular function is usually maintained, even in cases of deep sedation, patients may have a reduced level of consciousness but may potentially require assistance to maintain airway patency and adequate ventilation [5]. Since these infrastructure and staffing requirements cannot be available in all dental practices, or because in facilities where these conditions are met, dentists do not have sufficient operating slots, the number of patients waiting for general anesthesia is escalating [8].

Given the increasing clinical demand for sedation in pediatric dentistry and the limitations in resources and operating room availability, a broad and structured synthesis of the evidence is critically needed to guide clinical decision-making in dental settings. While the literature has extensively investigated the efficacy of pharmacological agents to achieve sedation in children [9,10,11], a comprehensive evidence synthesis is currently lacking. Although previous studies have investigated pharmacological sedation in pediatric dentistry, their scopes and analytical focuses vary substantially. Some studies compare different single agents against each other or against combination regimens; others focus exclusively on a single sedative agent; and several evaluate specific routes of administration across multiple agents. This heterogeneity in study design, inclusion criteria, and outcome reporting makes direct comparisons across studies difficult. As a consequence, clinicians may face uncertainty when selecting the most appropriate single-agent sedative and route of administration for routine practice. A higher-level synthesis that systematically integrates and standardizes the available evidence is therefore warranted. Specifically, there is an absence of umbrella reviews that extensively investigated the efficacy and safety of different standalone sedative agents (non-combination), which should be used in all dental settings, as well as the effectiveness of various routes of administration for the same agent or the impact of clinical setting and the provider’s expertise on the success of sedation. Finally, the outcomes related to the acceptability and satisfaction of the treatment from the perspective of the child, the caregiver, and the provider have not been highlighted.

Therefore, the present umbrella review aims primarily to provide a structured and critical synthesis of the available knowledge on sedative agents (single agent) and routes of administration employed for achieving sedation (excluding deep sedation and general anesthesia) during dental procedures in pediatric dentistry for behavior management; secondly, to evaluate for each sedative agent, route of administration, and sedation strategy the related acceptability and satisfaction among pediatric subjects and their caregivers, and providers; and furthermore, to assess the influence of the clinical setting (outpatient clinic, dental office, or operating room) and provider type on the selection, route of administration, sedation strategies, and sedation outcomes of sedative agents for achieving sedation during dental procedures.

## 2. Materials and Methods

### 2.1. Study Protocol

The study protocol was developed in accordance with the Preferred Reporting Items for Systematic Reviews and Meta-analyses (PRISMA) statement [12] and was registered on the International Prospective Register of Systematic Reviews (PROSPERO) under the identifier CRD420251043738. The protocol was established prior to the literature search, data extraction, and analysis.

The present umbrella review focused on the questions: “What is the efficacy of the different sedative agents and routes of administration, and which should be administered for achieving sedation (excluding deep sedation and general anesthesia) during dental procedures in pediatric dentistry for behavior management? How are different sedative agents, routes of administration, and sedation strategies accepted, and what are the rates of related satisfaction among pediatric subjects (≤18 years of age) and their caregivers, and usability for providers (dentists, physicians, anesthesiologists, sedationists, or trained non-anesthesiologists)? How do the clinical setting (outpatient clinic, dental office, or operating room) and provider type (dentist, physician, anesthesiologist, sedationist or trained non-anesthesiologist) influence the selection, route of administration, sedation strategies (single agent or combination), and sedation outcomes of sedative agents for achieving sedation (excluding deep sedation and general anesthesia) during dental procedures?”

The formulation of the research questions, along with the search strategy and criteria for study selection, was structured according to the PICO model [13], detailed as follows:

(P) Population: Pediatric subjects (≤18 years of age) undergoing dental procedures.

(I) Intervention: Any sedative agent (single-agent strategy) administered via any route for sedation (excluding deep sedation and general anesthesia) [14] by a dentist, physician, anesthesiologist, sedationist, or trained non-anesthesiologist in an outpatient setting, dental office, or operating room.

(C) Comparison: Different dosages and/or routes of administration of the same/different sedative agent; no sedation/placebo.

(O) Outcome(s):

- Primary outcomes:
  - Efficacy of the sedative agent administered via any route, measured through validated scales.
  - Efficacy of behavior management, measured through validated scales.
  - Complications or adverse events related to the sedative agent administration and adverse events occurring during and/or after treatment. Complications were defined as an unfavorable development or pathological event that occurs during the procedure, even when it has been performed correctly; adverse events were defined as an incident that causes harm to the patient, as defined by the World Health Organization in the “WHO Patient Safety Curriculum Guide for Medical Schools (available freely online on: https://iris.who.int/server/api/core/bitstreams/feadfc6c-7984-4d00-acb6-336a422bd8b9/content, accessed on 15 September 2025).
  - Completion of dental procedure (including treatment continuity, need for interruption, or early termination).
- Secondary outcomes:
  - Acceptability and perceived satisfaction of pediatric subjects, caregivers, and providers, measured through validated scales or self-reported data.
  - Influence of the clinical setting (outpatient, dental office, or operating room) on:
    (a) Selection of sedative agent, route of administration, sedation strategy;
    (b) Sedation outcomes.
  - Impact of provider type (dentist, physician, anesthesiologist, sedationist or trained non-anesthesiologist) on:
    (a) selection of sedative agent, route of administration, sedation strategy;
    (b) Sedation outcomes.

### 2.2. Search Strategy

Two independent reviewers (F.D.S and M.P.D.P.) conducted an electronic search to identify systematic reviews, encompassing both those with and without meta-analysis, up to 26 March 2025. No updated searches were performed after this date.

The search involved consulting the following databases and registers and applying filters according to availability:

- PubMed/MEDLINE: Article type “Systematic Review” and “Meta-analysis”;
- Scopus (search within: Title, abstract, keywords): Document type “Review”;
- Web of Science (search within: all fields): Document type “Review article”;
- PROSPERO register: Review status “Completed”;
- The Cochrane Library (search within: Title, abstract, keywords).

No date restrictions were applied.

The following keywords combined with Boolean operators were used (Appendix A, Table A1):

1. (“conscious sedation” OR “inhalatory conscious sedation” OR “intravenous conscious sedation” OR “enteral conscious sedation” OR “mild sedation” OR “nitrous oxide” OR “pharmacologic interventions” OR n2O OR benzodiazepine OR “Psychotropic Drugs” OR “intravenous sedation” OR “inhalatory sedation” OR “moderate sedation” OR “tranquilizing agents” OR sedation OR hypnosis OR ketamine OR midazolam)

AND

2. (dentistry OR pedodontics OR “dental treatment” OR “dental treatments” OR “dental procedures” OR “dental management”)

AND

3. (children OR child OR adolescent OR young OR pediatric OR youth)

### 2.3. Study Selection and Eligibility Criteria

After establishing the eligibility criteria, two independent reviewers (G.D.B. and F.G.) executed the study selection process. Any disagreements were addressed through discussion, with the involvement of a third reviewer (C.R.) to reach a consensus.

The titles and abstracts identified through the electronic search were screened to eliminate duplicates or records not relevant to the topic. For unclear titles and abstracts, the full text was obtained prior to exclusion. When access to the full text was not possible, the corresponding authors were contacted.

Additionally, a manual search was conducted by examining the reference list of the included studies to identify further eligible records.

All references from the selected studies were exported and organized using Mendeley Reference Manager version 2.120.3, copyright Elsevier Ltd. (Amsterdam, The Netherlands).

Inclusion criteria encompassed systematic reviews with or without meta-analysis, published in English, without any restrictions on date, sample size, or gender. Eligible studies involved human studies assessing the use of any sedative agent (single agent) administered via any route for sedation (excluding deep sedation and general anesthesia) by a dentist, physician, anesthesiologist, sedationist or trained non-anesthesiologist in an outpatient setting, dental office, or operating room in pediatric subjects (≤18 years of age) of any health status, comorbidity profile or behavior/cooperation level. Single-agent pharmacological sedation was defined as the administration of one sedative agent only, without concomitant use of additional sedatives or premedication.

Exclusion criteria encompassed non-systematic review studies, with or without meta-analysis, as well as previous versions of updated systematic reviews (only the most recent version was included if meeting the eligibility criteria); systematic reviews with or without meta-analysis conducted on adult subjects (>18 years of age), or in which it was not possible to extract data on pediatric subjects (≤18 years of age); systematic reviews with or without meta-analysis concerning deep sedation and general anesthesia or sedation administered prior to general anesthesia procedures, premedications, multidrug sedation strategies (e.g., midazolam plus nitrous oxide), or not related to dental procedures.

Sedation levels comprised minimal, conscious/moderate, and dissociative sedation, provided that airway patency, spontaneous ventilation, protective airway reflexes, and hemodynamic stability were maintained [14]. Deep sedation, although classified within the spectrum of sedation, according to the AAP [14], is characterized by a medically controlled depressed level of consciousness from which the patient is not easily arousable and may experience partial or complete loss of protective airway reflexes, including inability to maintain airway patency independently and diminished purposeful response to physical stimulation or verbal command. Due to these characteristics, which closely resemble general anesthesia, deep sedation is considered to lie beyond the scope of routine dental sedation. Given the inherent overlap and the labile boundary between deep sedation and general anesthesia, studies focusing on deep sedation were excluded to ensure a clear distinction and maintain the scope of the present umbrella review.

### 2.4. Data Extraction and Collection

The data were extracted by two independent reviewers (M.P.D.P.; A.B.) and were collected using a standardized data extraction form developed in alignment with established models proposed for RCT and non-RCT intervention reviews [15]. Any discrepancies were resolved through discussion and involving a third reviewer (A.F.).

The data extracted and collected from each included systematic review, whether or not a meta-analysis was performed, were as follows:

- Study: First author; year; journal; reference; number and design of included studies; meta-analysis (if any); quality assessment; funding.
- Population: Sample size; mean age/age range; gender ratio (M/F); comorbidities.
- Intervention: Sedative agent; route of administration; dosage; onset; latency; duration; sleep (if any); recovery time (time to recovery for readiness for discharge); provider (e.g., dentist, physician, anesthesiologist, sedationist or trained non-anesthesiologist); setting (e.g., outpatient, dental office, operating room); monitoring; dental procedure (e.g., restorative dental treatment, tooth extraction).
- Outcome(s):
  - Sedation efficacy (number of reported effective sedations; score, scales (e.g., Ramsay, Houpt): Sedation clinical efficacy was extracted as a dichotomous outcome (number of successful sedations on total number of sedations) as reported in the included studies. Moreover, sedation clinical efficacy outcomes were stratified separately for each sedation scale, reporting the score achieved.
  - Behavior score and scales.
  - Adverse events/complications (if any) and management of adverse events/complications (if any): Complications were defined as an unfavorable development or pathological event that occurs during the procedure, even when it has been performed correctly; adverse events were defined as an incident that causes harm to the patient, as defined by the World Health Organization in the “WHO Patient Safety Curriculum Guide for Medical Schools (available freely online on: https://iris.who.int/server/api/core/bitstreams/feadfc6c-7984-4d00-acb6-336a422bd8b9/content, accessed on 15 September 2025).
  - Procedure completion: Proportion of procedures completed without the need for interruption due to inadequate sedation, adverse events, or behavioral distress.
  - Reason for interruption (if any).
  - Acceptability (child, caregiver, provider).
  - Satisfaction (child, caregiver, provider).

Only data that adhered to the established eligibility criteria were included in the extraction process. Consequently, data not pertaining to pediatric populations, data unrelated to dental procedures, data concerning deep sedation or sedation prior to general anesthesia, or data related to multidrug sedation strategies were excluded from collection and extraction. Systematic reviews that included both single-agent and combination protocols were considered eligible; however, only the data specifically related to single-agent regimens were extracted, while data concerning multidrug strategies were excluded.

### 2.5. Data Synthesis

Data from the studies included in the present umbrella review were descriptively synthesized using Microsoft Excel Software 2019 (Microsoft Corporation, Redmond, WA, USA). A quantitative meta-analysis or formal comparative effect size pooling was not performed due to substantial methodological heterogeneity across the included reviews. Specifically, heterogeneity was observed in sedation scales, behavioral assessment tools, reporting-variable format (dichotomous vs. continuous), dosage regimens, routes of administration, and study designs of the primary studies included within each review. In addition, incomplete outcome reporting and missing data within several reviews further limited the feasibility of structured quantitative comparison.

Therefore, a structured narrative synthesis was conducted following a pre-specified analytical framework, and the synthesis was organized hierarchically as follows:

(i) First, by a sedative agent;
(ii) Then, by route of administration for each agent;
(iii) Finally, by outcome domains.

The data synthesis process was structured based on the following goals:

- To evaluate sedative agents administered via any route for sedation (excluding deep sedation and general anesthesia) during dental procedures in pediatric subjects (≤18 years of age) executed by a dentist, physician, anesthesiologist, seditionist, or trained non-anesthesiologist in an outpatient setting, dental office, or operating room;
- To assess the dosages and/or routes of administration of the same/different sedative agents in sedation (excluding deep sedation and general anesthesia) during dental procedures in pediatric subjects (≤18 years of age);
- To evaluate the efficacy of sedation (excluding deep sedation and general anesthesia) in behavior management;
- To summarize reported efficacy outcomes of sedation (excluding deep sedation and general anesthesia) compared to no sedation/placebo in behavior management;
- To evaluate different dosages and/or routes of administration of the same/different sedative agents in behavior management;
- To evaluate complications or adverse events related to the sedative-agent administration and adverse events occurring during and/or after treatment, the completion of dental procedures, and the reason for interruption (if any);
- To evaluate acceptability and satisfaction from pediatric subjects (≤18 years of age) and their caregivers;
- To evaluate the acceptability and satisfaction of providers (dentist, physician, anesthesiologist, sedationist or trained non-anesthesiologist);
- To assess the effect of the setting (outpatient setting, dental office, or operating room) and the provider (dentist, physician, anesthesiologist, sedationist or trained non-anesthesiologist) performing sedation (excluding deep sedation and general anesthesia) during dental procedures in pediatric subjects (≤18 years of age);
- To evaluate the impact of the clinical setting (outpatient, dental office, or operating room) on the selection of sedative agent, route of administration, sedation strategy, and sedation outcomes;
- To assess the impact of provider type (dentist, physician, anesthesiologist, sedationist or trained non-anesthesiologist) on the selection of sedative agent, route of administration, sedation strategy, and sedation outcomes;
- To evaluate the sedative agent, route of administration, sedation strategy, and sedation outcomes in relation to the type of dental procedure performed.

### 2.6. Quality Assessment and Overlap Management

Two independent authors (M.P.D.P. and A.F.) assessed the included studies in the present umbrella review qualitatively through the Assessing the Methodological Quality of Systematic Reviews 2 (AMSTAR) tool, available online at https://amstar.ca/ (accessed on 3 December 2025), which evaluates systematic reviews with or without meta-analysis for methodological confidence. The quality assessment was conducted after the final selection of eligible reviews, in accordance with the protocol registered in PROSPERO. No additional study selection based on the study quality or search updates was performed after the original database search conducted on 26 March 2025. In cases of disagreement in the evaluation, a third author (C.R.) was consulted for further discussion.

To calculate the primary studies’ overlap among the included systematic reviews, the corrected cover area (CCA) was assessed.

## 3. Results

### 3.1. Study Selection

The electronic search returned 463 results: specifically, 95 in PubMed/MEDLINE, 181 in Scopus, 158 in Web of Science, 22 in PROSPERO register, and 7 in the Cochrane Library. A total of 112 duplicate records were removed before screening. Of the remaining 351 title/abstracts, 237 records were not relevant to the study topic and were excluded. Five records were not retrieved; contact was made with the study authors, but no response was obtained, and the records were excluded.

The remaining 109 records were assessed for eligibility, of which a total of 91 records were excluded for the following reasons: 41 were not systematic review, 10 did not involve sedation, 10 were previous versions of updated reviews, 8 did not involve dental procedures, 6 involved deep sedation/general anesthesia, 4 due to inability to extract data on the pediatric population, 4 were not in English, 3 were on adults, 3 due to inability to extract data on single-agent/combination strategies agents.

A total of 18 systematic reviews [16,17,18,19,20,21,22,23,24,25,26,27,28,29,30,31,32,33] met the eligibility criteria and were included before the manual search.

From the additional manual search from the references of the included studies, a total of 762 records were obtained, from which 130 duplicates were removed. Of the 632 remaining records, 596 were not relevant to the study topic and were excluded. Four records were not retrieved and were excluded. The remaining 32 records were assessed for eligibility, and full-text screening led to the exclusion of all 32 records for the following reasons: 21 were not systematic reviews, 4 did not involve dental procedures, 2 were on adult populations, 2 were on premedications, 2 due to inability to extract data on single-agent/combination strategies, 1 due to inability to extract data on the pediatric population.

Finally, a total of 18 systematic reviews [16,17,18,19,20,21,22,23,24,25,26,27,28,29,30,31,32,33] were included in the present umbrella review.

**Figure 1:**
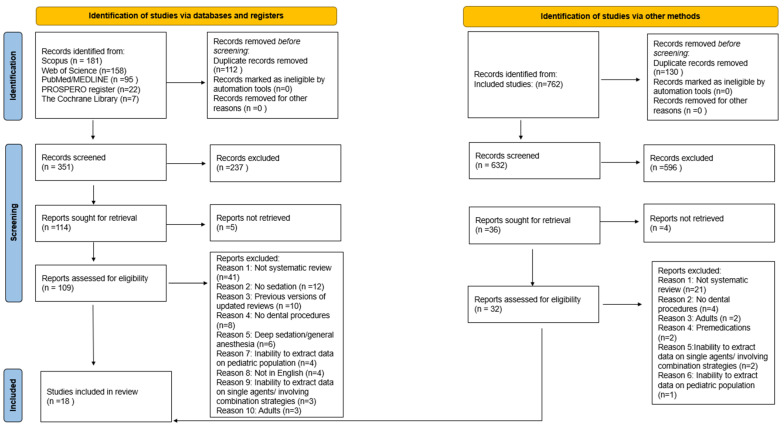
PRISMA flowchart for the electronic and manual search.

Figure 1 displays the PRISMA flowchart for the electronic and manual search.

### 3.2. Study Characteristics and Qualitative Synthesis

The data extracted from the 18 systematic reviews [16,17,18,19,20,21,22,23,24,25,26,27,28,29,30,31,32,33] included in the present umbrella review are shown in Supplementary File S1, which details study characteristics, populations, interventions, and primary and secondary outcomes. Supplementary File S2 presents the data extracted from the included systematic reviews, clustered by sedative agent and route of administration.

Across the included systematic reviews [16,17,18,19,20,21,22,23,24,25,26,27,28,29,30,31,32,33], a total of 97 studies were included: 88 randomized controlled trials and 9 observational studies. Among the included systematic reviews, 5 were with meta-analysis [16,17,19,25,29], whereas the remaining 13 were without meta-analysis [18,20,21,22,23,24,26,27,28,30,31,32,33].

**Figure 2:**
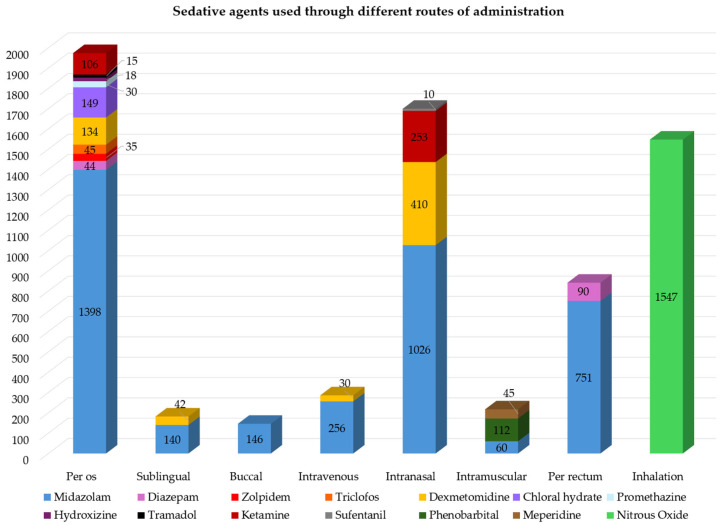
Sedative single agents used through different routes of administration.

In total, 14 different types of single sedative agents were used through eight different types of route of administration, involving 6877 children (Figure 2).

The overall mean age was 6.00 years, reported in 1852 pediatric subjects, with an age range from 0.1 to 17 years in 5031. The gender ratio was registered for 1111 children, comprising 554 males and 557 females (1.00 M/1.01 F). The mean weight reported was 14.34 kg in 657 children, ranging from 9 to 27 kg (*n* = 57).

The absence of comorbidities was declared in 1188 children, while in 529 pediatric subjects, the following comorbidities were reported: intellectual disability (*n* = 503) and autism (*n* = 26).

The providers who performed sedation were specified for 1765 procedures as follows: dentists for 824 procedures, anesthesiologists for 679, anesthesiologists and dentists for 150; specialists trained in pediatric sedation and life support for 42 pediatric subjects, dental nurses for 20, nurses for 20, dental assistants for 16, and pediatric dentists for 14 children.

The clinical setting in which the sedation was performed was specified for 2056 procedures as follows: an outpatient dental clinic for 1389 pediatric subjects, a university hospital for 478, a dental clinic/office for 102, a dental clinic and operating room transfer if necessary for 57, a pedodontic clinic for 16, and an operating room for 14 pediatric subjects.

Concerning the monitoring of vital signs, the following parameters were declared as being monitored: oxygen saturation in 1971 children, heart rate in 1286, respiratory rate in 999, blood pressure in 900, electrocardiogram in 194, visual assessment in 170, pre-tracheal auscultation in 170, and capnography in 10. It was declared that the monitoring of undefined vital signs was performed for an additional 547 children.

The types of dental procedures performed were specified for 3674 procedures as follows: tooth extraction (*n* = 1048), restorative dental therapy (*n* = 846), oral examination (*n* = 724), non-specified oral surgery (*n* = 584), and oral hygiene (*n* = 472).

The number of reported successful sedations was registered in 1857 pediatric subjects.

No adverse events/complications were registered in 1204 pediatric subject, while in 666, adverse events/complications were as follows: undefined events/complications (*n* = 153) [16,20,30,31]; headache (*n* = 89); oxygen desaturation/hypoxemia (*n* = 57); sleepiness/faint (*n* = 54); nausea (*n* = 52); disinhibitory reactions (*n* = 46); vomiting (*n* = 45); eating/swallowing difficulties (*n* = 30); hallucination (*n* = 26); vertigo (*n* = 22); sore mouth (*n* = 16); cough (*n* = 12); hiccups (*n* = 15); speaking impairment (*n* = 10); sneezing (*n* = 8); confusion (*n* = 7); euphoria (*n* = 5); paradoxical reactions (*n* = 5); diplopia (*n* = 3); emergency reaction (*n* = 2); salivation (*n* = 2); otalgia (*n* = 2); sweating (*n* = 2); crying (*n* = 1); epistaxis (*n* = 1); bradycardia (*n* = 1); unusually quiet at 24 h post intervention (*n* = N/d); and amnesia (*n* = N/d).

Management of adverse events was reported for six occasions as follows: none, spontaneous recovery (*n* = 4), oxygen application (*n* = 2).

Dental procedure completion was reported in 1929 cases, in which 1551 children completed the procedures, with difficulties in 89 other procedures, while no completion was reported in 289 children. The reason for procedure interruption was specified for 53 interruptions as follows: lack of cooperation (*n* = 45); inability to tolerate agent (*n* = 5); dental procedure refusal (*n* = 2); and paradoxical reactions (*n* = 1).

#### 3.2.1. Midazolam

In a total of 15 studies [16,18,19,20,21,22,24,25,26,27,28,30,31,32,33], midazolam was used as a single sedative agent, involving 3777 children.

The reported mean age was 4.72 years in 415 pediatric subjects [16,24,31,33], with an age range from 1.3 to 16 years in 2836 [16,18,20,21,22,26,28,30,32].

The reported gender ratio was 164 males to 150 females (1.09 M/1 F) [16,31].

The mean weight reported was 17.62 kg (kg) in 240 children [16], ranging from 9 to 27 kg (*n* = 57) [25].

The absence of comorbidities was declared in 530 children [16,19,24,30], while in 44 pediatric subjects, the following comorbidities were reported: intellectual disability (*n* = 31) [31] and autism (*n* = 13) [33].

The mean dosage reported was 0.48 mg/kg (*n* = 3393) [16,18,19,20,21,22,24,25,26,27,28,30,31,32]. The mean onset was 14.21 min (min) [16,20,21,25,32], with a range of 1.6 to 35 min (*n* = 241) [26,28]. The mean duration reported was 140.77 min (*n* = 35) [21,24], with a range of 45 to 79 min (*n* = 136) [21]. The absence of sleep was reported in 25 pediatric subjects [16]. The mean recovery time reported was 29.94 min (*n* = 330) [16,20,25], with a time range of less than 10 min (*n* = 10) [16].

The provider types performing sedation with midazolam were as follows: anesthesiologist for 331 pediatric subjects [16,19,30]; dentist for 85 [16]; anesthesiologist and dentist for 77 [16,25,31]; dental nurse for 20 [16]; dental assistant for 16 [16]; and specialist trained in pediatric sedation and life support for 42 pediatric subjects [20].

The clinical setting was reported as follows: outpatient dental clinic for 1314 pediatric subjects [19,21,30], dental clinic/office for 31 [18,24]; dental clinic and operating room transfer if necessary for 21 [25]; pedodontic clinic for 16 [16]; and university hospital for 10 pediatric subjects [16].

Monitoring was recorded as follows: oxygen saturation in 1157 children [16,20,21,25], respiratory rate in 410 [16,20,25], heart rate in 563 [16,20,25], electrocardiogram (ECG) in 194 [16], blood pressure in 453 [16,20], and undefined vital signs in 30 pediatric subjects [19].

The types of dental procedures performed were as follows: restorative dental therapy (*n* = 242) [16,19,20,24] and tooth extraction (*n* = 227) [16,20].

The number of reported successful sedations was registered in 507 pediatric subjects [21,22,24,26,27,28,31,32].

Mean sedation score was reported as follows: “moderate” for the Modified Ramsay sedation scale in 35 children [20]; 3.3 for the Breitkopf and Buttner in 90 [20]; 4 for Wilton’s sedation scale in 1 child [16]; “adequate” for a five-point scale in 38 [26]; “satisfactory” for a five-point scale in 15 [26]; range score of 4/5 for a five-point scale in 21 children [16]; 4.27 for an eight-point scale in 15 [16]; 4 for a 10-point scale in 20 [16,26]; and 3.3 for an undefined degree of sedation scale in 30 children [16].

Mean behavior score was reported as follows: 4.68 for the Houpt scale in 230 children [16,20,28]; range score between 5/6 in 8 children [24], less than score 5/6 in 3 [24], and between 3/4 in 20 for the Houpt scale [28]; 5.29 for the Modified Houpt scale in 38 [28]; 19.04 for the Children’s Fear Survey Schedule-Dental Subscale (CFSS-DS) in 82 [20,32]; 3.83 for the Face, Legs, Activity, Cry, Consolability (FLACC) scale in 56 [20]; 2.09 for the Ohio State University Behavioral Rating Scale (OSUBRS) in 44 [16]; 39.4 for the Spielberg anxiety inventory in 36 [20]; 0.45 for Vehnam’s clinical anxiety scale in 20 [16]; 3 for the Modified Frankl scale in 13 [31]; “excellent” in 86, “adequate” in 18, and “satisfactory” in 33 for the Global behavior rating scale; 2.9 for a three-point scale in 16 [16]; and 2.78 for the movement, crying, overall sedation and behavior scale in 93 children [16].

No adverse events/complications were registered in 538 pediatric subjects [16,19,24,25,27,30,33], while in 484, adverse events/complications were as follows: undefined events/complications (*n* = 153) [16,20,30,31]; oxygen desaturation/hypoxemia (*n* = 54) [16,21]; disinhibitory reactions (*n* = 46) [16]; headache (*n* = 36) [16,20]; sleepiness/faint (*n* = 34) [16,20]; nausea (*n* = 24) [16,20]; vomiting (*n* = 24) [16,25]; hallucination (*n* = 21) [16]; sore mouth (*n* = 14) [16]; vertigo (*n* = 13) [16,20]; cough (*n* = 12) [16]; hiccups (*n* = 11) [16]; speaking impairment (*n* = 10) [16]; sneezing (*n* = 8) [16]; confusion (*n* = 7) [16]; euphoria (*n* = 5) [16]; paradoxical reactions (*n* = 4) [20,21]; diplopia (*n* = 3) [16]; salivation (*n* = 2) [16]; sweating (*n* = 2) [16]; bradycardia (*n* = 1) [16]; unusually quiet at 24 h post intervention (*n* = N/d) [16]; and amnesia (*n* = N/d) [21].

Management of adverse events was reported as follows: oxygen application (*n* = 2) [21]; and none, with spontaneous recovery (*n* = 1) [16].

Dental procedure completion was reported in 854 children [16,20,21,28,30] and completion with difficulties in 89 [21], while the procedure was not completed in 117 children [16,20,28,30]. The reason for procedure interruption was reported as follows: inability to tolerate agent (*n* = 5) and paradoxical reactions (*n* = 1).

Acceptability was reported as follows: “excellent” (*n* = 18), “good” (*n* = 6), “moderate” (*n* = 1), and “poor” (*n* = 1), according to a four-point scale [16]; “acceptance” (*n* = 28), according to the Al-Rakaf scale [16]; “no complaints in acceptance” (*n* = 25) [32] and “well-accepted” (*n* = 21) [27] according to self-report from the child. No acceptability data were reported for caregivers and providers.

Satisfaction was reported as follows: 4.69 ± 0.7 (*n* = 194), according to a 10-point scale in caregivers [16]; 2.7 (*n* = 30), according to a 3-point scale [19]; and “very effective” (*n* = 31) [32] and “effective” (*n* = 31) according to self-report in providers [32]. No satisfaction data were reported in child.

A total of seven different routes of administration of midazolam were recorded: per os in 11 studies [16,18,20,21,24,25,28,30,31,32,33]; sublingual in 4 [16,22,28,32]; buccal in 3 [20,28,32]; intravenous in 4 [16,18,19,20]; intranasal in 9 [16,20,22,25,26,27,28,30,32]; intramuscular in 3 [16,28,32]; and per rectum in 2 [16,21].

Table 1 shows the population and intervention characteristics, as well as the primary and secondary outcomes, sorted by administration routes of midazolam.

#### 3.2.2. Diazepam

In a total of 5 studies [16,17,18,21,33], diazepam was used as a single sedative agent, involving 134 children.

The reported mean age was 4.02 years in 58 pediatric subjects [16,33], with an age range from 1.5 to 14.7 years in 134 [16,17,18,21,33].

The reported gender ratio was 23 males to 22 females (1.05 M/1 F) [16].

The mean weight was never reported.

The following comorbidities were reported: autism (*n* = 13).

The mean dosage reported was 0.63 mg/kg (*n* = 123) [16,18,21,33]. The onset, duration, sleep status and recovery time were never registered.

The provider types performing sedation with diazepam were as follows: dentist for 45 [16] and anesthesiologist for 10 pediatric subjects [18].

The clinical setting was reported as follows: outpatient dental clinic for 45 pediatric subjects [21] and dental clinic/office for 10 [18].

Monitoring was recorded as follows: oxygen saturation in 10 children, respiratory rate in 10, and blood pressure in 10 pediatric subjects [16].

The types of dental procedures performed were registered as follows: dental examination in 11 children [17].

The number of reported successful sedations was registered as 38 pediatric subjects [16,33]. Mean sedation score was reported as follows: “agitated” for an undefined degree-of-sedation scale in 13 children [16].

Mean behavior score was reported as follows: 4.5 for the Houpt scale in 10 children [16].

No data on adverse events/complications, management of adverse events, dental procedure completion, reason for interruption, acceptability, or satisfaction were reported in the included studies.

A total of two different routes of administration of diazepam were recorded: per os in four studies [16,17,18,33] and per rectum in two [16,21].

Table 2 shows the population and intervention characteristics, as well as the primary outcomes, sorted by administration routes of diazepam. Categories with missing data for all administration routes are not displayed in the table.

#### 3.2.3. Zolpidem

In one study [2], zolpidem was used as a single sedative agent, involving 35 children using one route of administration: per os.

Table 3 shows the population and intervention characteristics, as well as the primary outcomes, sorted by administration route of zolpidem. Categories with missing data for all administration routes are not displayed in the table.

#### 3.2.4. Triclofos

In one study [16], triclofos was used as a single sedative agent, involving 45 children using one route of administration: per os.

Table 4 shows the population and intervention characteristics, as well as the primary outcomes, sorted by administration route of triclofos. Categories with missing data for all administration routes are not displayed in the table.

#### 3.2.5. Dexmedetomidine

In a total of seven studies [16,19,25,26,27,29,32], dexmedetomidine was used as a single sedative agent, involving 616 children.

The reported mean age was 7.00 years in 126 pediatric subjects [16], with an age range from 3 to 14 years in 405 [16,22,25].

The reported gender ratio was 38 males to 46 females (1 M/1.21 F) [16].

The mean weight reported was 14.82 kg in 126 children [16].

The absence of comorbidities was declared in 225 children [16,25].

The mean dosage reported was 1.68 mg/kg (*n* = 608) [16,19,22,25,27,32]. The mean onset was 17.51 min (*n* = 85) [25,29], with a range of 7 to 25 min (*n* = 22) [27]. The duration was not registered. Sleep was reported in 14 pediatric subjects [29]. The mean recovery time reported was 24.5 min (*n* = 36) [25].

The provider types performing sedation with dexmedetomidine were as follows: anesthesiologist for 84 pediatric subjects [16,29]; anesthesiologist and dentist for 42 [25]; and pediatric dentist for 14 pediatric subjects [29].

The clinical setting was reported as follows: dental clinic and operating room transfer if necessary for 36 pediatric subjects [25]; outpatient dental clinic for 30 [19,29]; and operating room for 14 [29].

Monitoring was recorded as follows: oxygen saturation in 222 children [16,29], blood pressure in 222 [16,29], respiratory rate in 154 [16,29], heart rate in 96 [16,29], and undefined vital signs in 30 pediatric subjects [19].

The types of dental procedures performed were registered as follows: undefined dental surgery (*n* = 78) [25,29], tooth extraction (*n* = 55) [19,25], and restorative therapy (*n* = 17) [19].

The number of reported successful sedations was registered as 156 pediatric subjects [22]. Mean sedation score was reported as follows: “satisfactory” on a five-point scale in 39 children [27]; “satisfactory” for the modified American Academy of Pediatric Dentistry (AAPD) scale in 23 [16]; and 3.61 for the Modified Observer Assessment of Alertness/Sedation scale (MOAAS) in 14 children [29].

The mean behavior score was reported as follows: 3.75 for the FLACC scale in 42 children [27] and “acceptable” for an undefined behavior scale in 22 children [32].

No adverse events/complications were registered in 203 pediatric subjects [16,19,25], while in 1, adverse events/complications were as follows: vomiting (*n* = 1) [25].

No data about management of adverse events, dental procedure completion or reason for procedure interruption was registered.

Acceptability was reported as follows: “well-accepted” (*n* = 42) and “far to excellent acceptance”, according to a self-reported scale (*n* = 16) from the child [27]. No acceptability data were reported for caregivers or providers.

No satisfaction data were reported.

A total of four different routes of administration of dexmedetomidine were recorded: intranasal in six [16,25,26,27,29,32]; per os in three studies [16,27,29], sublingual in one [32]; and intravenous in 1 [19].

Table 5 shows the population and intervention characteristics, as well as the primary and secondary outcomes, sorted by administration routes of dexmedetomidine. Categories with missing data for all administration routes are not displayed in the table.

#### 3.2.6. Chloral Hydrate

In two studies [16,17], chloral hydrate was used as a single sedative agent, involving 149 children using one route of administration: per os.

Table 6 shows the population and intervention characteristics, as well as the primary outcomes, sorted by administration route of chloral hydrate. Categories with missing data for all administration routes were not displayed in the table.

#### 3.2.7. Promethazine

In one study [16], promethazine was used as a single sedative agent, involving 30 children using one route of administration: per os.

Table 7 shows the population and intervention characteristics, as well as the primary outcomes, sorted by administration route of promethazine. Categories with missing data for all administration routes are not displayed in the table.

#### 3.2.8. Hydroxyzine

In one study [16] hydroxyzine was used as a single sedative agent, involving 18 children using one route of administration: per os.

Table 8 shows the population and intervention characteristics, as well as the primary outcomes, sorted by administration route of hydroxyzine. Categories with missing data for all administration routes are not displayed in the table.

#### 3.2.9. Phenobarbital

In one study [17], phenobarbital was used as a single sedative agent, involving 112 children using one route of administration: intramuscular.

Table 9 shows the population and intervention characteristics, as well as the primary outcomes, sorted by administration route of phenobarbital. Categories with missing data for all administration routes are not displayed in the table.

#### 3.2.10. Tramadol

In one study [16], tramadol was used as a single sedative agent, involving 15 children using one route of administration: per os.

Table 10 shows the population and intervention characteristics, as well as the primary outcomes, sorted by administration route of tramadol. Categories with missing data for all administration routes are not displayed in the table.

#### 3.2.11. Meperidine

In one study [16], meperidine was used as a single sedative agent, involving 45 children using one route of administration: intramuscular.

Table 11 shows the population and intervention characteristics, as well as the primary outcomes, sorted by administration route of meperidine. Categories with missing data for all administration routes are not displayed in the table.

#### 3.2.12. Sufentanil

In one study [16], sufentanil was used as a single sedative agent, involving 10 children using one route of administration: intranasal.

Table 12 shows the population and intervention characteristics, as well as the primary outcomes, sorted by administration route of sufentanil. Categories with missing data for all administration routes are not displayed in the table.

#### 3.2.13. Ketamine

In a total of seven studies [16,22,25,26,27,29,32], ketamine was used as a single sedative agent, involving 359 children.

The reported mean age was 5.15 years in 192 pediatric subjects [16], with an age range from 1.42 to 14 years in 273 [16,22,25,26,27,32].

The reported gender ratio was 44 males to 42 females (1.05 M/1 F) [16,29].

The mean weight reported was 18.38 kg in 49 children [16].

The absence of comorbidities was declared in 79 children [16].

The mean dosage reported was 6.29 mg/kg (*n* = 272) [16,26,27,29,32]. The mean onset was 14.62 min [25,29,32], with a range of 3.6 to 11.6 min (*n* = 66) [26]. The duration reported and the sleep status were never assessed in the included studies. The mean recovery time reported was of 44.19 min (*n* = 21) [25], with a time range between 10 and 30 min in three children, and less than 10 min in seven [16].

The provider types performing sedation with ketamine were as follows: anesthesiologist and dentist for 31 pediatric subjects [16,25]; and anesthesiologist for 21 [16].

The clinical setting was a university hospital for 10 pediatric subjects [16].

Monitoring was recorded as follows: oxygen saturation in 90 children [16,25,26,29], respiratory rate in 70 [16,25,29], blood pressure in 70 [16,25,29], heart rate in 42 [16,25], and capnography in 10 pediatric subjects [16].

The types of dental procedures performed were registered as follows: tooth extraction (*n* = 61) [16,25] and undefined oral surgery (*n* = 28) [29].

The number of reported successful sedations was registered as 155 pediatric subjects.

The mean sedation score was reported as follows: 4 for a 10-point scale in 20 children [16,26]; and “satisfactory” (*n* = 16) ora 4–5 score (*n* = 14) for a five-point scale [26].

The mean behavior score was reported as follows: 3.5 on the FLACC scale in 21 [27], and “good/better behavior” for the movement, crying, overall sedation and behavior scale in 28 children [16].

No adverse events/complications were registered in 41 pediatric subject [25,27], while in 17, adverse events/complications were as follows: vomiting (*n* = 6) [16,25]; hallucination (*n* = 5) [16]; oxygen desaturation (*n* = 3) [16,26]; undefined emergency reaction (*n* = 2) [16]; and paradoxical reactions (*n* = 1) [16].

Management of adverse events was reported as follows: none and spontaneous recovery (*n* = 3) [16,26].

Dental procedure completion and reason for procedure interruption were never reported.

Acceptability and satisfaction rates were never reported in the included studies.

A total of two different routes of administration of ketamine were recorded: per os in two studies [16,29] and intranasal in seven [16,22,25,26,27,29,32].

Table 13 shows the population and intervention characteristics, as well as the primary and secondary outcomes, sorted by administration routes of ketamine. Categories with missing data for all administration routes are not displayed in the table.

#### 3.2.14. Nitrous Oxide

In a total of six studies [16,20,23,28,31,32], nitrous oxide was used as a single sedative agent, involving 1547 children using one route of administration: inhalation.

Table 14 shows the population and intervention characteristics, as well as the primary outcomes, sorted by administration route of nitrous oxide. Categories with missing data for all administration routes are not displayed in the table.

### 3.3. Quality Assessment and Primary Studies Overlap

The quality of the included studies [16,17,18,19,20,21,22,23,24,25,26,27,28,29,30,31,32,33] was assessed using the AMSTAR-2 at each item-level; overall confidence was derived according to critical domains, and the related judgments are reported in Supplementary File S3 for each domain. The overall quality of the included studies [16,17,18,19,20,21,22,23,24,25,26,27,28,29,30,31,32,33] resulted as follows: two (11.11% of the included studies) [16,31] high-quality; five (27.78%) moderate [18,20,24,30,32]; five (27.78%) [17,23,26,27,28] low; and six (33.33%) [19,21,22,25,29,33] critically low.

The result of the CCA was 2.6% (67 primary studies without multiple studies, 97 primary studies with multiple studies, and 18 systematic reviews included), calculated based on the Pieper et al. [34] recommendation. The CCA of 2.6% showed a “slight” primary study overlap, according to the overlap grading (0–5% “slight”; 6–10% “moderate”; 11–15% “high”; >15% “very high”) of Pieper et al. [34].

## 4. Discussion

The selection of an ideal sedative agent for sedation is traditionally defined by a set of intrinsic pharmacological requirements: the drug safety, including maintenance of protective airway reflexes and hemodynamic stability; clinical efficacy, in providing behavioral control and sufficient procedural time; and logistical practicality, including rapid onset and a short, predictable recovery profile to facilitate timely patient discharge [35]. Thus, the domains of clinical efficacy, pharmacological safety, and logistically/practicality are inherent to the agent itself.

However, in the clinical variability of sedation in pediatric dentistry, the intrinsic properties of the sedative agents alone may represent only part of the solution. Indeed, pediatric dental sedation relies on several variables that constitute a complex framework, which extends beyond the core pharmacological and pharmacokinetic properties of the agent and the chosen route of administration, also take into consideration the impact of the clinical setting and the type of provider on agent selection; the adequate level of sedation and behavioral control achieved ensuring the actual completion of the dental procedure; and the acceptability and satisfaction of the agent chosen for the pediatric subject and their caregivers.

Therefore, the present umbrella review primarily aimed to provide a structured and critical synthesis of the available knowledge on sedative agents (single agent) and routes of administration employed for achieving sedation (excluding deep sedation and general anesthesia) during dental procedures in pediatric dentistry for behavior management; secondly, to evaluate for each sedative agent, route of administration, and sedation strategy the related acceptability and satisfaction for pediatric subjects, their caregivers, and providers; and furthermore, to assess the influence of the clinical setting (outpatient clinic, dental office, or operating room) and provider type on the selection, route of administration, sedation strategies, and sedation outcomes of sedative agents for achieving sedation during dental procedures.

### 4.1. Sedative Agents in Pediatric Dental Procedures: Anxiolytic–Hypnotic, Sedative–Hypnotic, Opioid Analgesic, Dissociative, and Inhalation Anesthetics

#### 4.1.1. Anxiolytic–Hypnotic Agents: Midazolam, Diazepam, Zolpidem, Triclofos

Among the anxiolytic–hypnotic agents, midazolam was the most investigated, in seven routes of administration: per os (PO) [16,18,20,21,24,25,28,30,31,32,33], sublingual (SL) [16,22,28,32], buccal [20,28,32], intravenous (IV) [16,18,19,20], intranasal (IN) [16,20,22,25,26,27,28,30,32], intramuscular (IM) [16,28,32], and per rectum (PR) [16,21].

The PO formulation was the most frequently employed (*n* = 1398), likely owing to its practical ease of administration and its suitability, especially for children who are needle-phobic or anxious about a more invasive route of administration [36,37]. Moreover, its palatability may also be enhanced by flavoring vehicles, such as sweeteners, fruit juices, or sodium citrate, potentially facilitating acceptance in selected patients [38].

However, oral administration is strictly dependent on pediatric subjects’ cooperation, and the drug may be partially expelled or spat out, particularly in younger or non-compliant children [38,39]. Nevertheless, in the present umbrella review, children with intellectual disabilities or autism received PO midazolam, highlighting its potential value in dental care also for pediatric patients with systemic conditions, who often experience substantial unmet dental needs and reduced access to care, together with heightened procedural anxiety due to communication difficulties or sensory hypersensitivity, frequently resulting in significant behavioral management challenges in the dental setting [40,41]. In this context, pediatric dental sedation may represent a valuable adjunct to promote patient comfort and cooperation, enabling dental providers to overcome behavioral barriers and provide appropriate dental care to a population that is often underserved and at risk of oral health disparities, with PO administration being suitable for children able to reliably ingest and retain the medication, while alternative sedation approaches should be considered when this cannot be ensured.

However, several considerations should be taken into account when using PO midazolam in pediatric dentistry. Compared with other routes of administration of the same agent, PO midazolam required higher dosing, with a mean reported dose of 0.76 mg/kg, compared with 0.23 mg/kg for intranasal (IN) administration and 0.076 mg/kg and 0.5 mg/min for intravenous (IV) delivery.

In fact, oral midazolam undergoes extensive presystemic metabolism, determined by the combined first-pass extraction of the intestinal mucosa and liver via CYP3A-mediated biotransformation [42].

Indeed, age-dependent differences in CYP3A activity result in marked variability in oral bioavailability. For instance, preterm neonates demonstrated higher bioavailability (49–92%) due to immature intestinal and hepatic metabolism, whereas children older than one year showed lower values (21% approximately), with adults exhibiting intermediate bioavailability (30% approximately) [43,44]. Clinically, this maturational increase in first-pass metabolism suggests that older children may need higher weight-adjusted oral doses to achieve sedation compared to younger infants or non-oral routes, since a greater fraction of the dose is metabolized before reaching systemic circulation [44], as currently found in the present umbrella review, when compared to IN or IV administration routes.

Moreover, higher doses of PO midazolam are associated with longer recovery times [45], which in the present umbrella review averaged 108.62 min. Such prolonged recovery times require careful post-dental procedure monitoring, which may not be feasible in all dental settings. In the included studies, PO midazolam setting was specified for 690 subjects and was administered in outpatient dental clinics (653/690 subjects, 94.64%) and dental offices (21/690, 3.04%), potentially reflecting contexts in which adequate monitoring was ensured. The higher dosing, variable absorption, and extended recovery may add a logistical burden within the dental setting and underscore the need for careful observation, particularly given the observed adverse events, including oxygen desaturation (*n* = 50), which was, however, managed through oxygen administration.

Conversely, IV midazolam, administered to 256 subjects in the included studies, demonstrated a faster mean onset of sedation (8 min vs. 15.5 min for PO) and a shorter mean recovery time (15.78 min). This reflects the pharmacokinetic advantages of IV administration, which bypasses presystemic metabolism, resulting in complete systemic bioavailability (100%), and allowing rapid distribution to the central nervous system, followed by relatively fast redistribution and elimination [46,47]. These characteristics account for the predictability of sedation onset and the shorter post-procedural recovery observed. However, intravenous administration is more invasive as it requires venous access, continuous monitoring, and properly trained personnel, which should be weighed against its potential pharmacokinetic benefits [46].

Indeed, in the present umbrella review, compared to other routes of administration, dental procedures using IV midazolam were performed primarily by anesthesiologists, specified for 279 subjects (234/279 subjects, 83.87%). Although IV midazolam achieved favorable levels of behavioral control (mean Houpt score 5.8), numerically higher than those registered to other administration routes, and although no quantitative comparison was performed, an apparent “intravenous paradox” was observed. Specifically, despite its behavioral control, the rate of dental treatment completion was specified as 194 subjects, found to be lower with IV midazolam (104/194, 53.61% completed; 90/194, 46.39% non-completed) compared to with PO midazolam, investigated for 302 subjects (284/302, 94.04% completed; 18/302, 5.96% non-completed). This apparent discrepancy may be related to the increased monitoring requirements associated with IV sedation, which can potentially delay or interrupt procedures in the case of adverse reactions or complications. In the present umbrella review, such events were relatively infrequent, including mainly nausea, vomiting, vertigo, sleepiness/fainting, and sore mouth. However, given the intra-individual variability that may occur with IV sedation, some patients may potentially require airway support or additional interventions, underscoring the importance of monitoring in this context [48], which may affect dental procedure completion.

Similarly, IM midazolam, which also avoids gastrointestinal first-pass metabolism and is rapidly absorbed with a bioavailability exceeding 90%, may share the practical limitations associated with an invasive route despite favorable pharmacokinetics [44]. However, unlike the more thoroughly investigated profile of IV sedation, several instances of missing data on dental procedure completion, sedation, or behavior score, as well as acceptability and satisfaction, are present in the present umbrella review, potentially limiting thorough clinical evidence and interpretation within pediatric dental sedation.

In this context, IN midazolam may emerge as a clinically balanced alternative, potentially combining pharmacokinetic efficiency with practical feasibility in pediatric dental settings. Across the included studies, IN midazolam was administered to 1026 children at a mean dose of 0.23 mg/kg, positioned between oral (0.76 mg/kg) and intravenous regimens (0.076 mg/kg, 0.5 mg/min). By bypassing first-pass hepatic metabolism, the intranasal route allows for direct systemic absorption, resulting in improved bioavailability and a predictable sedative effect [49], with a reported average recovery time of 37.5 min, intermediate between that of PO and IV administration. This recovery profile may likely reflect a more incline pharmacodynamic profile to dental procedures, providing sufficient duration to stabilize anxiety and behavior throughout the procedure, as indicated by an intermediate Houpt score achieved of 4.09, without the prolonged or variable sedation often associated with oral administration, nor the invasiveness and technical demands of IV sedation. Clinically, IN midazolam demonstrated high procedural completion, with low non-completion rates, investigated in 220 subjects (5/220, approximately 2.27%), and predominantly mild, self-limiting adverse effects. Its needle-free delivery likely enhances acceptability and satisfaction among children and caregivers, as currently reported, particularly for pediatric subjects whose cooperation for oral intake cannot be reliably ensured [50]. From this perspective, IN midazolam may be interpreted as a practical compromise that balances sedation levels, duration, and clinical practicability with the operational demands of pediatric dental interventions, particularly when PO administration is not indicated due to cooperation challenges, and more invasive approaches, such as IV administration, may be disproportionate to the dental procedure’s complexity and treatment duration.

Transmucosal routes, including SL and buccal administration (currently administered at a mean of 0.3 and 0.27 mg/kg, respectively), allow absorption through the oral mucosa, potentially providing faster onset of sedative effects than oral administration [51]. In the umbrella review, SL midazolam reported favorable behavioral outcomes (Houpt 5.6 among 40 subjects) and acceptance, although procedure completion was not documented; buccal administration showed intermediate behavioral control (with a mean Houpt of 3.46; and score range of 3–4), with a completion rate, specified in 45 subjects, of 42/45 (93.33%) and non-completion of 3/45 (6.67%). Unlike IM or IV administration, these routes might facilitate acceptance and cooperation with administration, provided that children can follow instructions and keep the medication in place during administration.

Indeed, the effectiveness of transmucosal administration is influenced by mucosal residence time and salivary dynamics. The bioavailability of midazolam after buccal administration increases with prolonged exposure to the mucosa [52,53], whereas high salivary flow or involuntary swallowing may wash away the drug, reducing systemic absorption and potentially decreasing sedative efficacy; conversely, children able to maintain the formulation sublingually or buccally might achieve more predictable sedation [52,53].

Furthermore, transmucosal drug administration may serve as an adjunct to more-invasive routes such as IV sedative agents, particularly when systemic conditions require IV-related intense monitoring, or more complex dental procedures are needed. Although the present umbrella review considered only single sedative agents, the combined use of non-invasive methods, such as sublingual or buccal midazolam, may support IV administration. For instance, an effective premedication method using sublingual midazolam delivered through a suction toothbrush was reported, enabling subsequent procedural steps with minimal distress [54]. Importantly, this approach may also be translated to pediatric patients, as the possibility of self-administering the medication, rather than receiving it directly from a provider, could simplify administration and enhance compliance, giving the child a sense of control over the procedure.

Among other sedative–anxiolytic agents, diazepam was used with PO and PR administration. PO diazepam was administered at a mean dose of 0.42 mg/kg and resulted in lower behavioral control (mean Houpt score of 4.5) compared to oral midazolam.

Moreover, PR administration of both diazepam and midazolam was primarily reserved for very young children (mean ages of 2.67 years and 3.61 years, respectively), likely reflecting the relative ease of administration in populations less able to cooperate with other administration routes. Although reported data were limited, the number of reported successful sedations for PR diazepam and midazolam potentially suggests that rectal routes can provide effective sedation in this age group, despite occasional challenges reported in completing dental procedures, specified for 299 subjects (89/299, 29.77% in PR midazolam).

Among non-benzodiazepine anxiolytic–hypnotic agents, zolpidem and triclofos were limitedly investigated in the present umbrella review, administered in 35 and 45 subjects, respectively, and delivered by anesthesiologists. Both agents showed no adverse effects, and the mean sedation score was 6.47 for zolpidem and 5.00 for triclofos, based on an eight-point sedation scale.

The available evidence concerning these agents mainly derives from non-dental settings, where PO administration of zolpidem and triclofos has been investigated for preoperative anxiety and facilitation of immobility during diagnostic or procedural interventions, showing adequate sedative efficacy, good tolerability, and a low incidence of adverse events, but limited impact on active behavioral modulation compared with benzodiazepines [55,56,57,58].

When translated to pediatric dental sedation, however, their clinical manageability might likely appear more limited than that of other sedative agents, including benzodiazepines. Indeed, although a short-acting and rapid onset characterizes zolpidem, and triclofos provides longer-lasting sedation [57], their maneuverability might pose more difficulty in dental settings. While zolpidem, like midazolam, can be pharmacologically antagonized by flumazenil [59], triclofos does not have a specific reversal agent, which may reduce the ability to manage sedation complications and limit its use to settings with appropriately trained personnel and structured monitoring. However, the limited data on pediatric dental sedation may limit the possibility of definitive conclusions regarding its practical applicability in the dental setting.

#### 4.1.2. Non-Benzodiazepine Sedative–Hypnotic Agents: Dexmedetomidine, Chloral Hydrate, Promethazine, Hydroxyzine, Phenobarbital

Among non-benzodiazepine sedative–hypnotic agents, dexmedetomidine has been the most extensively investigated, exhibiting multiple routes of administration, including PO, IN, SL, and IV. Dexmedetomidine is a highly selective, short-acting α2-adrenoceptor agonist, capable of inducing sedation, anxiolysis, analgesia, antisialagogue effects, and sympatholysis, with minimal respiratory depression, and exhibits mild affinity for non-adrenergic imidazoline receptors, contributing to its central effects [60,61,62].

In the present umbrella review, PO dexmedetomidine was administered to 134 children, while IN administration was reported in 410 children, and IV in 30. PO dexmedetomidine, similar to midazolam, is often preferred over IV or IN routes, for ease of administration; however, its onset is slower, recovery is prolonged, and bioavailability is limited due to high hepatic first-pass metabolism, compared with SL or IN administrations, also considering the potential gastrointestinal adverse effect often related to PO delivery [60]. However, no such events were reported in the present umbrella review, except for a single episode observed after IN administration.

Dexmedetomidine is characterized by being highly lipophilic at physiological pH (pKa 7.1), thereby potentially facilitating rapid tissue penetration [60,63]. Especially when administered IN or SL, absorption is rapidly achieved due to the rich vascularization of the nasal mucosa and proximity to the central nervous system, via the olfactory pathway, leading to faster onset and more-predictable sedation compared with the oral route [60,63]. Moreover, IN administration is non-invasive, avoids the bitter taste of oral formulations, and is better accepted in children [60]. Indeed, in this umbrella review, self-reported acceptance of IN dexmedetomidine, although limitedly investigated, ranged from “fair to excellent” and “well accepted”, highlighting potentially favorable acceptability outcomes within pediatric populations.

Beyond achieving sedation and anxiolysis, dexmedetomidine is characterized by analgesic effects via central α2-adrenergic stimulation [60]. Evidence on adult dental procedures has shown the role of this drug in prolonging postoperative analgesia, with reduction in VAS scores, and through vasoconstriction properties, reduced intraoperative bleeding during dental surgeries [64]. Compared to other sedative agents, which mainly exert sedative and behavioral control/anxiolytic effects [60], dexmedetomidine may offer, in certain clinical situations, broader support, particularly when analgesia or hemodynamic stability is desirable.

IV administration was explored in 30 children, administered at lower doses (mean 1.7 μg/kg), compared to PO (4.10 7 μg/kg), similar to midazolam. It should be noted that dexmedetomidine IV delivery requires slow titration due to its potential biphasic blood pressure effects [60]. Nevertheless, no adverse hemodynamic events or other complications were presently reported. However, evidence has demonstrated that both the sedative and hypotensive effects of dexmedetomidine can be reversed with intravenous atipamezole when necessary [60,65].

The other non-benzodiazepine sedative–hypnotic agents presently reported are PO chloral hydrate, promethazine, hydroxyzine, and IM phenobarbital; however, the available evidence for these agents was limited by several instances of missing data, which may restrict comparisons and the overall interpretation of the findings.

Chloral hydrate was used in 149 pediatric subjects, with a mean dose of 60.69 μg/kg and 0.8–1.0 mL/μg. Indeed, dosage selection is critical, as the effects are dose-dependent; at higher doses, there is a risk of exceeding minimal or moderate sedation and progressing to deep sedation or general anesthesia, with potential depression of the respiratory and vasomotor centers [66,67]. Furthermore, even at therapeutic doses, the wide inter-individual variability in response could lead to complications, including tongue muscle weakness, possible retraction towards the oropharynx, and respiratory complications [67,68]. Therefore, careful monitoring and expertise from the providers are necessary, as well as an adequately equipped delivery setting, given the evidence that complications or adverse events occur in non-hospital-based settings, making it essential for dental providers to be capable of pediatric advanced life support to cope with emergencies [68]. Given these circumstances, despite its effectiveness in providing successful sedation, other sedative agents, including midazolam, have increasingly replaced the use of chloral hydrate [68].

Other sedative–hypnotic drugs included in this umbrella review as single PO agents are promethazine and hydroxyzine, whereas IM administration involved phenobarbital. However, the available evidence for these medications remains limited, as their use is predominantly reported in combination with other sedative agents, such as midazolam, meperidine, chloral hydrate, or ketamine [69,70]. Consequently, the paucity of data on sedation effectiveness, completion of dental procedures, and findings on acceptability and satisfaction limits the ability to draw definitive conclusions about the role in sedation in pediatric dental practice.

#### 4.1.3. Opioid Analgesics: Tramadol, Meperidine, Sufentanil

Opioid analgesic drugs were limitedly represented as single sedative agents in the present umbrella review, with most findings deriving from small sample sizes and heterogeneous reported data.

Meperidine was administered intramuscularly in 45 pediatric patients at a mean dose of 1.08 mg/kg and was associated with one interrupted dental procedure due to unmanageable behavior. This finding is consistent with the known pharmacodynamic profile of meperidine, which, despite producing analgesia and drowsiness, may be associated with dysphoria, mood alterations, and behavioral agitation [69]. Such effects may partly explain why, during dental procedures, meperidine was scarcely found as a single agent within the studies presently found, as it could be more frequently used in combination with other central nervous system depressants, such as promethazine or midazolam, to enhance sedative efficacy and behavioral control [69].

Similarly, tramadol was evaluated as a single oral agent in a sample of 15 children, administered at a mean dose of 2 mg/kg, yielding a moderate sedative effect (mean sedation score of 4.07 on an eight-point scale). However, the limited sample size and the absence of additional behavioral or procedural outcomes substantially restrict interpretation. Moreover, the available literature suggests that tramadol is more frequently investigated and employed for its analgesic properties rather than as a primary sedative, particularly in dental and oral surgical contexts, such as third-molar extraction, where its role appears to be predominantly pain control rather than behavioral sedation [71,72]. This may further explain its limited adoption as a sole sedative agent in pediatric dentistry.

Intranasal sufentanil was reported as a single agent in a cohort of 10 children, with a mean dose of 1.25 µg/kg and a satisfactory sedation score of 5.5 on a 10-point scale. Notably, this dosage is comparable to that reported in the broader literature, in which IN sufentanil, owing to its high lipophilicity and favorable bioavailability, has demonstrated rapid absorption, stable hemodynamics, and an acceptable safety profile [73]. Nevertheless, most available evidence supports its use within multidrug sedation strategies rather than as a single agent, likely to optimize sedative depth while minimizing opioid-related adverse effects, particularly respiratory depression [73]. Although reversal is managed through naloxone, careful dosing and monitoring remain essential when opioids are administered to pediatric patients [73]. Interestingly, while satisfaction data were not reported for IN sufentanil in the present umbrella review, evidence has described high operator satisfaction in combination strategies, potentially supporting its valuable role in sedation protocols [73].

#### 4.1.4. Dissociative and Inhalation Anesthetics: Ketamine, Nitrous Oxide

Among the dissociative anesthetic agents reported, ketamine was administered PO in 106 pediatric patients and IN in 253. As for other agents, comparing between PO and IN routes, IN ketamine required a lower mean dose to achieve sedation than PO administration (4.74 mg/kg vs. 8.71 mg/kg, respectively), and a markedly shorter onset time (mean 9.58 vs. 21.11 min).

Ketamine induces a dissociative state, characterized by functional neurophysiological separation between cortical and limbic activity, which leads to profound analgesia and amnesia, while preserving spontaneous respiration and cardiovascular stability, a state known as catalepsy [69,74]. Clinically, patients are awake, with open eyes, and may exhibit involuntary movements, yet remain disconnected from environmental stimuli [69,74]. These intrinsic pharmacodynamic properties likely explain the favorable sedation reported and behavioral outcomes, with positive behavior scores across multiple scales, including five-point behavior scales, FLACC, and overall movement–crying–behavior assessments.

The recurrence of adverse events was investigated for 58 subjects, which mainly included: vomiting (5/58, 8.62%, PO; 1/58, 1.72%, IN), paradoxical reactions (1/58, 1.72%, PO), hallucinations (5/58, 8.62%, PO), oxygen desaturation or hypoxemia (3/58, 5.17%IN), and emergency reactions (2/58,3.45%, PO). These findings are consistent with previous evidence, where emesis was frequently reported as a side effect of ketamine, particularly in younger children, suggesting a possible age-dependent vulnerability [74,75]. Importantly, involuntary or sudden muscle spasms, rigidity, or hypertonicity are well-recognized effects of ketamine and should not be interpreted as an inadequate level of sedation or analgesia [69,74]. The frequent reporting of these effects underscores the importance of operator experience in correctly interpreting clinical signs and managing with dissociative sedation. Indeed, ketamine administration is generally recommended within adequately equipped settings, especially hospitals, and under the supervision of experienced personnel [74]. Consistently, in the present umbrella review, ketamine was predominantly administered by anesthesiologists or by multidisciplinary teams including anesthesiologists and dentists, and was reported exclusively within university hospital environments, which may further suggest that sedation with ketamine, despite being effective, requires appropriate expertise levels and infrastructure.

Nitrous oxide (N_2_O) has been extensively investigated in this umbrella review, which included a total of 1547 subjects. The inhalation route is commonly used in pediatric dentistry, either as a single agent or in combination with other drugs, due to its anxiolytic and modest analgesic properties, rapid onset and recovery, ease of use, and the safety and non-invasiveness of administration [76].

N_2_O has been used in various N_2_O/O_2_ concentrations, most frequently in a 30/70 ratio, followed by 40/60 and, in limited cases, 50/50. The AAPD suggests that administration can be done by standard titration, which means introducing 100% oxygen for 1–2 min, followed by gradual titration of nitrous oxide in 10% increments, or by rapid induction, with administration of a fixed dose or a predetermined percentage without titration [76]. Evidence suggests that a 30/70 ratio is often sufficient to achieve anxiolysis and analgesia, avoiding exceeding 50/50 to minimize the risk of adverse events [76]. Moreover, concentrations can be adapted to the dental procedure: lower for shorter, less complex procedures; and higher for longer or potentially more stressful procedures, such as extractions or local anesthesia administration [76].

The effects of nitrous oxide were reported as rapid, with an average onset of approximately 6 min (range 2–18). However, the peak effects depend on the child’s cooperation, which is why it is advisable to continue verbal behavioral management throughout the procedure to ensure effective nasal breathing [76]. Patient selection must therefore be carefully considered, as in some subjects, for example, children with disabilities or who have difficulty cooperating, nitrous oxide alone may not be sufficient [76]. Despite this, in the present review, N_2_O was used as the sole agent even in subjects with intellectual disabilities (*n* = 472/1547).

Overall, it was reported to be effective in sedation, with adequate behavioral control (mean Houpt score of 5.02), very close to that achieved with PO midazolam (Houpt of 5.09), but with the potential advantage of a faster onset and recovery time. The main reported adverse events might be related to the expansion of gas in air-containing body spaces [76]. The recurrence of adverse events was investigated for 546 subjects, with nausea and vomiting reported in 28 (5.13%) and 14 subjects out of 546 (2.56%), respectively, while 382 (69.96%) subjects experienced no adverse effects. Other reported effects included headache (53/546, 9.71%), drowsiness/fainting (20/546, 3.66%), and isolated cases of epistaxis, hiccups, or sore mouth.

N_2_O enabled dental procedures to be completed, specified for 804 subjects as being completed in 633/804 cases (78.73%), while in 171/804, 21.27%, the dental procedure was not completed, of which 2 were due to refusal of the procedure and 44 due to lack of cooperation. Uncooperative behavior by the child can compromise the effectiveness of N_2_O and, consequently, the dental procedure itself; for this reason, it is essential that the professional actively manage the patient’s behavior, avoiding crying, refusal of the mask, or mouth breathing [76]. In addition, nitrous oxide was currently associated with high levels of satisfaction among both children and caregivers. These results are consistent with other evidence that parents generally perceive nitrous oxide sedation as safe and beneficial for dental treatment, preferring it to more invasive or deeper sedation methods [77]. Based on the synthesized evidence, nitrous oxide appears to be consistently reported as an effective and well-accepted sedative agent in appropriately selected cooperative patients, thanks to its ease of administration, rapid recovery, low adverse event profile, behavioral control, and high acceptability by children and caregivers. Conversely, in patients with poor or no cooperation, with systemic disease requiring careful monitoring, or needing more invasive or prolonged dental procedures, the use of other sedative agents, used alone or in combination, may be indicated to ensure adequate behavior control and procedural safety and completion.

### 4.2. Impact of the Provider on the Sedative Agent Choice

A clinically relevant aspect that emerged from this umbrella review concerns the provider’s level of expertise and its possible impact on the choice of sedative agent and route of administration [78]. The present findings show a potential association between the type of drug used and the professional figure responsible for sedation. Indeed, the providers who performed sedation were specified for 1765 procedures as follows: dentists for 824, anesthesiologists for 679, anesthesiologists and dentists for 150, specialists trained in pediatric sedation and life support for 42, dental nurses for 20, nurses for 20, dental assistants for 16, and pediatric dentists for 14.

**Figure 3:**
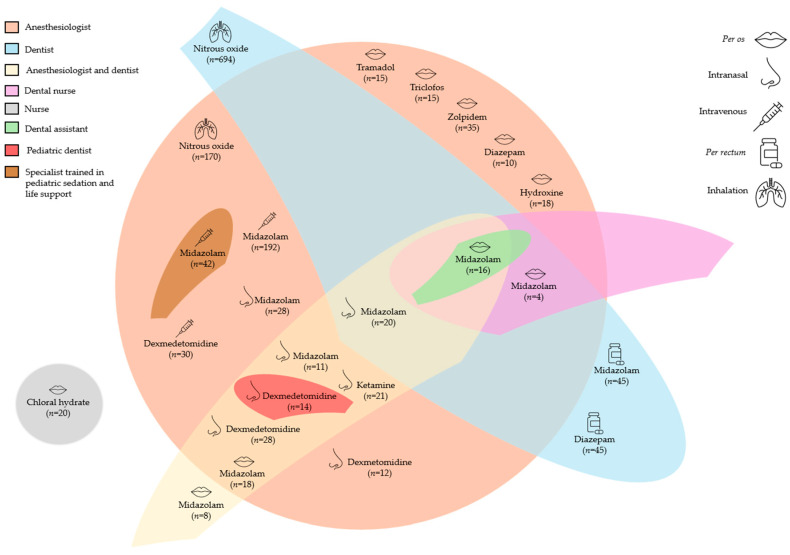
Provider distribution sorted by sedative agents and routes of administration.

Figure 3 illustrates the distribution of providers according to the sedative agents and routes of administration employed.

Most anxiolytic–hypnotic agents, including midazolam, diazepam, zolpidem, and triclofos, were administered by anesthesiologists or by multidisciplinary teams comprising anesthesiologists and dentists via any route of administration. Only occasionally was the administration of midazolam and diazepam by the rectal route (*n* = 45) or of diazepam by the intranasal route by dentists reported, while intravenous administration of midazolam was in some cases performed by specialists trained in pediatric sedation and advanced life support (*n* = 42). Less frequently, these drugs were administered by support staff, such as dental assistants or nurses, with PO midazolam used in 16 and 4 pediatric subjects, respectively.

A similar trend was observed for non-benzodiazepine sedative–hypnotics, including dexmedetomidine, chloral hydrate, promethazine, hydroxyzine, and phenobarbital, as well as for opioid analgesics such as tramadol, meperidine, and sufentanil, which were mainly administered by anesthesiologists. Only in limited cohorts were these drugs managed by pediatric dentists, as in the case of IN dexmedetomidine (*n* = 14), or by nursing staff, as in the case of PO chloral hydrate (*n* = 20).

For dissociative and inhalation anesthetics, ketamine was administered mainly by anesthesiologists or dentists in highly controlled settings (*n* = 21). In contrast, nitrous oxide showed an opposite trend, being the sedative agent most frequently used by dentists, as it was administered to 694 pediatric subjects by dentists, compared to 170 by anesthesiologists. This distribution may reflect the widespread use of nitrous oxide as a sedative agent in pediatric dental practice, likely due to its established safety profile, non-invasiveness, and ease of use by dentists [74].

However, the wide variability observed in the distribution of providers highlights how the education and training of professionals is an essential element in sedation practice [48,79]. Providers performing sedation should have advanced skills in pre-sedation assessment and appropriate patient selection, considering individual characteristics, level of cooperation, the presence of comorbidities, drug therapies, and the type of procedure to be performed, to achieve efficacy and safety [79]. Moreover, in-depth knowledge of the physiology and pathophysiology of the airways and the respiratory and cardiovascular systems, as well as the pharmacological principles of the sedatives and analgesics used, including indications, dosages, contraindications, drug interactions, reversal agents, and adverse events management, is also essential [79]. In addition, the provider should be able to protect the emotional and psychological well-being of pediatric patients by recognizing and managing anticipatory anxiety, fear, and expectations, and adopting communication and comfort strategies to create a reassuring and collaborative environment, as sedation success does not depend solely on the depth of sedation, but also on the ability to integrate pharmacological agents with appropriate behavioral management, optimizing patient comfort and efficiency [7,48,79].

In addition, the professional must be trained in the early recognition of sedation-related complications and their management, including the ability to use monitoring systems correctly, to intervene in the event of respiratory or cardiovascular compromise, and to use antagonistic drugs when indicated [48,79]. Knowledge of airway management techniques and basic and advanced resuscitation maneuvers is a fundamental prerequisite for ensuring patient safety [79]. These factors help explain why, in this umbrella review, some sedative drugs were mainly administered by anesthesiologists or in contexts of close multidisciplinary collaboration, especially for medications associated with a higher risk potential, likely due to the insufficient knowledge of dentists alone. At the same time, even for agents commonly used in dental practice, such as nitrous oxide, and associated with a relatively low risk profile, structured training of the dentist in sedation, basic life support, and emergency management should be, in any case, essential to ensure high standards of safety, efficacy, and quality of care for the pediatric subjects.

### 4.3. Impact of the Setting on the Sedative Agent Choice

This umbrella review also highlighted the impact of the clinical setting on the choice of sedative agent and method of administration. The selection of the sedative drug cannot be separated from the clinical context in which the procedure is performed, as it is essential to ensure adequate monitoring of vital signs and to have resources proportionate to the route of administration, the pharmacological profile of the agent used, and the expected level of sedation, as some drugs require more intensive monitoring and the immediate availability of equipment and trained personnel to manage potential complications [48,79].

**Figure 4:**
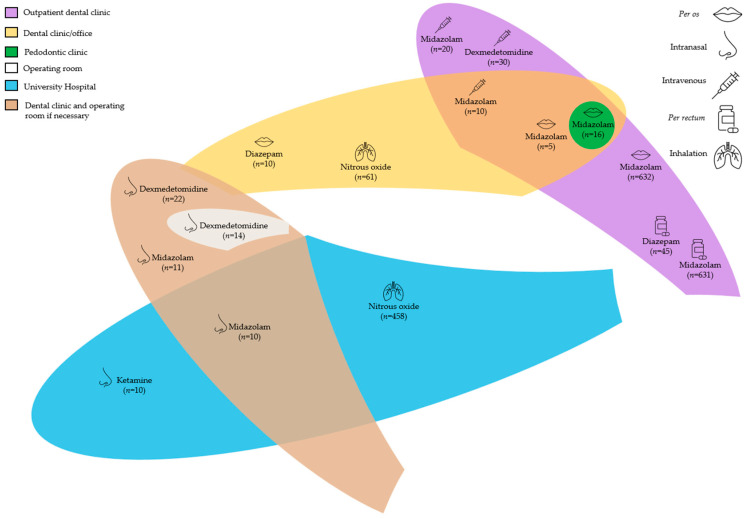
Setting distribution sorted by sedative agents and routes of administration.

Figure 4 shows the setting distribution according to the sedative agents and routes of administration employed.

The clinical setting in which the sedation was performed was specified for 2056 procedures as follows: an outpatient dental clinic for 1389 pediatric subjects, a university hospital for 478, a dental clinic/office for 102, a dental clinic and operating room transfer if necessary for 57, a pedodontic clinic for 16, and an operating room for 14.

In particular, anxiolytic–hypnotic agents, including midazolam and diazepam, were primarily administered in dental clinics, with the possibility of transfer to the operating room, whereas their use in dental offices was reported only in limited cases. Non-benzodiazepine sedative–hypnotics, such as dexmedetomidine, were mainly used in dental clinics and university hospital settings, consistent with the need for more structured monitoring [14,79]. Ketamine, as a dissociative anesthetic, associated with a higher risk profile [75], has been administered mainly in university hospitals, where advanced resources for monitoring and airway management might be available.

Nitrous oxide was administered both in university hospitals and, more frequently, in clinics and dental practices, confirming its potential wide applicability in different care settings. This versatility might be related to its favorable safety profile, the agent’s titratability, and the potential for less-intensive monitoring compared to other sedative drugs.

The currently observed difference concerning the impact of clinical setting and provider type may likely reflect case-mix and dental procedure complexity; however, the present umbrella review does not support causal interference; therefore, further studies may be needed to clarify potential causal relationships.

### 4.4. Dental Procedures Implications and Management of Pediatric Subjects

In this umbrella review, sedation was generally reported as an effective strategy in facilitating the completion of dental treatment in children. Specifically, the types of dental procedures performed were specified for 3674 procedures as follows: tooth extraction (*n* = 1048), restorative dental therapy (*n* = 846), oral examination (*n* = 724), non-specified oral surgery (*n* = 584), and oral hygiene (*n* = 472). Within this complex procedural context, sedation has shown a good level of clinical efficacy: there were 1857 cases of sedation being reported as effective, while completion of dental treatment was documented in 1929 cases, with 1551 procedures completed without difficulty and a further 89 completed with difficulty. In contrast, 289 children did not complete treatment. When the cause of discontinuation was specified, lack of cooperation was the main limiting factor (*n* = 45), followed by inability to tolerate the sedative agent (*n* = 5), refusal of the procedure (*n* = 2), and paradoxical reactions (*n* = 1). These data suggest that, even with pharmacological support, pediatric patient cooperation remains a key determinant of treatment success.

These results are consistent with those reported in the literature, where dental procedures performed with pharmacological support generally show higher completion rates than exclusively non-pharmacological approaches, particularly for restorative and extractive treatments [80], although direct comparative conclusions cannot be drawn within the present synthesis. In particular, pharmacological interventions, including sedation and general anesthesia, are more frequently associated with treatment completion, especially in younger patients and those undergoing painful or invasive procedures, while non-pharmacological strategies are more appropriate for less complex treatments and in more cooperative children [80,81,82].

However, the data from this umbrella review emphasize that sedation cannot be considered independent of the child’s cooperation, especially for certain administration routes [76]. Delivery methods such as inhalation via a mask, oral, or sublingual administration require a minimum level of initial cooperation in order for the drug to be administered effectively and achieve a level of sedation adequate to proceed [83]. This aspect is particularly relevant in the pre-sedation phase, before the pharmacological effects are fully established, and helps explain why lack of cooperation is the main cause of procedure interruption even in the presence of sedation [80]. In this perspective, integrating sedation with brief desensitization strategies and familiarization with the dental environment, the operator, and the administration devices may facilitate treatment acceptance and improve clinical outcomes. As reported in the literature, adequate management of anticipatory anxiety and dental fear reduces the risk of treatment discontinuation and promotes a more positive dental experience in the long term [80].

Sedation therefore emerges as a potentially valuable aid in the management of dental procedures traditionally associated with high anxiety and requiring cooperation, such as extractions and restorative therapies [84], which constitute the majority of the procedures included in the review. These treatments require not only tolerance of local anesthesia, but also prolonged maintenance of oral opening, placement of a rubber dam, and exposure to potentially anxiety-provoking dental instruments, all of which can lead to fear, refusal, or interruption of the procedure in the absence of adequate sedative support [4,85].

While recognizing the need for contingent cooperation for certain modes of administration, sedation still represents a clinically and economically advantageous alternative to general anesthesia [86]. As also highlighted by previous studies, conscious sedation techniques, particularly oral or inhalation methods, reduce the invasiveness of treatment, overall costs, recovery times, and the need for an advanced hospital setting, while maintaining a high probability of treatment completion in anxious pediatric patients or those who are difficult to manage with non-pharmacological techniques alone [80].

Therefore, further studies are required to analyze the most effective sedative agents and the routes of administration used to achieve sedation during pediatric dental procedures, as well as to determine which sedation strategies are more acceptable and satisfying to pediatric patients, their caregivers, and healthcare providers. Additionally, future research should further explore the influence of the clinical setting on sedation selection, delivery, and outcomes in pediatric dentistry.

### 4.5. Limitations, Unaddressed Knowledge Gaps, Strengths

#### 4.5.1. Limitations

In the critical interpretation of the findings, some limitations of the present umbrella review should be recognized. In particular, the AMSTAR-2 tool showed that even among the included systematic reviews, about 56% had a high [16,31] or moderate [18,20,24,30,32] quality, and the remaining 44% had a low [17,23,26,27,28] or critically low quality [19,21,22,25,29,33]. This could signify that potential biases could reduce the robustness of the conclusions. Based on the quality assessment (Supplementary File S3), the most common methodological weaknesses found in the included systematic reviews were:

- The lack of reporting about the sources of funding of the included studies (domain 10 of the AMSTAR-2). Previous evidence has shown that studies supported by commercial sponsors were more likely to present results in favor of the sponsors compared to unfunded studies [87]. The lack of this information may compromise the possibility of assessing potential conflicts of interest.
- The lack of a pre-registered study protocol or of an explicit statement that the materials and methods of the systematic review were defined prior to conducting the study (domain 2 of the AMSTAR-2). This is a critical domain of the AMSTAR-2, and this lack raises doubts concerning potential reporting biases.
- The lack of well-defined PICO components in the research questions and inclusion criteria (domain 1 of the AMSTAR-2). An unclear or incomplete definition of the PICO components may limit the study’s transparency and reproducibility, or can lead to heterogeneous study selection, making it difficult to interpret and compare results [87].

Probably as a consequence of the potentially heterogeneous study selection performed in the included systematic reviews, the findings of the present umbrella review showed substantial heterogeneity. Specifically, the included systematic reviews differed widely in terms of reported variables and investigated outcomes, particularly regarding the sedation/behavior scale used, as well as in the routes of administration and types of sedative agents considered. Given the broad range of sedative agents examined (14 in total) and the variability in administration routes (eight in total), the synthesized evidence appears fragmented, which hindered the possibility of conducting a meta-analysis.

#### 4.5.2. Unaddressed Knowledge Gaps

A pre-specified secondary objective of this umbrella review was to synthesize evidence regarding acceptability and satisfaction among children, caregivers, and providers of the different sedative agents, due to the scarcity of current data available in the literature. However, the included systematic reviews rarely reported these outcomes in a structured or comparable manner. Rather than representing a methodological weakness of the present umbrella review, this finding highlights a significant evidence gap in the pediatric dental sedation literature. The a priori definition of this objective, as specified in the protocol and before starting the research, allowed us to systematically document this under-reporting and emphasize the need for future primary studies to incorporate standardized measures of acceptability and satisfaction.

To address the identified reporting gaps and the heterogeneity observed across the literature, a minimum reporting set was proposed in the present umbrella review (Table 15). Table 15 defines the essential items that should be systematically reported in future pediatric dental sedation research. The adoption of these standardized criteria is intended to ensure greater transparency and comparability, facilitating robust evidence synthesis and providing clearer guidance for clinical practice.

#### 4.5.3. Strengths

Despite the absence of quantitative synthesis the results of the present umbrella review were strengthened by: consistent patterns across high-volume agents (midazolam and nitrous oxide as the most studied/safe/effective in appropriate settings); large cumulative sample (6877 children from 97 primary studies); low primary-study overlap (CCA 2.6%); methodological rigor via PROSPERO registration, PRISMA compliance, dual extraction, and AMSTAR-2 assessment (with transparent reporting of quality limitations) and alignment with established guidelines restricting single-agent conscious sedation to monitored, non-deep levels. These strengths are detailed and analyzed below.

Based on the quality assessment (Supplementary File S3), the most common methodological strengths found in the included systematic reviews were:

- A clear definition of the selection of the included studies in the systematic review, based on the study’s design (domain 3 of the AMSTAR-2), which improved the transparency and reproducibility.
- A comprehensive description of the included studies in adequate detail (domain 8 of the AMSTAR-2), which improved the transparency and interpretability of the reported data.
- A satisfactory technique in the risk of bias assessment of the included studies (domain 9 of the AMSTAR-2) using a validated tool. This is a critical domain of the AMSTAR-2, which strengthened the reported evidence and related conclusions.

Moreover, to the best of our knowledge, the present umbrella review provides the first comprehensive umbrella-level synthesis focused exclusively on single sedative agents and routes of administration employed for achieving sedation (excluding deep sedation and general anesthesia) during dental procedures in pediatric dentistry, assessing also the influence of the clinical setting (outpatient clinic, dental office, or operating room) and provider type on the selection, route of administration, sedation strategies, and sedation outcomes of sedative agents. Interestingly, although no date restrictions were applied in the search strategy, the majority of included systematic reviews (10 of the 18 included) were published between 2020 and 2025. This likely reflects the growing clinical and research interest in pediatric sedation, possibly driven by evolving regulatory standards, greater emphasis on patient safety, and methodological standardization in evidence-based dentistry.

In addition, it should be noted that the overall large sample size of children included (6892), the several single sedative agents used (14 in total), as well as the different routes of administration (eight in total), ensured a comprehensive analysis of sedation in pediatric dentistry with single sedative agents. In fact, this umbrella review addresses a specific and previously unaddressed inconsistency in the pediatric dental sedation literature: the absence of a higher-level synthesis focused exclusively on single-agent conscious sedation (excluding deep sedation/general anesthesia and multidrug regimens), with explicit evaluation of the influence of provider type and clinical setting. While multiple systematic reviews have examined sedation agents/routes in pediatric dentistry [16,17,18,19,20,21,22,23,24,25,26,27,28,29,30,31,32,33], they vary substantially in scope (e.g., some include combinations, others focus on single agents only, or include deep sedation), inclusion criteria, outcome evaluated, and reporting of key safety elements (e.g., adverse events often undefined, monitoring inconsistently detailed). By synthesizing 18 systematic reviews encompassing 97 primary studies and 6877 children, while restricting the scope to single-agent regimens and narratively addressing provider/setting influences, this review provides the first comprehensive overview to guide agent/route selection and highlights critical reporting gaps for future standardization.

Furthermore, despite the large samples, several potential biases were excluded a priori thanks to an adequate methodology strategy, which excluded: adult subjects (>18 years of age); deep sedation and general anesthesia; sedation administered prior to general anesthesia procedures; premedications; and not using single sedative agents.

While the exclusion of combined sedative agents represented a methodological strength of the present umbrella review, as it allowed for a clearer evaluation of the effects of single sedative agents without introducing additional sources of bias or heterogeneity, future research should aim to comprehensively assess sedation protocols involving combinations of sedative agents. Comparing the findings of the present umbrella review with future evidence on combined regimens may help identify potential synergistic effects, improvements in efficacy or safety, and combinations that should be avoided in pediatric dental sedation.

Finally, although the primary studies overlap across systematic reviews, which is an acknowledged potential bias in umbrella reviews, the degree of overlap observed in the present study was low, with a CCA of 2.6%. This indicates that only a limited proportion of primary studies were shared across reviews, reducing the likelihood of redundancy and suggesting that the overall conclusions were not substantially influenced by overlapping data [34].

## 5. Conclusions

The present umbrella review suggests that conscious sedation with single agents is consistently reported as an effective approach for behavioral management of patients in pediatric dentistry, with the most-consistent evidence emerging for a limited number of agents and administration routes.

Based on the current synthesized evidence, Midazolam was the most frequently investigated sedative agent in the included reviews, with oral administration being the most common, although its effectiveness may depend on the patient’s cooperation during ingestion. However, the available evidence does not allow definitive conclusions regarding comparative effectiveness among agents. The intranasal route was frequently reported as a clinical alternative, possibly offering a faster onset and recovery times and, therefore, potentially more efficient management. Nitrous oxide was commonly used by dentists, and was consistently described as an option for pediatric patients with mild anxiety and moderate cooperation, supported by consistent evidence regarding its rapid induction and recovery profiles.

Sedation agents were generally reported as having a favorable safety profile, although incomplete reporting and heterogeneity limit definitive safety comparisons within the included studies, with adverse reactions tending to be limited and mostly mild and transient. Nevertheless, this safety is closely linked to the crucial importance of continuous monitoring of vital signs, which remains essential. The influence of the clinical setting (such as the dental office, outpatient clinic, and hospital), and the provider type (dentist or anesthesiologist) may influence the choice of agent and route of administration, potentially indicating the need for further guidelines that depend on the specific resources and skills of the operators.

In any case, pharmacological sedation should be closely aligned with the behavioral strategy to effectively manage and enhance the child’s residual cooperation during the dental procedure. However, the present study identified a notable gap in standardized reporting, particularly concerning the inconsistent reporting of data, including occasionally sedation and behavior scores, adverse events, recovery, dental procedure completion, and patient-reported outcomes, including acceptability and satisfaction expressed by children, caregivers, and providers, underscoring the need for further standardized studies to fill knowledge gaps and to allow structured quantitative comparisons and more-robust evidence synthesis.

## Figures and Tables

**Table 1:** Study population, intervention characteristics, and primary and secondary outcomes, grouped by midazolam administration route.

	PO	SL	BUCCAL	IV	IN	IM	PR
**Population**							
Sample size	1398	140	146	256	1026	60	751
Age (yo) Mean/Range	5.89 (*n* = 136/1398) Range: 1.3–16 (*n* = 1042/1398)	5.2 (*n* = 20/140) Range: 3–7 (*n* = 60/140)	MD Range: 2–15 (*n* = 91/146)	MD Range: 2–16 (*n* = 62/256)	4.64 (*n* = 119/1026) Range: 1.42–14 (*n* = 885/1026)	3.4 (*n* = 20/60) Range: 1–5 (*n* = 20/60)	3.61 (*n* = 120/751) Range: 1.5–10.5 (*n* = 676/751)
Gender ratio (M/F)	45 M/31 F (*n* = 86/1398)	MD	MD	MD	49 M/49 F (*n* = 98/1026)	11 M/9 F (*n* = 20/60)	59 M/61 F (*n* = 120/751)
Weight (kg) Mean/Range	19.38 (*n* = 135/1398)	MD	MD	MD	15.4 (*n* = 60) Range: 9–27 (*n* = 57/1026)	12.2 (*n* = 20/60)	17.80 (*n* = 25/751)
Comorbidities	None (*n* = 261/1398) Intellectual disability (*n* = 31/1398) Autism (*n* = 13/1398)	None (*n* = 30/140)	MD	None (*n* = 30/256)	None (*n* = 89/1026)	MD	None (*n* = 120/751)
**Intervention**							
Dosage Mean	0.76 mg/kg (*n* = 1337/1398)	0.23 mg/kg (*n* = 140/140)	0.27 mg/kg (*n* = 85/146)	0.076 mg/kg (*n* = 50/256) 0.5 mg/min (*n* = 236/256)	0.23 mg/kg (*n* = 970/1026)	0.2 mg/kg (*n* = 60/60)	0.43 mg/kg (*n* = 751/751)
Onset (min) Mean/Range	15.5 (*n* = 40/1398) Range: 15–35 (*n* = 111/1398)	MD	N/d	8 (*n* = 42/256)	13.19 (*n* = 210/1026) Range: 1.6–15 (*n* = 130/1026)	15.7 (*n* = 40/60)	16.88 (*n* = 136/751)
Duration (min) Mean/Range	55.2 (*n* = 11/1398) Range: 45–79 (*n* = 91/1398)	MD	MD	MD	MD	MD	180 (*n* = 24/751) Range: 45–79 (*n* = 45/751)
Sleep	N/d	N/d	MD	MD	No (*n* = 25/1026)	N/d	N/d
Recovery Time (min) Mean/Range	108.62 (*n* = 37/1398)	MD	MD	15.78 (*n* = 236/256)	37.51 (*n* = 57/1026) Range: <10 min (*n* = 10/1026)	MD	N/d
Provider		MD	MD			MD	
Anesthesiologist	38/1398			234/256	59/1026		
Dentist	20/1398				20/1026		45/751
Anesthesiologist and dentist	46/1398				31/1026		
Dental nurse	20/1398						
Dental assistant	16/1398						
Specialist trained in pediatric sedation and life support				42/256			
Setting (*n* of children)		MD	MD			MD	
Outpatient dental clinic	653/1398			30/256			631/751
Dental clinic/office	21/1398			10/256			
Dental clinic and operating room transfer if necessary					21/1026		
University hospital					10/1026		
Pedodontic clinic	16/1398						
Monitoring (*n* of children monitored)		MD					
Oxygen saturation	370/1398		36/146	194/256	142/1026		415/751
N/d respiratory rate	212/1398		36/146	10/256	132/1026	20/60	
N/d heart rate	107/1398		36/146	204/256	121/1026	20/60	75/751
ECG				194/256			
Blood pressure	167/1398		36/146	194/256	31/1026		25/751
N/d vital signs	31/1398			30/256			
Dental Procedure		N/d	N/d			N/d	
Restorative therapy	115/1398			19/256	108/1026		
Tooth extraction	139/1398			53/256	10/1026		25/751
**Primary outcome (s)**							
N. of reported successful sedations	130/1398	MD	20/146	MD	196/1026	MD	161/751
Sedation score Mean/Range		MD				MD	
Ramsay sedation scale	N/d			N/d			N/d
Modified Ramsay sedation scale					Moderate (*n* = 35/1026)		
Breitkopf and Buttner	3.5 (*n* = 54/1398)		3 (*n* = 36/146)	N/d			
Wilton’s sedation scale							4 (*n* = 1/751) “agitated”
5-point scale					“adequate” (*n* = 38/1026); “satisfactory” (*n* = 15/1026) Range: 4–5 (*n* = 21/1026)		
8-point scale	4.27 (*n* = 15/1398)						
10-point scale					4 (*n* = 20/1026)		
N/d degree of sedation scale	3.3 (*n* = 30/1398)						
Behavior Score Mean/Range							
Houpt scale	5.09 (*n* = 75/1398) Range: 5–6 (*n* = 8/1398), <5–6 (*n* = 3/1398)	5.6 (*n* = 40/140)	3.46 (*n* = 28/146) Range: 3–4 (*n* = 20/146)	5.8 (*n* = 10/256)	4.09 (*n* = 77/1026)	N/d	
Modified Houpt scale					5.29 (*n* = 38/1026)		
CFSS-DS	3.1 (*n* = 46/1398)		39.4 (*n* = 36/146)		“acceptable” (*n* = 29/1026)		
FLACC					3.83 (*n* = 56/1026)		
Vehnam’s clinical anxiety scale		0.45 (*n* = 20/140)			0.35 (*n* = 20/1026)		
OSUBRS	2.09 (*n* = 44/1398)						
Spielberg state anxiety inventory			39.4 (*n* = 36/146)				
Modified Frankl	3 (*n* = 13/1398)						
Global behavior rating scale					“excellent” (*n* = 86/1026); “adequate” (*n* = 18/1026); “satisfactory” (*n* = 33/1026)		
3-point scale	2.9 (*n* = 16/1398)						
Movement, crying, overall sedation, and behavior scale							2.78 (*n* = 93/751)
Adverse Events/Complications (*n* of Events)		MD					
None	186/1398			203/256	135/1026		14/751
Nausea	4/1398		6/146	14/256			
Vomiting	5/1398			14/256	1/1026		4/751
Headache	15/1398		6/146	14/256			1/751
Vertigo	10/1398						3/751
Speaking impairment	3/1398						7/751
Cough					6/1026	2/60	4/751
Sneezing					6/1026	2/60	
Hiccups					6/1026	2/60	3/751
Sore mouth				14/256			
Salivation	2/1398						
Sweating							2/751
Diplopia							3/751
Sleepiness/faint	13/1398		6/146	15/256			N/d
Confusion							7/751
Euphoria	2/1398						3/751
Disinhibitory reactions	46/1398						
Paradoxical reactions	4/1398						
Unusually quiet/lively (24 h post intervention)							N/d
Amnesia							N/d
Hallucination							21/751
Oxygen desaturation/ hypoxemia	50/1398				1/1026		3/751
Bradicardia							1/751
Yes but N/d	63/1398		20/146		23/1026	18/60	29/751
Management of Adverse Events/Complications	Oxygen application (*n* = 2/1398)	MD		MD	None, spontaneous recovery (*n* = 1/1026)	MD	MD
Procedure Completion	Yes (*n* = 284/1398) No (*n* = 18/1398)	MD	Yes (*n* = 42/146) No (*n* = 3/146)	Yes (*n* = 104/256) No (*n* = 90/256)	Yes (*n* = 215/1026) No (*n* = 5/1026)	MD	Yes (*n* = 209/751) Yes, with difficulties (*n* = 89/751) No (*n* = 1/751)
Reason for Interruption	Inability to tolerate agent (*n* = 5/1398) Paradoxical reactions (*n* = 1/1398)	MD	MD		MD	MD	MD
**Secondary outcome(s)**							
Acceptance (Scale: Score)							
Child	4-point scale: “excellent” (*n* = 18/1398), “good” (*n* = 6/1398), “moderate”(*n* = 1/1398), “poor” (*n* = 1/1398)	Al-Rakaf scale: “acceptance” (*n* = 8/140)	Self-reported: “No complaints in acceptance” (*n* = 21/146)	MD	Al-Rakaf scale: “acceptance” (*n* = 20/1026) Self-reported: “No complaints in acceptance” (*n* = 4/1026), “well accepted” (*n* = 21/1026)	MD	MD
Caregiver	MD	MD		MD		MD	MD
Provider	MD	MD		MD		MD	MD
Satisfaction (Scale: Score)							
Child	MD	MD	MD	MD	MD		MD
Caregiver	MD	MD	MD	10-point scale: 4.69 ± 0.7 (*n* = 194/256)	MD		MD
Provider	Self-reported: “very effective” (*n* = 31/1398)	MD	MD	3-point scale: 2.7 (*n* = 30/256)	Self-reported: “effective” (*n* = 31/1026)		MD

Abbreviations: Per os, “PO”; sublingual, “SL”; intravenous, “IV”; intranasal, “IN”; intramuscular, “IM”; per rectum, “PR”; missing data, “MD”; not-defined “N/d”; years old, “yo”; number, “*n*”; milligram, “mg”; minutes, “min”, males, “M”; females, “F”; kilogram, “kg”, electrocardiogram, “ECG”; Modified Observer Assessment of Alertness/Sedation scale, “MOAAS”; Face, Legs, Activity, Cry, Consolability scale, “FLACC”; Children’s Fear Survey Schedule-Dental Subscale, “CFSS-DS”; Ohio State University Behavior Rating Scale, “OSUBRS”.

**Table 2:** Study population, intervention characteristics, and primary and secondary outcomes, grouped by diazepam administration route.

	PO	PR
**Population**		
Sample size	44	90
Age (yo) Mean/Range	8.68 (*n* = 13/44) Range: 2–14.7 (*n* = 44/44)	2.67 (*n* = 45/90) Range: 1.5–3.5 (*n* = 90/90)
Gender Ratio (M/F)	MD	23 M/22 F (*n* = 45/90)
Comorbidities	Autism (*n* = 13/44)	MD
**Intervention**		
Dosage Mean	0.42 mg/kg (*n* = 33/44)	0.7 mg/kg (*n* = 90/90)
Provider	Anesthesiologist (*n* = 10/44)	Dentist (*n* = 45/90)
Setting (*n* of Children)	Dental clinic/office (*n* = 10/44)	Outpatient dental clinic (*n* = 45/90)
Monitoring (*n* of Children Monitored)	Oxygen saturation (*n* = 10/44) Respiratory rate (*n* = 10/44) Blood pressure (*n* = 10/44)	MD
Dental Procedure	Dental examination (*n* = 11/44)	N/d
**Primary outcome (s)**		
N. of Reported Successful Sedations	10/44	28/90
Sedation Score Mean/Range	MD	Self-reported: “agitated” (*n* = 13/90)
Behavior Score Mean/Range	Houpt scale: 4.5 (*n* = 10/44)	N/d

Abbreviations: Per os, “PO”; per rectum, “PR”; years old, “yo”; number, “*n*”; male, “M”; female, “F”; milligram, “mg”; kilogram, “kg”; missing data, “MD”; not-defined, “N/d”.

**Table 3:** Study population, intervention characteristics, and primary and secondary outcomes, grouped by zolpidem administration route.

	PO
**Population**	
Sample size	35
Age (yo) Mean/Range	MD/Range: 2–9 (*n* = 35/35)
Comorbidities	None (*n* = 15/35)
**Intervention**	
Dosage Mean	0.42 mg/kg (*n* = 35/35)
Provider	Anesthesiologist (*n* = 15/35)
**Primary outcome (s)**	
Sedation Score Mean/Range	8-point scale: 6.47 (*n* = 15/35)
Adverse Events/Complications	None (*n* = 20/35)

Abbreviations: per os, “PO”; years old, “yo”; number, “*n*”; milligram, “mg”; kilogram, “kg”; missing data, “MD”.

**Table 4:** Study population, intervention characteristics, and primary and secondary outcomes, grouped by triclofos administration route.

	PO
**Population**	
Sample Size	45
Age (yo) Mean/Range	MD/Range: 3–9 (*n* = 45/45)
Comorbidities	None (*n* = 45/45)
**Intervention**	
Dosage Mean	31.47 mg/kg (*n* = 45/45)
Provider	Anesthesiologist (*n* = 15/45)
Monitoring	Blood pressure (*n* = 30/45) Heart rate (*n* = 30/45) Respiratory rate (*n* = 30/45)
**Primary outcome (s)**	
Sedation Score Mean/Range	8-point scale: 5.00 (*n* = 15/45) N/d degree of sedation score: 2.73 (*n* = 30/45)
Adverse Events/Complications	None (*n* = 20/35)

Abbreviations: Per os, “PO”; years old, “yo”; number, “*n*”; milligram, “mg”; kilogram, “kg”; missing data, “MD”; not-defined, “N/d”.

**Table 5:** Study population, intervention characteristics, and primary and secondary outcomes, grouped by dexmedetomidine administration route.

	PO	SL	IV	IN
**Population**				
Sample size	134	42	30	410
Age (yo) Mean/Range	6.99 (*n* = 84/134) Range: 4–9 (*n* = 22/134)	Range: 5–7 (*n* = 42/42)	MD	7.03 ± 2.32 (*n* = 42/410) Range: 3–14 (*n* = 383/410)
Gender Ratio (M/F)	38 M/46 F (*n* = 84/134)	MD	MD	MD
Weight (kg) Mean/Range	13.53 (*n* = 84/134)	MD	MD	17.41 (*n* = 42/410)
Comorbidities	None (*n* = 84/134)	MD	None (*n* = 30/30)	None (*n* = 111/410)
**Intervention**				
Dosage Mean	4.10 µg/kg (*n* = 106/134)	1 µg/kg (*n* = 42/42)	1.7 µg/kg (*n* = 30/30)	1.19 µg/kg (*n* = 400/410)
Onset (min) Mean/Range	23.61 (*n* = 28/134)	MD	MD	14.52 (*n* = 57/410) Range: 7–25 (*n* = 22/410)
Sleep	N/d	MD	MD	Yes (*n* = 14/410)
Recovery time (min) Mean/Range	MD	MD	N/d	24.5 (*n* = 36)
Provider	N/d	MD		
Anesthesiologist			30/30	54/410
Anesthesiologist and dentist				42/410
Pediatric dentist				14/410
Setting (*n* of Children)	MD	MD		
Outpatient dental clinic			30/30	
Dental clinic and operating room transfer if necessary				36/410
Operating room				14/410
Monitoring (*n* of Children Monitored)		MD		
Oxygen saturation	112/134			110/410
Respiratory rate	112/134			42
Heart rate	28/134			68
Blood pressure	112/134			110/410
N/d vital signs			30/30	
Dental Procedure		N/d		
Restorative therapy			17/30	
Tooth extraction			13/30	42/410
N/d dental surgery	28/134			50/410
**Primary outcome (s)**				
N. of Reported Successful Sedations	23/134	MD	MD	133/410
Sedation Score Mean/Range		MD		
Ramsay sedation scale			N/d	
Modified AAPD scale	“satisfactory” (*n* = 23/134)			
MOAAS				3.61 (*n* = 14/410)
5-point scale				“satisfactory” (*n* = 39/410)
N/d degree of sedation scale				Score 4–5 (*n* = 35/410)
Behavior Score Mean/Range	N/d	N/d	MD	
Houpt scale				N/d
FLACC				3.75 (*n* = 42/410)
N/d behavior scale				“acceptable” (*n* = 22/410)
Adverse Events/Complications (*n* of Events)				
None	28/134	MD	30/30	145/410
Vomiting				1/410
**Secondary outcome (s)**				
Acceptance (Scale: Score)				
Child	MD	MD	MD	Self-reported: “fair to excellent acceptance” (*n* = 16/410); “well accepted” (*n* = 42/410)
Caregiver	MD	MD	MD	MD
Provider	MD	MD	MD	MD

Abbreviations: Per os, “PO”; intravenous, “IV”; intranasal, “IN”; missing data, “MD”; not-defined “N/d”; years old, “yo”; number, “*n*”; microgram, “µg”; minutes, “min”, males, “M”; females, “F”; kilogram, “kg”; Modified Observer Assessment of Alertness/Sedation scale, “MOAAS”; Face, Legs, Activity, Cry, Consolability scale, “FLACC”; American Academy Pediatric Dentistry, “AAPD”.

**Table 6:** Study population, intervention characteristics, and primary and secondary outcomes, grouped by chloral hydrate administration route.

	PO
**Population**	
Sample Size	149
Age (yo) Mean/Range	MD/Range: 0.1–7 (*n* = 129/149)
Comorbidities	None (*n* = 20/149)
**Intervention**	
Dosage Mean	60.69 mg/kg (*n* = 29/149) 0.8–1.0 mL/µg (*n* = 120) * verbatim from source study; non-standard unit; not harmonizable. Excluded from averages.
Provider	Nurse (*n* = 20/149)
Dental procedure	Dental examination (*n* = 129/149)
**Primary outcome (s)**	
N. of Reported Successful Sedations	96/149
Behavior Score Mean/Range	Houpt scale: 4.9 (*n* = 20/149)
Procedure Completion	Yes (*n* = 20/149)

Abbreviations: Per os, “PO”; years old, “yo”; number, “*n*”; milligram, “mg”; kilogram, “kg”; milliliters/microgram, “mL/µg”; missing data, “MD”. * verbatim from source study; non-standard unit; not harmonizable. Excluded from averages.

**Table 7:** Study population, intervention characteristics, and primary and secondary outcomes, grouped by promethazine administration route.

	PO
**Population**	
Sample Size	30
Age (yo) Mean/Range	MD/Range: 3–9 (*n* = 30/30)
Comorbidities	None (*n* = 30/30)
**Intervention**	
Dosage Mean	12.2 mg/kg (*n* = 30/30)
Monitoring	Blood pressure (*n* = 30/30) Heart rate (*n* = 30/30) Respiratory rate (*n* = 30/30)
**Primary outcome (s)**	
Sedation Score Mean/Range	8-point sedation scale: 2.73 (*n* = 30/30)

Abbreviations: Per os, “PO”; years old, “yo”; number, “*n*”; milligram, “mg”; kilogram, “kg”; missing data, “MD”.

**Table 8:** Study population, intervention characteristics, and primary and secondary outcomes, grouped by hydroxyzine administration route.

	PO
**Population**	
Sample Size	18
Age (yo) Mean/Range	3.9 (*n* = 18/18)/MD
Gender Ratio (M/F)	11 M/7 F (*n* = 18/18)
Weight (kg) Mean	18.1 (*n* = 18/18)
**Intervention**	
Dosage Mean	1.5 mg/kg (*n* = 18/18)
Provider	Anesthesiologist (*n* = 18/18)
Monitoring	Respiratory rate (*n* = 18/18) Heart rate (*n* = 18/18)
**Primary outcome (s)**	
Behavior Score Mean/Range	N/d, Ohio State Behavioral Rating scale (*n* = 18/18)

Abbreviations: Per os, “PO”; years old, “yo”; male, “M”; female, “F”; number, “*n*”; milligram, “mg”; kilogram, “kg”; not-defined, “N/d”.

**Table 9:** Study population, intervention characteristics, and primary and secondary outcomes, grouped by phenobarbital administration route.

	IM
**Population**	
Sample Size	112
Age (yo) Mean/Range	MD/Range: 0.1–6 (*n* = 112/112)
**Intervention**	
Dosage Mean	5 mg/kg (*n* = 112/112)
Dental procedure	Dental examination (*n* = 112/112)
**Primary outcome (s)**	
N. of Reported Successful Sedations	89/112

Abbreviations: Intramuscular, “IM”; years old, “yo”; number, “*n*”; milligram, “mg”; kilogram, “kg”; missing data, “MD”.

**Table 10:** Study population, intervention characteristics, and primary and secondary outcomes, grouped by tramadol administration route.

	PO
**Population**	
Sample Size	15
Age (yo) Mean/Range	MD/3–9 (*n* = 15/15)
Comorbidities	None (*n* = 15/15)
**Intervention**	
Dosage Mean	2 mg/kg (*n* = 15/15)
Provider	Anesthesiologist (*n* = 15/15)
Monitoring	Respiratory rate (*n* = 15/15) Heart rate (*n* = 15/15)
**Primary outcome (s)**	
Sedation Score Mean/Range	8-point sedation scale: 4.07 (*n* = 15/15)

Abbreviations: Per os, “PO”; years old, “yo”; number, “*n*”; milligram, “mg”; kilogram, “kg”; missing data, “MD”.

**Table 11:** Study population, intervention characteristics, and primary and secondary outcomes, grouped by meperidine administration route.

	IM
**Population**	
Sample Size	45
Age (yo) Mean/Range	3.35 (*n* = 45/45)/Range: 2–5 (*n* = 45/45)
**Intervention**	
Dosage Mean	1.08 mg/kg (*n* = 45/45)
Monitoring	Oxygen saturation (*n* = 45/45)
	Heart rate (*n* = 45/45)
	Blood pressure (*n* = 45/45)
Dental Procedure	Restorative therapy (*n* = 45/45)
**Primary outcome (s)**	
Sedation Score Mean/Range	Modified Houpt: N/d (*n* = 45/45)
Behavior Score Mean/Range	Dichotomous behavior scale: N/d (*n* = 45/45)
	10-point behavior scale: N/d (*n* = 45/45)
	Global rating scale: N/d (*n* = 45/45)
Adverse Events/Complications	Sleep/drowsiness (*n* = N/d/45)
Procedure Completion	Yes (*n* = 44/45) No (*n* = 1/45)
Reason for interruption	Unmanageable behavior (*n* = 1/45)

Abbreviations: Intramuscular, “IM”; years old, “yo”; number, “*n*”; milligram, “mg”; kilogram, “kg”; not-defined, “N/d”.

**Table 12:** Study population, intervention characteristics, and primary and secondary outcomes, grouped by sufentanil administration route.

	IN
**Population**	
Sample Size	10
Age (yo) Mean/Range	MD/Range: 1.5–6 (*n* = 10/10)
**Intervention**	
Dosage Mean	1.25 µg/kg (*n* = 10/10)
**Primary outcome (s)**	
Sedation Score Mean/Range	10-point sedation scale: 5.5 (*n* = 10/10)

Abbreviations: Intranasal, “IN”; years old, “yo”; number, “*n*”; microgram, “µg”; kilogram, “kg”; missing data, “MD”.

**Table 13:** Study population, intervention characteristics, and primary and secondary outcomes, grouped by ketamine administration route.

	PO	IN
**Population**		
Sample Size	106	253
Age (yo) Mean/Range	5.67 (*n* = 58/106) Range: 2–9 (*n* = 20/106)	4.93 (*n* = 134/253) Range: 1.42–14 (*n* = 253/253)
Gender Ratio (M/F)	44 M/42 F (*n* = 86/106)	MD
Weight (kg) Mean	18.89 (*n* = 28/106)	17.71 (*n* = 21/253)
Comorbidities	None (*n* = 58/106)	None (*n* = 21/253)
**Intervention**		
Dosage Mean	8.71 mg/kg (*n* = 106/106)	4.74 mg/kg (*n* = 166/253)
Onset (min) Mean/Range	21.11 (*n* = 28/106)	9.58 (*n* = 36/253) Range: 3.6–11.6 (*n* = 66/253)
Recovery Time (min) Mean/Range	MD	44.19 (*n* = 21/253) Range: <10 min (*n* = 7/253), 10–30 min (*n* = 3/253)
Provider	MD	Anesthesiologist and dentist (*n* = 31/253); anesthesiologist (*n* = 21/253)
Setting (*n* of Children)	MD	University hospital (*n* = 10/253)
Monitoring (*n* of Children Monitored)		
Oxygen saturation	28/106	62/253
Respiratory rate	28/106	42/253
Capnography		10/253
Heart rate		42/253
Blood pressure	28/106	42/253
Dental Procedure		
N/d oral surgery	28/106	
Tooth extraction	30/106	31/253
**Primary outcome (s)**		
N. of Reported Successful Sedations	MD	155/253
Sedation Score Mean/Range	N/d	
5-point scale		“adequate” (*n* = 106/253), “satisfactory” (*n* = 16/253) Range: 4–5 (*n* = 14/253)
10-point scale		4 (*n* = 20/253)
Behavior Score Mean/Range		
FLACC		3.5 (*n* = 21/253)
Movement, crying, overall sedation, and behavior scale	“good/better behavior” (*n* = 28/106)	
Adverse Events/Complications (*n* of Events)		
None		41/253
Vomiting	5/106	1/253
Paradoxical reactions	1/106	
Hallucination	5/106	
Oxygen desaturation/hypoxemia		3/253
N/d emergency reaction	2/106	
Management of Adverse Events/Complications	MD	None, spontaneous recovery (*n* = 3/253)

Abbreviations: Per os, “PO”; intranasal, “IN”; missing data, “MD”; not-defined, “N/d”; years old, “yo”; number, “*n*”; milligram, “mg”; kilogram, “kg”; minutes, “min”; males, “M”; females, “F”; Face, Legs, Activity, Cry, Consolability scale, “FLACC”.

**Table 14:** Study population, intervention characteristics, and primary and secondary outcomes, grouped by nitrous oxide administration route.

	INH
**Population**	
Sample Size	1547
Age (yo) Mean/Range	6.84 (*n* = 998/1547)/Range: 4–17 (*n* = 962/1547)
Gender Ratio (M/F)	274 M/290 F (*n* = 564/1547)
Weight (kg) Mean/Range	9.36 (*n* = 224/1547)
Comorbidities	None (*n* = 229/1547) Intellectual disability (*n* = 472/1547)
**Intervention**	
Dosage	30/70 (%nitrogen/oxygen) (*n* = 297/1547)
	40/60 (%nitrogen/oxygen) (*n* = 525/1547)
	Up to 40/60 (%nitrogen/oxygen) (*n* = 29/1547)
	50/50 (%nitrogen/oxygen) (*n* = 60/1547)
Onset (min) Mean/Range	6 (*n* = 42/1547) Range: 2–18 (*n* = 42/1547)
Duration (min) Mean/Range	37.21 (*n* = 193/1547) Range: 15–115 (*n* = 194/1547)
Recovery Time (min) Mean/Range	18.47 (*n* = 102/1547) Range: 2–18 (*n* = 42/1547)
Provider	Dentist (*n* = 694/1547) Anesthesiologist (*n* = 170/1547)
Setting (*n* of Children)	University hospital (*n* = 458/1547) Dental clinic/office (*n* = 61/1547)
Monitoring (*n* of Children Monitored)	N/d vital signs (*n* = 472/1547)
	Oxygen saturation (*n* = 447/1547)
	Heart rate (*n* = 447/1547)
	Respiratory rate (*n* = 262/1547)
	Blood pressure (*n* = 262/1547)
	Pretracheal auscultation (*n* = 170/1547)
	Visual assessment (*n* = 170/1547)
	N/d cardiovascular and respiratory parameters (*n* = 15/1547)
Dental Procedure	Tooth extraction (*n* = 705/1547)
	Restorative therapy (*n* = 542/1547)
	N/d oral surgery (*n* = 478/1547)
	Oral examination (*n* = 472/1547)
	Oral hygiene (*n* = 472/1547)
**Primary outcome(s)**	
N. of Reported Successful Sedations	816/1547
Sedation Score Mean/Range	Breitkopf and Buttner: 2.63 (*n* = 41/1547)
	Modified Ramsay: “moderate” (*n* = 35/1547)
	Ramsay sedation score: 1.7 (*n* = 15/1547)
	Range: Bispectral Index system: >90 (*n* = 60/1547)
Behavior Score Mean/Range	Houpt scale: 5.02 (*n* = 82/1547)
	CFSS-DS: 31.9 (*n* = 72/1547)
	VAS: 3.1 (*n* = 60/1547)
	Spielberg state anxiety inventory: 39.4 (*n* = 36/1547)
	Vehnam scale: 3.4 (*n* = 29/1547)
	N/d behavior scale: “adequate” (*n* = 19/1547), “excellent” (*n* = 14/1547)
	Range: Vehnam scale: 0–1 (*n* = 54/1547)
	Spielberg state anxiety inventory: 20–73 (*n* = 36/1547)
Adverse Events/Complications (*n* of Events)	
None	382/1547
Nausea	28/1547
Vomiting	14/1547
Headache	53/1547
Vertigo	9/1547
Eating/swallowing difficulties	30/1547
Epistaxis	1/1547
Hiccups	4/1547
Sore mouth	2/1547
Crying	1/1547
Sleepiness/faint	20/1547
Otalgia	2/1547
Procedure Completion	Yes (*n* = 633/1547) No (*n* = 171/1547)
Reason for Interruption	Dental procedure refusal (*n* = 2/1547) Lack of cooperation (*n* = 44/1547)
**Secondary outcome(s)**	
Satisfaction (Scale: Score)	
Child	Self-reported: “high” (*n* = 221/1547) 5-point scale: “satisfied” (*n* = 97%/N/d sample of respondents)
Caregiver	Self-reported: “high” (*n* = 221/1547) 5-point scale: “satisfied” (*n* = 79%/N/d sample of respondents)
Provider	MD

Abbreviations: Inhalation route, “INH”; nitrogen, “N_2_”; oxygen, “O_2_”; percentage, “%”; missing data, “MD”; not-defined, “N/d”; years old, “yo”; number, “*n*”; milligram, “mg”; minutes, “min”; males, “M”; females, “F”; kilogram, “kg”; Children’s Fear Survey Schedule-Dental Subscale, “CFSS-DS”; Visual Analog Scale, “VAS”.

**Table 15:** Proposed minimum reporting set for future research on pediatric dental sedation.

Domain	Essential Reporting Items	Rationale or Evidence Gap addressed
Patient Baseline	Age, weight (kg), American Society of Anesthesiologists status, and baseline anxiety/behavior (e.g., Frankl Scale)	Necessary to ensure comparability of study populations and the safety of drug dosages
Sedation Protocol Details	Specific agent, dosage (mg/kg), route of administration, onset, and recovery time	High heterogeneity in routes and dosages currently prevents robust meta-analysis
Provider Expertise	Qualification of the operator (e.g., anesthesiologist, a pediatric dentist, a trained nurse)	The safety profile and agent selection are strictly influenced by the provider’s training
Clinical Setting	Type of facility (e.g., private office, university hospital, operating room)	Infrastructure influences the management of potential emergencies and patient selection
Monitoring Standard	Continuous recording of oxygen rate, heart rate, respiratory rate, and blood pressure	Monitoring is often under-reported or inconsistently defined across systematic reviews. Standardized monitoring ensures an objective safety assessment
Clinical Outcomes	Use of validated scales for sedation (e.g., Ramsay) and clinical behavior (e.g., Houpt)	The use of non-standardized or “not-defined” scales is a major barrier to evidence synthesis.
Dental Procedure Completion	Report the completion of the planned dental treatment	Pharmacological success does not always translate into clinical success. This is often missing but critical for assessing the actual utility of a sedation protocol
Self-reported Outcomes	Acceptability and satisfaction scores from the child, caregiver, and dental provider	Identified as the most significant “knowledge gap” in the current literature, essential for patient-centered care

## Data Availability

The datasets generated and analyzed for the current umbrella review, including the data extraction matrix and AMSTAR-2 quality assessments, are available as Supplementary Materials associated with this article.

## Supplementary Materials

The following supporting information can be downloaded at https://www.mdpi.com/article/10.3390/children13030373/s1: File S1: Data extraction table; File S2: Data extracted and clustered by sedative agent and route of administration; File S3: Risk of bias.

## Appendix A

**Table A1:** Advanced search strategy.

Database	Search Date (up to)	Filters	Advanced Search Strategy
Pubmed/MEDLINE	26 March 2025	Article type “Systematic Review” and “Meta-analysis”	((“conscious sedation” [All Fields] OR (“inhalatory” [All Fields] AND (“procedural sedation” [MeSH Terms] OR (“procedural” [All Fields] AND “sedation” [All Fields]) OR “procedural sedation” [All Fields] OR (“conscious” [All Fields] AND “sedation” [All Fields]) OR “conscious sedation” [All Fields])) OR “intravenous conscious sedation” [All Fields] OR “enteral conscious sedation” [All Fields] OR “mild sedation” [All Fields] OR “nitrous oxide” [All Fields] OR “pharmacologic interventions” [All Fields] OR “n2O” [All Fields] OR (“benzodiazepin” [All Fields] OR “benzodiazepines” [Supplementary Concept] OR “benzodiazepines” [All Fields] OR “benzodiazepine” [All Fields] OR “benzodiazepines” [MeSH Terms] OR “benzodiazepinic” [All Fields] OR “benzodiazepins” [All Fields]) OR “Psychotropic Drugs” [All Fields] OR “intravenous sedation” [All Fields] OR “inhalatory sedation” [All Fields] OR “moderate sedation” [All Fields] OR “tranquilizing agents” [All Fields] OR (“sedate” [All Fields] OR “sedated” [All Fields] OR “sedating” [All Fields] OR “sedation” [All Fields] OR “sedations” [All Fields]) OR (“hypnosis” [MeSH Terms] OR “hypnosis” [All Fields]) OR (“ketamin” [All Fields] OR “ketamine” [Supplementary Concept] OR “ketamine” [All Fields] OR “esketamine” [Supplementary Concept] OR “esketamine” [All Fields] OR “esketamine” [Supplementary Concept] OR “esketamine” [All Fields] OR “ketamine” [MeSH Terms] OR “ketamine s” [All Fields] OR “ketamines” [All Fields]) OR (“midazolam” [Supplementary Concept] OR “midazolam” [All Fields] OR “midazolam” [MeSH Terms] OR “midazolam s” [All Fields] OR “midazolame” [All Fields])) AND (“dentistry” [MeSH Terms] OR “dentistry” [All Fields] OR “dentistry s” [All Fields] OR (“paedodontic” [All Fields] OR “paedodontics” [All Fields] OR “pediatric dentistry” [MeSH Terms] OR (“pediatric” [All Fields] AND “dentistry” [All Fields]) OR “pediatric dentistry” [All Fields] OR “pedodontics” [All Fields] OR “pedodontic” [All Fields]) OR “dental treatment” [All Fields] OR “dental treatments” [All Fields] OR “dental procedures” [All Fields] OR “dental management” [All Fields]) AND (“child” [MeSH Terms] OR “child” [All Fields] OR “children” [All Fields] OR “child s” [All Fields] OR “children s” [All Fields] OR “childrens” [All Fields] OR “childs” [All Fields] OR (“child” [MeSH Terms] OR “child” [All Fields] OR “children” [All Fields] OR “child s” [All Fields] OR “children s” [All Fields] OR “childrens” [All Fields] OR “childs” [All Fields]) OR (“adolescences” [All Fields] OR “adolescency” [All Fields] OR “adolescent” [MeSH Terms] OR “adolescent” [All Fields] OR “adolescence” [All Fields] OR “adolescents” [All Fields] OR “adolescent s” [All Fields]) OR (“young” [All Fields] OR “youngs” [All Fields]) OR (“paediatrics” [All Fields] OR “pediatrics” [MeSH Terms] OR “pediatrics” [All Fields] OR “paediatric” [All Fields] OR “pediatric” [All Fields]) OR (“adolescent” [MeSH Terms] OR “adolescent” [All Fields] OR “youth” [All Fields] OR “youths” [All Fields] OR “youth s” [All Fields]))) AND (meta-analysis [Filter] OR systematicreview [Filter])
Scopus	26 March 2025	Search within: Title, abstract, keywords; Document type “Review”	(TITLE-ABS-KEY ((“conscious sedation” OR “inhalatory conscious sedation” OR “intravenous conscious sedation” OR “enteral conscious sedation” OR “mild sedation” OR “nitrous oxide” OR “pharmacologic interventions” OR n2o OR benzodiazepine OR “Psychotropic Drugs” OR “intravenous sedation” OR “inhalatory sedation” OR “moderate sedation” OR “tranquilizing agents” OR sedation OR hypnosis OR ketamine OR midazolam)) AND TITLE-ABS-KEY ((dentistry OR pedodontics OR “dental treatment” OR “dental treatments” OR “dental procedures” OR “dental management”)) AND TITLE-ABS-KEY ((children OR child OR adolescent OR young OR pediatric OR youth))) AND (LIMIT-TO (DOCTYPE, “re”))
Web of Science	26 March 2025	Search within: all fields; Document type “Review article”	(“conscious sedation” OR “inhalatory conscious sedation” OR “intravenous conscious sedation” OR “enteral conscious sedation” OR “mild sedation” OR “nitrous oxide” OR “pharmacologic interventions” OR n2O OR benzodiazepine OR “Psychotropic Drugs” OR “intravenous sedation” OR “inhalatory sedation” OR “moderate sedation” OR “tranquilizing agents” OR sedation OR hypnosis OR ketamine OR midazolam) (All Fields) and (dentistry OR pedodontics OR “dental treatment” OR “dental treatments” OR “dental procedures” OR “dental management”) (All Fields) and (children OR child OR adolescent OR young OR pediatric OR youth) (All Fields) and Review Article (Document Types)
PROSPERO register	26 March 2025	Review status “Completed”	Search term: (“conscious sedation” OR “inhalatory conscious sedation” OR “intravenous conscious sedation” OR “enteral conscious sedation” OR “mild sedation” OR “nitrous oxide” OR “pharmacologic interventions” OR n2O OR benzodiazepine OR “Psychotropic Drugs” OR “intravenous sedation” OR “inhalatory sedation” OR “moderate sedation” OR “tranquilizing agents” OR sedation OR hypnosis OR ketamine OR midazolam) AND (dentistry OR pedodontics OR “dental treatment” OR “dental treatments” OR “dental procedures” OR “dental management”) AND (children OR child OR adolescent OR young OR pediatric OR youth)
The Cochrane Library	26 March 2025	Search within: Title, abstract, keywords	(“conscious sedation” OR “inhalatory conscious sedation” OR “intravenous conscious sedation” OR “enteral conscious sedation” OR “mild sedation” OR “nitrous oxide” OR “pharmacologic interventions” OR n2O OR benzodiazepine OR “Psychotropic Drugs” OR “intravenous sedation” OR “inhalatory sedation” OR “moderate sedation” OR “tranquilizing agents” OR sedation OR hypnosis OR ketamine OR midazolam) in Title Abstract Keyword AND (dentistry OR pedodontics OR “dental treatment” OR “dental treatments” OR “dental procedures” OR “dental management”) in Title Abstract Keyword AND (children OR child OR adolescent OR young OR pediatric OR youth) in Title Abstract Keyword—(Word variations have been searched)
