# Supplementary material for: Single-Agent Sedation for Behavioral Management in Pediatric Dentistry: An Umbrella Review of Agents, Routes of Administration, Providers, and Clinical Settings
Source: Children (Basel). 2026 Mar 6;13(3):373. doi: 10.3390/children13030373 (PMC13025869; doi:10.3390/children13030373)
Supplement: Supplementary file 1 [file children-13-00373-s001.zip › OK Supplementary File S2 - Clustered by Sedative Agent and Route of Administration.pdf]

Supplementary File S2 – Data Extracted from Included Systematic Reviews, Clustered by Sedative Agent and Route of Administration

Table S2a. Data extracted from the included systematic reviews for midazolam, clustered by route of administration.

	PO	SL	BUCCAL	IV	IN	IM	PR
**POPULATION**							
Sample size	1398	140	146	256	1026	60	751
Mean age/ age range (yo)	4.21 ± 1.65 (*n*=81) [16]<br>3.4 (*n*=11) [24]<br>10 (*n*=31) [31]<br>8.68 (*n*=13) [33]<br>Range:<br>1.3-9.3 (*n*=91) [21]<br>2-9 (*n*=614) [21,28,30]<br>2-10 (*n*=265) [16,18,20,22]<br>10-16 (*n*=72) [20]<br>MD	5.2 ± 1.15 (*n*=20) [16]<br>Range: 3-7 (*n*=60) [16,28]	MD<br>Range: 2-8 (*n*=55) [28]<br>10-15 (*n*=36) [20]	Range: 2-10 (*n*=20) [16,18]<br>12-16 (*n*=42) [20]<br>MD	4.64 ± 1.96 (*n*=119)[16]<br>MD<br>Range: 1.42-14 (*n*=794) [16,22,25,26,28,32]<br>4-8 (*n*=70) [20]<br>4-14 (*n*=21) [27]<br>MD	3.4 ± 0.6 yo (*n*=20) [16]<br>Range: 1-5 (*n*=20) [28]	4.33 ± 1.18 (*n*=75) [16]<br>2.42 (*n*=45) [16]<br>Range: 1.5-3.5 yo (*n*=45) [16]<br>2-9 (*n*=270) [21]<br>1.3-10.5 (*n*=361) [21]
Gender ratio (M/F)	45M/31F [16,31]<br>MD	MD	MD	MD	49M/49F<br>MD	11M/9F [16]	59M/61F [16]<br>MD
Weight (kg)	19.38 ± 8.15 (*n*=135) [16]<br>MD	MD	MD	MD	16.2 (*n*=20) [16]<br>17.5 ± 4.39 (*n*=20) [16]<br>12.6 ± 1.4 kg (*n*=20) [16]<br>9-27 (*n*=57) [25]<br>MD	12.2 ± 1.2 (*n*=20) [16]	17.80 ± 3.8 (*n*=25) [16]<br>MD
Comorbidities	None (*n*=261) [16,24,30]<br>Intellectual disability (*n*=31) [31]<br>Autism (*n*=13) [33]<br>MD	None (*n*=30) [16]	MD	None (*n*=30) [19]<br>MD	None (*n*=89)<br>MD	MD	None (*n*=120) [16]<br>MD
**INTERVENTION**							
Dosage	0.25 mg/kg (*n*=80) [16,32]<br>0.3 mg/kg (*n*=58) [20,31]	0.2 mg/kg (*n*=100) [16,28,32]<br>0.3 mg/kg (*n*=40) [28]	0.25 mg/kg (*n*=55) [20,28,32]<br>0.3 mg/kg (*n*=30)<br>N/d (*n*=25)<br>MD	0.5 mg/min (*n*=236) [16,20]<br>0.06 mg/kg (*n*=20)[16,18]	0.2 mg/kg (*n*=479) [16,25–28,32]<br>0.25 mg/kg (*n*=126) [28,32]	0.2 mg/kg (*n*=60) [16,28,32]	0.3 mg/kg (*n*=510) [16,21]<br>0.35 mg/kg (*n*=50) [16,21]<br>0.6 mg/kg (*n*=91) [21]<br>1 mg/kg (*n*=100) [16,21]

	0.5 mg/kg (*n*=881) [16,18,20–22,25,30–32]<br>0.7 mg/kg (*n*=151) [16,21,28,32]<br>0.75 mg/kg (*n*=30) [16]<br>1 mg/kg (*n*=101) [16,24,30]<br>7.5 mg (*n*=16) [16]<br>9.5 mg/kg (*n*=20) [16]			0.05 mg/kg (induction) + 0.06-0.012 mg/kg (maintenance) (*n*=30) [19]	0.3 mg/kg (*n*=252) [16,20,26,32]<br>0.4 mg/kg (*n*=23) [16,26]<br>0.45 mg/kg (*n*=10) [16]<br>0.5 mg/kg (*n*=13) [16]<br>1 μg/kg (*n*=67) [25,32]		
Onset (min)	15.5 ± 5 (*n*=20) [16]<br>15-33 (*n*=91) [21]<br>30-35 (*n*=20) [28]<br>15.5 (*n*=20)[32]<br>MD	MD	N/d (*n*=25)<br>MD	Mean: 8 (*n*=42) [20]<br>MD	15.10± 8.65 (*n*=155) [16,25,32]<br>3.6-1.6 (*n*=45) [26]<br>7.8 (*n*=55)[32]<br>5-15 (*n*=85) [32]<br>MD	15.7 ± 2.0 (*n*=20) [16]<br>15.7 (*n*=20) [32]	Mean: 15.33 (*n*=91) [21]<br>Mean: 20 (*n*=45) [21]<br>MD
Duration	45-79 (*n*=91) [21]<br>55.2 (*n*=11) [24]<br>MD	MD	MD	MD	MD	MD	Mean: 45-79 (*n*=45) [21]<br>Mean: 180 (*n*=24) [21]<br>MD
Sleep	MD<br>N/d (*n*=36) [16]	N/d (*n*=30) [16]<br>MD	MD	MD	No (*n*=25) [16]<br>MD	N/d (*n*=20) [16]	MD<br>N/d (*n*=120) [16]
Recovery time/score (min)	108.62 ± 58.7 (*n*=37) [25]<br>MD	MD	MD	Mean 8.02 ± 5.38 (*n*=194) [16]<br>Mean: 51.6 (*n*=42) [20]<br>N/d (*n*=30) [19]<br>MD	<10 min (*n*=10) [16]<br>37.51±17.91 (*n*=57) [25]<br>MD	MD	MD<br>N/d (*n*=50) [16]
Provider	Dentist (*n*=20) [16]<br>Dental nurse (*n*=20) [16]<br>Dental assistant (*n*=16) [16]	MD	MD	Anesthesiolo gist (*n*=234) [16,19]<br>Specialist trained in pediatric	Anesthesiologist (*n*=59) [16]<br>Dentist (*n*=20) [16]	MD	N/d research member (*n*=50) [16]<br>Dentist (*n*=45) [16]<br>MD

	Anesthesiologist and Dentist (*n*=46) [16,31]<br>Anesthesiologist (*n*=38) [16,30]<br>MD			sedation and life support (*n*=42) [20]	Anesthesiologist and Dentist (*n*=31) [16,25]<br>MD		
Setting	Pedodontic clinic (*n*=16) [16]<br>Dental office (*n*=10) [18]<br>Dental clinic (*n*=11) [24]<br>Outpatient dental clinic (*n*=653) [21,30]<br>MD	MD	MD	Dental office (*n*=10) [18]<br>Outpatient dental clinic (*n*=30) [19]<br>MD	University hospital (*n*=10) [16]<br>Dental clinic and operating room tranfer if necessary (*n*=21) [25]<br>MD	MD	Outpatient dental clinic (*n*=631) [21]<br>MD
Monitoring	Oxygen saturation, respiratory rate, blood pressure (*n*=100) [16]<br>Oxygen saturation, heart rate, respiratory rate (*n*=40) [16]<br>Oxygen saturation, blood pressure, heart rate (*n*=67) [16,20]<br>Oxygen saturation, respiratory rate (*n*=72) [20]<br>Oxygen saturation (*n*=91) [21]<br>N/d vital signs (*n*=31) [31]<br>MD	MD	Oxygen saturation, blood pressure, heart rate, respiratory rate (*n*=36) [20]	Oxygen saturation, heart rate, blood pressure, ECG (*n*=194) [16]<br>Respiratory rate, heart rate (*n*=10) [16]<br>N/d vital parameters (*n*=30) [19]<br>MD	Oxygen saturation, respiratory rate, blood pressure (*n*=21) [16]<br>Oxygen saturation, heart rate, respiratory rate (*n*=111) [16,20,25]<br>Oxygen saturation, heart rate, blood pressure (*n*=10) [16]<br>MD	Heart rate, respiratory rate (*n*=20) [16]	Oxygen saturation, blood pressure, heart rate (*n*=25) [16]<br>oxygen saturation, heart rate (*n*=50) [16]<br>Oxygen saturation (*n*=340) [21]<br>MD
Dental Procedure	N/d dental procedure (*n*=1144) [16,18,21,22,25,28,30–33]<br>Restorative dental therapy (*n*=115) [24]<br>Tooth extraction (*n*=139) [16,20]	N/d dental procedure (*n*=140) [16,28,32]	N/d dental procedure (*n*=146) [20,28,32]	N/d dental procedure (*n*=214) [16,18]<br>Tooth extraction (*n*=53) [19,20]	N/d dental procedure (*n*=898)[16,22,25–27,32]<br>Restorative dental therapy (*n*=108) [16,20]	N/d dental procedure (*n*=60) [16,28,32]	N/d dental procedure (*n*=726) [16,21]<br>Tooth extraction (*n*=25) [16]

				Restorative dental therapy (*n*=19) [19]	Tooth extraction (*n*=10) [16]		
**PRIMARY OUTCOME(S)**							
N. of reported successful sedations	130 [21,22,24,31]<br>MD	MD	20 [28]<br>MD	MD	196 [22,26–28,32]<br>MD	MD	161 [21,22]<br>MD
Sedation score, scales	N/d, Ramsay sedation scale (*n*=45) [16]<br>4.27 ± 0.46, 8-point sedation scaling rate (*n*=15) [16]<br>Score 2 (*n*=7); score 3 (*n*=13); score 4 (*n*=34), Breitkopf and Buttner [20]<br>N/d, Breitkopf and Buttner (*n*=14) [20]<br>N/d, Overall sedation scale (*n*=16) [16]<br>3.3 ± 0.7, degree of sedation scale (*n*=30) [16]<br>N/d, Wisconsin sedation scale (*n*=90) [16]<br>N/d, MOAAS (*n*=12)[22]<br>N/d, 4-point sedation scale (*n*=37) [25]<br>MD	MD	Score 3, Breitkopf and Buttner (*n*=36) [20]<br>MD	N/d, Ramsay sedation scale (*n*=30) [19]<br>N/d, Breitkopf and Buttner (*n*=42) [20]<br>MD	4 ± 1, 10-item sedation score (*n*=10) [16]<br>Score 4 (*n*=10), 10-item sedation score [26]<br>“adequate” (*n*=38), 5-item sedation scale [26]<br>“satisfactory” (*n*=15), 5-item sedation scale [26]<br>Score 4/5 (*n*=21), 5-item sedation score<br>N/d Ramsay sedation scale (*n*=15)<br>N/d, Houpt (*n*=16) [20]<br>Score “moderate”, Modified Ramsay sedation scale (*n*=35) [20]<br>N/d, Modified Houpt (*n*=20) [16]	MD	N/d, Ramsay sedation scale (*n*=75) [16]

					N/d, Modified AAPD sedation record (*n*=42) [16,25]<br>N/d, MOAAS (*n*=21)[22]<br>N/d, Ellis sedation score (*n*=70) [20,32]<br>N/d, 4-point sedation scale (*n*=21) [25]		
Behavior score, scales	4.90 ± 0.89, Houpt (*n*=50)<br>2.9 ± 0.3, 3-point scale (*n*=16) [16]<br>Score 6 (*n*=18), score 5 (*n*=3); score 4 (*n*=2); score 3 (*n*=2), Houpt [20]<br>N/d, Houpt (*n*=74) [20,24]<br>Mean 3.1, CFSS-DS (*n*=46) [20]<br>4.0±3.8 (*n*=16), OSUBRS<br>Score "restless/quiet"(*n*=28), OSUBRS<br>N/d, Modified Houpt (*n*=51) [16,32]<br>N/d, OSUBRS (*n*=16) [16]<br>Score 3 (*n*=13), Modified Frankl [31]<br>N/d, Modified Frankl (*n*=18) [31]<br>N/d, Breitkopf and Butner (*n*=20) [16]	N/d, Vehnam's clinical anxiety scale, cortisol level (*n*=20) [16,28]<br>N/d Vehnam's clinical anxiety scale (*n*=40)[28,32]<br>N/d, Modified Houpt (*n*=20) [32]<br>Score "fair" (*n*=4); score "good" (*n*=2); Score "excellent" (*n*=34), Houpt [28]<br>0.45 ± 1.10, Vehnam's	Mean 39.4, CFSS-DS (*n*=36) [20]<br>Mean 39.4, Spielberg state anxiety inventory (*n*=36) [20]<br>Score3-4 (*n*=20), Houpt [28]<br>Score "excellent" (*n*=3); "very good"(*n*=8); "fair" (*n*=5), "poor" (*n*=12), Houpt<br>N/d, Houpt (*n*=101) [20,28,32]	5.8 ± 0.4, Houpt (*n*=10) [16]<br>N/d, Houpt (*n*=42) [20]<br>MD	0.35 ± 0.59, Vehnam's clinical anxiety scale (*n*=20) [16]<br>2.2 ±0.6, Houpt (*n*=20) [16]<br>Score "excellent" (*n*=21); "very good" (*n*=13); "fair" (*n*=2); "poor (*n*=2), Modified Houpt [28]<br>Score "excellent" (*n*=86); "adequate" (*n*=18); N/d (*n*=16), Modified scale for behavior [28]<br>"satisfactory" (*n*=33); N/d (*n*=17), Global behavior rating scale	N/d Houpt (*n*=60) [16,28,32]	Score 4: "agitated", Wilton's sedation scale (*n*=1) [16]<br>N/d, Wilton's sedation scale (*n*=45) [16]<br>3.6 ± 0.6, Movement, crying, overall sedation, and behavior (*n*= 50) [16] MD<br>Score "oriented and calm", Movement, crying, overall sedation, and behavior (*n*= 36) [16]<br>Score "no movement", Movement, crying, overall sedation, and behavior (*n*= 7) [16]<br>N/d, Movement, crying, overall sedation, and behavior (*n*=7) [16] MD

	N/d, Frankl (n=20) [16]<br>N/d, Houpt (n=170) [16,32]<br>N/d, 4-point anxiolysis scale (n=37) [25]<br>Score 5/6 (n=8), Houpt [24]<br>< score 5/6 (n=3), Houpt [24]<br>N/d, Modified Houpt (n=40) [16,28]<br>MD	clinical anxiety scale (n=20) [16]			N/d, Fukuta scale (n=20) [16]<br>N/d, Vehnam's clinical anxiety scale, cortisol level (n=30) [16,32]<br>Mean: 2.77, FLACC (n=35) [20]<br>5.6 ± 1.1, FLACC (n=21),<br>N/d, FLACC scale (n=21) [16]<br>Score "acceptable" (n=29), CFSS-DS [32]<br>N/d, 4-point anxiolysis scale (n=36) [25]<br>N/d, 5-point behavior scale (n=21) [25]<br>N/d, Modified Houpt (n=40) [32]<br>N/d, Houpt (n=136) [32]<br>MD<br>4.12 ± 1.30, Houpt (n=38) [16]<br>Score "excellent" (n=19), Houpt [20]		
Adverse events/complications	None (n=186) [16,24,25,30,33]	MD	Sleepiness/headache/slight nausea (n=6) [20]	None (n=203) [16,19]	None (n=135) [16,25,27]	Sneezing /	None (n=14) [16]

	Dishinibitory reactions/hypoxiemia (*n*=46) [16]<br>vertigo/vomiting/speaking impairment (*n*=3) [16] nausea/vomiting (*n*=2) [16]<br>euphoria/hypoxyemia / headache (*n*=1) [16]<br>vertigo/nausea/salivation (*n*=2) [16]<br>Sleepiness/dizziness/ slight headache (*n*=5) [20]<br>Paradoxical reactions (*n*=4) [20,21]<br>Drowsiness/headache (*n*=9) [20]<br>Oxygen desaturation (*n*=2) [21]<br>Yes, N/d (*n*=21)[30,31]<br>N/d (*n*=42) [20]<br>MD		N/d (*n*=20) [20]<br>MD	Faint (*n*=1) [16]<br>Nausea, vomiting, drowsiness, headache, sore mouth (*n*=14)<br>MD	Oxygen desaturation (*n*=1)<br>Sneezing/coughing/ hiccups (*n*=6) [16]<br>Vomiting (*n*=1) [25]<br>N/d (*n*=23) [16]	coughing/<br>7iccup (*n*=2) [16]<br>N/d (*n*=18) [16]	speaking impairment/cough/confusion (*n*=4) [16]<br>hiccough/sweating/euphoria/speaking impairment/vomiting (*n*=3) [16]<br>diplopia/confusion/vertigo (*n*=3) [16]<br>bradycardia/vomiting/headache/hypoxiemia (*n*=1) [16]<br>hallucination (*n*=21) [16]<br>Amnesia/drawsiness (*n*=N/d) [21]<br>Oxygen desaturation (*n*=2) [21]<br>N/d (*n*=29) [16]<br>unusually quiet/lively (24 h post intervention) (*n*=N/d) [16]
Management of adverse events/complications	Oxygen application (*n*=2) [21]<br>MD	MD		MD	None, spontaneous recovery (*n*=1) [16]	MD	MD
Procedure completion	Yes (*n*=284) [16,20,28,30]<br>No (*n*=18) [16,20,30]<br>N/d (*n*=30) [16]<br>MD	MD	Yes (*n*=42) [20,28]<br>N/d (*n*=10) [20,28]<br>No (*n*=3) [20,28]<br>MD	Yes (*n*=104) [16]<br>No (*n*=90) [16]<br>MD	Yes (*n*=215) [16,20,28]<br>No (*n*=5) [16,28]<br>N/d (*n*=38) [28]<br>MD	MD	Yes (*n*=209) [16,21]<br>Yes, with difficulties (*n*=89) [21]<br>No (*n*= 1) [16]<br>N/d (*n*=45) [16]
Reason for interruption	Paradoxical reactions (*n*=1)<br>Inability to tolerate agent (*n*=5)<br>MD	MD	MD		MD	MD	MD
**SECONDARY OUTCOME(S)**							

Child/caregiver/provider acceptance: score, scale	4-point scale, “excellent” (*n*=18); “good” (*n*=6); “moderate”(*n*=1) poor” (*n*=1)/MD/MD [16]<br>MD	Al-Rakaf scale, acceptance (*n*=8)/MD/MD [16]<br>Al-Rakaf scale, N/d acceptance (*n*=12) [16]<br>N/d acceptance (*n*=20)/MD/MD [28]<br>MD	MD/MD/MD<br>“No complaints in acceptance”, self-reported (*n*=21)/MD/MD [32]	MD/MD/MD	acceptance, Al-Rakaf scale, (*n*=20)/MD/MD [16]<br>“No complaints in acceptance”, self-reported (*n*=4)/MD/MD [32]<br>“well accepted” (*n*=21), N/d scale/MD/MD [27]<br>MD/MD/MD	MD	MD
Child/caregiver/provider satisfaction	MD/MD/MD<br>MD/MD/ “very effective” (*n*=31), self-reported [32]	MD	MD/MD/MD	MD/4.69 ± 0.7 /MD, self-reported (1-10 rating scale) (*n*=194) [16]<br>MD/MD/ Score 2 (*n*=9), score 3 (*n*=21), 3-point scale self-reported [19]<br>MD/MD/MD	MD/MD/MD<br>MD/MD/ “effective” (*n*=31), self-reported [32]	MD	MD

Abbreviations: Abbreviations: *Per os*, “PO”; Sublingual, “SL”, Intravenous, “IV”; Intranasal, “IN”; Intramuscular, “IM”; *Per Rectum*, “PR”; Missing data, “MD”, Not applicable, “NA”; Not defined “N/d”; Years old, “yo”; Number, “n”; Milligram, “mg”; Minutes, “min”, Males/Females, “M/F”; Milligram per kilogram, “mg/kg”, Electrocardiogram, “ECG”; Modified Observer Assessment of Alertness/Sedation scale, “MOAAS”; Face, Legs, Activity, Cry, Consolability scale, “FLACC”; Children’s Fear Survey Schedule – Dental Subscale, “CFSS-DS”; Ohio State University Behavior Rating Scale, “OSUBRS”.

Table S2b. Data extracted from the included systematic reviews for diazepam, clustered by route of administration

	**PO**	**PR**
**POPULATION**		
Sample size	44	90

Mean age/<br>age range (yo)	8.68 (*n*=13)[33]<br>Range: 2-14.7 [16–18,33]	2.67 (*n*=45) [16]<br>Range:<br>1.5-3.5 (*n*=90) [16,21]
Gender ratio<br>(M/F)	MD	23M/22F [16]<br>MD
Weight<br>(kg)	MD	MD
Comorbidities	Autism (*n*=13)<br>MD	MD
**INTERVENTION**		
Dosage	0.3 mg/kg (*n*=13) [33]<br>0.5 mg/kg (*n*=20)[16,18]	0.7 mg/kg (*n*=90) [16,21]
Onset (min)	MD	MD
Duration	MD	MD
Sleep	MD	MD
Recovery time/score (min)	MD	MD
Provider	Anesthesiologist (*n*=10) [18]	Dentist (*n*=45) [16]<br>MD
Setting	Dental office (*n*=10) [18]<br>MD	Outpatient dental clinic (*n*=45) [21]<br>MD
Monitoring	Oxygen saturation, respiratory rate, blood pressure (*n*=10) [16]	MD
Dental Procedure	N/d dental procedure (*n*=33) [16,18,33]<br>Dental examination (*n*=11) [17]	N/d dental procedure (*n*=90) [16,21]
**PRIMARY OUTCOME(S)**		
N. of reported successful sedations	10 [33]	28 [16]
Sedation score, scales	MD	Score "agitated" (*n*=13); N/d score (*n*=32) Wilton's sedation scale [16]<br>MD
Behavior score, scales	4.5 ±0.5, Houpt (*n*=10) [16]	Wilton's sedation scale
Adverse events/complications	MD	MD

Management of adverse events/complications	MD	MD
Procedure completion	MD	MD
Reason for interruption	MD	MD
**SECONDARY OUTCOME(S)**		
Child/caregiver/provider acceptance: score, scale	MD/MD/MD	MD/MD/MD
Child/caregiver/provider satisfaction	MD/MD/MD	MD/MD/MD

Abbreviations: *Per os*, "PO"; *Per Rectum*, "PR"; Missing data, "MD", Not applicable, "NA"; Not defined "N/d"; Years old, "yo"; Number, "n"; Milligram, "mg"; Minutes, "min", Males/Females, "M/F"; Milligram per kilogram, "mg/kg".

Table S2c. Data extracted from the included systematic reviews for zolpidem, clustered by route of administration.

	**PO**
**POPULATION**	
Sample size	35
Mean age/<br>age range (yo)	MD<br>Range: 2-9 yo (*n*=35) [16]
Gender ratio<br>(M/F)	MD
Weight<br>(kg)	MD
Comorbidities	None (*n*=15) [16]<br>MD
**INTERVENTION**	
Dosage	0.4 mg/kg (*n*=35) [16]
Onset (min)	MD
Duration	MD
Sleep	MD
Recovery time/score (min)	MD
Provider	Anesthesiologist (*n*=15)<br>MD
Setting	MD
Monitoring	MD [16]

Dental Procedure	N/d dental procedure (*n*=15) [16]
**PRIMARY OUTCOME(S)**	
N. of reported successful sedations	MD
Sedation score, scales	6.47 ± 1.06, 8-point sedation scaling rate [16]<br>N/d, Sedation scoring system
Behavior score, scales	MD
Adverse events/complications	None (*n*= 20)<br>MD
Management of adverse events/complications	MD
Procedure completion	MD
Reason for interruption	MD
**SECONDARY OUTCOME(S)**	
Child/caregiver/provider acceptance: score, scale	MD/MD/MD
Child/caregiver/provider satisfaction	MD/MD/MD

Abbreviations: *Per os*, "PO"; Missing data, "MD", Not applicable, "NA"; Not defined "N/d"; Years old, "yo"; Number, "n"; Milligram, "mg"; Minutes, "min", Males/Females, "M/F"; Milligram per kilogram, "mg/kg".

Table S2d. Data extracted from the included systematic reviews for triclofos, clustered by route of administration.

	**PO**
**POPULATION**	
Sample size	45
Mean age/<br>age range (yo)	MD<br>Range:<br>3-9 (*n*=45)[16]
Gender ratio (M/F)	MD

Weight (kg)	MD
Comorbidities	None (*n*=45) [16]
**INTERVENTION**	
Dosage	12.2 mg/kg (*n*=30) [16]<br>70 mg/kg (*n*=15) [16]
Onset (min)	MD
Duration	MD
Sleep	MD
Recovery time/score (min)	MD
Provider	Anesthesiologist (*n*=15) [16]<br>MD
Setting	MD
Monitoring	Blood pressure, heart rate, respiratory rate (*n*=30) [16]
Dental Procedure	N/d dental procedure (*n*=45) [16]
**PRIMARY OUTCOME(S)**	
N. of reported successful sedations	MD
Sedation score, scales	Mean 5.00 ±0.85 (*n*=15), 8-point sedation scaling rate [16]<br>Mean 2.73 ± 0.5 (*n*=30), Degree of Sedation score [16]
Behavior score, scales	MD
Adverse events/complications	MD
Management of adverse events/complications	MD
Procedure completion	MD
Reason for interruption	MD
**SECONDARY OUTCOME(S)**	
Child/caregiver/provider acceptance: score, scale	MD/MD/MD
Child/caregiver/provider satisfaction	MD/MD/MD

Abbreviations: *Per os*, "PO"; Missing data, "MD", Not applicable, "NA"; Not defined "N/d"; Years old, "yo"; Number, "n"; Milligram, "mg"; Minutes, "min", Males/Females, "M/F"; Milligram per kilogram, "mg/kg".

Table S2e. Data extracted from the included systematic reviews for dexmedetomidine, clustered by route of administration.

	**PO**	**SL**	**IV**	**IN**
**POPULATION**				
Sample size	134	42	30	410
Mean age/<br>age range (yo)	6.99 ± 2.09 (*n*=84) [16]<br>MD<br>Range: 4-9 (*n*=22)	MD<br>Range: 5-7 (*n*=42) [32]	MD<br>MD	7.03 ± 2.32 (*n*=42) [16]<br>MD<br>Range: 3-14 (*n*=383) [16,22,25]

				MD
Gender ratio<br>(M/F)	38M/46F<br>MD	MD	MD	MD
Weight<br>(kg)	13.53 ± 8.39 (*n*=84) [16]<br>MD	MD	MD	17.41 ± 3.55 (*n*=42) [16]<br>MD
Comorbidities	None (*n*=84) [16]<br>MD	MD	None (*n*=30)[19]	None (*n*=111) [16,25]<br>MD
**INTERVENTION**				
Dosage	3 µg/kg (*n*=28) [16]<br>4 µg/kg (*n*=39) [16,27]<br>5 µg/kg (*n*=39) [16,27]	1 µg/kg (*n*=42) [32]	2 µg/kg (induction) + 0.4 µg/kg (mantainance) (*n*=30)[19]	1 µg/kg (*n*=283) [16,22,25]<br>1.5 µg/kg (*n*=95) [16,22]<br>2 µg/kg (*n*=11) [27]<br>2.5 µg/kg (*n*=11) [27]
Onset (min)	N/d (*n*=84) [16]<br>23.61 ± 4.12 (*n*=28) [29]	MD	MD	N/d (*n*=97) [16]<br>14.52 ± 4.9 (*n*=57) [25]<br>7-25 (*n*=22) [27]<br>MD
Duration	MD	MD	MD	MD
Sleep	N/d (*n*=84) [16]	MD	MD	Yes (*n*=14) [29]<br>MD
Recovery time/score (min)	MD	MD	N/d (*n*=30) [19]	N/d (*n*=54) [16]<br>24.5±5.1 (*n*=36) [25]<br>MD
Provider	N/d (*n*=84) [16]	MD	Anesthesiologist (*n*=30) [19]	Anesthesiologist (*n*=54) [16]<br>Dentist and anesthesiologist (*n*=42) [25]<br>Pediatric dentist (*n*=14) [29]<br>MD
Setting	MD	MD	Outpatient dental clinic (*n*=30) [19]	MD<br>Dental clinic/ operating room if necessary (*n*=36) [25]<br>Operating room (*n*=14) [29]
Monitoring	Oxygen saturation, respiratory rate, blood pressure (*n*=84) [16]<br>Oxygen saturation, respiratory rate, blood pressure, heart rate (*n*=28)[29]	MD	N/d vital parameters (*n*=30) [19]	Oxygen saturation, respiratory rate, blood pressure (*n*=42) [16]<br>Oxygen saturation, heart rate, blood pressure (*n*=68) [16]

Dental Procedure	N/d dental procedure (*n*=106) [16,27]<br>N/d Dental surgery (*n*=28) [29]	N/d dental procedure (*n*=42) [32]	Tooth extraction (*n*=13)<br>Restorative dental therapy (*n*=17) [19]	N/d dental procedure (*n*=318) [16,22]<br>N/d dental surgery (*n*=50) [25]<br>Tooth extraction (*n*=42) [25]
**PRIMARY OUTCOME(S)**				
N. of reported successful sedations	23	MD	MD	133 [22]<br>MD
Sedation score, scales	Score "satisfactory" (*n*=23), Modified AAPD [16]<br>N/d (*n*=61), Modified AAPD [16]<br>MD	MD	N/d, Ramsay (*n*=30)[19]	Score 4/5 (*n*=35); N/d score (*n*=7), 5-point sedation scale [27]<br>N/d, MOAAS (*n*=67) [16,22]<br>3.61 ± 1.71 (*n*=14), MOASS [29]<br>N/d AAPD (*n*=84) [16]<br>N/d, 4-point sedation score (*n*=36) [25]<br>Score "satisfactory" (*n*=39), 5-item sedation scale<br>N/d, Ramsay sedation scale (*n*=15)[32]<br>N/d Houpt (*n*=46) [32]<br>MD
Behavior score, scales	N/d, FLACC (*n*=84) [16]<br>MD	N/d, Venham scale for anxiety (*n*=42) [32]	MD	3.75 ± 0.84 (*n*=42), FLACC [27]<br>Score "acceptable behavior" (*n*=22); N/d (*n*=24), FLACCC [32]<br>N/d, Houpt (*n*=27) [16]<br>N/d FLACC (*n*=42) [16]<br>N/d 5-point behavior scale (*n*=42) [25]<br>N/d, Venham scale for anxiety (*n*=42) [32]<br>MD
Adverse events/complications	None (*n*=28) [16]<br>MD	MD	None (*n*=30) [19]	None (*n*=145) [16,25]<br>Vomiting (*n*=1) [25]<br>MD
Management of adverse events/complications	MD	MD	MD	MD
Procedure completion	MD	MD	MD	MD
Reason for interruption	MD	MD	MD	MD

SECONDARY OUTCOME(S)				
Child/caregiver/provider acceptance: score, scale	MD/MD/MD		MD/MD/MD	"fair to excellent acceptance" (*n*=16), N/d scale/MD/MD "well accepted" (*n*=42), N/d scale/MD/MD [27] MD/MD/MD
Child/caregiver/provider satisfaction	MD/MD/MD	MD/ MD/N/d, Modified Vernon Questionnaire (parental report) (*n*=42) /MD [32] MD/MD/MD	MD/MD/MD	MD/N/d, Modified Vernon Questionnaire (parental report) (*n*=42) (*n*=42)/MD [32] MD/MD/MD

Abbreviations: Abbreviations: *Per os*, "PO"; Sublingual, "SL", Intravenous, "IV"; Intranasal, "IN"; Intramuscular, "IM"; Missing data, "MD", Not applicable, "NA"; Not defined "N/d"; Years old, "yo"; Number, "n"; Milligram, "mg"; Minutes, "min", Males/Females, "M/F"; Milligram per kilogram, "mg/kg", Electrocardiogram, "ECG"; Modified Observer Assessment of Alertness/Sedation scale, "MOAAS"; Face, Legs, Activity, Cry, Consolability scale, "FLACC"; Children's Fear Survey Schedule – Dental Subscale, "CFSS-DS".

Table S2f. Data extracted from the included systematic reviews for chloral hydrate, clustered by route of administration.

	**PO**
**POPULATION**	
Sample size	149
Mean age/<br>age range (yo)	MD<br>Range: 0.1- 7 (*n*=129)
Gender ratio (M/F)	MD
Weight (kg)	MD
Comorbidities	MD<br>None (*n*=20) [16]
**INTERVENTION**	
Dosage	70 mg/kg (*n*=20) [16]<br>40 mg/kg (*n*=9) [17]<br>0.8-1.0 ml/µg (*n*=120) [17]
Onset (min)	MD
Duration	MD
Sleep	MD
Recovery time/score (min)	MD
Provider	Nurse (*n*=20) [16]
Setting	MD
Monitoring	MD

Dental Procedure	N/d dental procedure (*n*=20) [16]<br>Dental examination (*n*=129) [17]
**PRIMARY OUTCOME(S)**	
N. of reported successful sedations	96 [17]<br>MD
Sedation score, scales	MD
Behavior score, scales	4.9 ± 1.1 (*n*=20), Houpt [16]
Adverse events/complications	MD
Management of adverse events/complications	MD
Procedure completion	Yes (*n*=20) [16]<br>No (*n*=0) [16]
Reason for interruption	NA
**SECONDARY OUTCOME(S)**	
Child/caregiver/provider acceptance: score, scale	MD/MD/MD
Child/caregiver/provider satisfaction	MD/MD/MD

Abbreviations: Abbreviations: Abbreviations: *Per os*, "PO", Missing data, "MD", Not applicable, "NA"; Not defined "N/d"; Years old, "yo"; Number, "n"; Milligram, "mg"; milliliters, "ml"; Minutes, "min", Males/Females, "M/F"; Milligram per kilogram, "mg/kg", Electrocardiogram, "ECG"; Modified Observer Assessment of Alertness/Sedation scale, "MOAAS"; Face, Legs, Activity, Cry, Consolability scale, "FLACC"; Children's Fear Survey Schedule – Dental Subscale, "CFSS-DS"; Ohio State University Behavior Rating Scale, "OSUBRS".

Table S2g. Data extracted from the included systematic reviews for promethazine, clustered by route of administration.

	**PO**
**POPULATION**	
Sample size	30
Mean age/<br>age range (yo)	MD<br>Range:<br>3-9 yo (*n*=30) [16]
Gender ratio (M/F)	MD
Weight (kg)	MD
Comorbidities	None (*n*=30) [16]
**INTERVENTION**	
Dosage	12.2 mg/kg (*n*=30) [16]
Onset (min)	MD
Duration	MD
Sleep	MD
Recovery time/score (min)	MD
Provider	MD

Setting	MD
Monitoring	Blood pressure, heart rate, respiratory rate (*n*=30) [16]
Dental Procedure	N/d dental procedure (*n*=30) [16]
**PRIMARY OUTCOME(S)**	
N. of reported successful sedations	MD
Sedation score, scales	2.73 ±0.5, 8-point sedation scaling rate [16]
Behavior score, scales	MD
Adverse events/complications	MD
Management of adverse events/complications	MD
Procedure completion	MD
Reason for interruption	MD
**SECONDARY OUTCOME(S)**	
Child/caregiver/provider acceptance: score, scale	MD/MD/MD
Child/caregiver/provider satisfaction	MD/MD/MD

Abbreviations: Abbreviations: *Per os*, "PO", Missing data, "MD", Not applicable, "NA"; Not defined "N/d"; Years old, "yo"; Number, "n"; Milligram, "mg"; milliliters, "ml"; Minutes, "min", Males/Females, "M/F"; Milligram per kilogram, "mg/kg".

Table S2h. Data extracted from the included systematic reviews for hydroxizine, clustered by route of administration.

	**PO**
**POPULATION**	
Sample size	18
Mean age/age range (yo)	3.9 (*n*=18) [16]
Gender ratio (M/F)	11M/7F [16]
Weight (kg)	Mean: 18.1 kg (*n*=18) [16]
Comorbidities	MD
**INTERVENTION**	
Dosage	2 mg/kg (2h before) + 1mg/kg (20 min before) (*n*=18) [16]
Onset (min)	MD
Duration	MD
Sleep	MD
Recovery time/score (min)	MD
Provider	Anesthesiologist (*n*=18) [16]
Setting	MD
Monitoring	Respiratory rate, heart rate (*n*=18) [16]
Dental Procedure	N/d dental procedure (*n*=18) [16]
**PRIMARY OUTCOME(S)**	

N. of reported successful sedations	MD
Sedation score, scales	MD
Behavior score, scales	N/d, OSUBRS (*n*=18) [16]
Adverse events/complications	MD
Management of adverse events/complications	MD
Procedure completion	MD
Reason for interruption	MD
**SECONDARY OUTCOME(S)**	
Child/caregiver/provider acceptance: score, scale	MD/MD/MD
Child/caregiver/provider satisfaction	MD/MD/MD

Abbreviations: *Per os*, "PO"; Missing data, "MD", Not applicable, "NA"; Not defined "N/d"; Years old, "yo"; Number, "n"; Milligram, "mg"; Minutes, "min", Males/Females, "M/F"; Milligram per kilogram, "mg/kg", Ohio State Behavioral Rating scale "OSUBRS".

Table S2i. Data extracted from the included systematic reviews for phenobarbital, clustered by route of administration.

	**IM**
**POPULATION**	
Sample size	112 [17]
Mean age/age range (yo)	MD/0.1-6 (*n*=112) [17]
Gender ratio (M/F)	MD
Weight (kg)	MD
Comorbidities	MD
**INTERVENTION**	
Dosage	5 mg/kg (*n*=112) [17]
Onset (min)	MD
Duration	MD
Sleep	MD
Recovery time/score (min)	MD
Provider	MD
Setting	MD
Monitoring	MD
Dental Procedure	Dental examination (*n*=112) [17]
**PRIMARY OUTCOME(S)**	
N. of reported successful sedations	89 [17]
Sedation score, scales	MD
Behavior score, scales	MD

Adverse events/complications	MD
Management of adverse events/complications	MD
Procedure completion	MD
Reason for interruption	MD
**SECONDARY OUTCOME(S)**	
Child/caregiver/provider acceptance: score, scale	MD/MD/MD
Child/caregiver/provider satisfaction	MD/MD/MD

Abbreviations: Intramuscular "IM", Missing data, "MD", Not applicable, "NA"; Years old, "yo"; Number, "n"; Milligram, "mg"; milliliters, "ml"; Minutes, "min", Males/Females, "M/F"; Milligram per kilogram, "mg/kg.

Table S2j. Data extracted from the included systematic reviews for tramadol, clustered by route of administration.

	**PO**
**POPULATION**	
Sample size	15 [16]
Mean age/age range (yo)	MD/Range: 3-9 yo (*n*=15) [16]
Gender ratio (M/F)	MD
Weight (kg)	MD
Comorbidities	None (*n*=15) [16]
**INTERVENTION**	
Dosage	2 mg/kg (*n*=15) [16]
Onset (min)	MD
Duration	MD
Sleep	MD
Recovery time/score (min)	MD
Provider	Anesthesiologist (*n*=15) [16]
Setting	MD
Monitoring	MD
Dental Procedure	N/d dental procedure (*n*=15) [16]
**PRIMARY OUTCOME(S)**	
N. of reported successful sedations	MD
Sedation score, scales	4.07 ± 0.88 (*n*=15), 8-point sedation scaling rate [16]
Behavior score, scales	MD
Adverse events/complications	MD
Management of adverse events/complications	MD
Procedure completion	MD
Reason for interruption	MD

SECONDARY OUTCOME(S)	
Child/caregiver/provider acceptance: score, scale	MD/MD/MD
Child/caregiver/provider satisfaction	MD/MD/MD

Abbreviations: *Per os*, “PO”; Missing data, “MD”;Years old, “yo”; Number, “n”; Milligram, “mg”; Minutes, “min”, Males/Females, “M/F”; Milligram per kilogram, “mg/kg.

Table S2k. Data extracted from the included systematic reviews for meperidine, clustered by route of administration.

	**IM**
**POPULATION**	
Sample size	45
Mean age/<br>age range (yo)	3.35 ± 0.35 (*n*=45) [16]<br>Range: 2-5 (*n*=45) [16]
Gender ratio (M/F)	MD
Weight (kg)	MD
Comorbidities	MD
**INTERVENTION**	
Dosage	0.25 mg/kg (*n*=15) [16]<br>1.0 mg/kg (*n*=15) [16]<br>2.0 mg/kg (*n*=15) [16]
Onset (min)	MD
Duration	MD
Sleep	MD
Recovery time/score (min)	MD
Provider	MD
Setting	MD
Monitoring	Oxygen saturation, heart rate, pulse rate, blood pressure (*n*=45) [16]
Dental Procedure	Restorative dental therapy (*n*=45) [16]
**PRIMARY OUTCOME(S)**	
N. of reported successful sedations	MD
Sedation score, scales	N/d, Modified Houpt (*n*=45) [16]
Behavior score, scales	N/d, Dichomotous behavior scale (*n*=45) [16]<br>N/d, 10-point behavior scale (*n*=45) [16]<br>N/d, Global rating scale (*n*=45) [16]
Adverse events/complications	Sleep/ drowsiness (*n*=N/d) [16]
Management of adverse events/complications	MD
Procedure completion	Yes (*n*=44) [16]<br>No (*n*=1) [16]

Reason for interruption	Unmanageable behavior (*n*=1) [16]
**SECONDARY OUTCOME(S)**	
Child/caregiver/provider acceptance: score, scale	MD/MD/MD
Child/caregiver/provider satisfaction	MD/MD/MD

Abbreviations: Intramuscular "IM", Missing data, "MD", Not applicable, "NA"; Years old, "yo"; Number, "n"; Milligram, "mg"; milliliters, "ml"; Minutes, "min", Males/Females, "M/F"; Milligram per kilogram, "mg/kg.

Table S2l. Data extracted from the included systematic reviews for sulfentanil, clustered by route of administration.

	**IN**
**POPULATION**	
Sample size	10
Mean age/<br>age range (yo)	MD<br>1.5-6 (*n*=10)
Gender ratio (M/F)	MD
Weight (kg)	MD
Comorbidities	MD
**INTERVENTION**	
Dosage	1 µg/kg (*n*=5)<br>1.5 µg/kg (*n*=5)
Onset (min)	MD
Duration	MD
Sleep	MD
Recovery time/score (min)	MD
Provider	MD
Setting	MD
Monitoring	MD
Dental Procedure	N/d dental procedure (*n*=10)
**PRIMARY OUTCOME(S)**	
N. of reported successful sedations	MD
Sedation score, scales	Mean score 7 (*n*=5), mean score 4 (*n*=5), 10-item depth of sedation scale
Behavior score, scales	MD
Adverse events/complications	MD
Management of adverse events/complications	MD
Procedure completion	MD
Reason for interruption	MD
**SECONDARY OUTCOME(S)**	

Child/caregiver/provider acceptance: score, scale	MD/MD/MD
Child/caregiver/provider satisfaction	MD/MD/MD

Abbreviations: Intranasal, "IN"; Missing data, "MD"; Not defined, "N/d"; Years old, "yo"; Number, "n"; Milligram, "mg"; Minutes, "min"; Males/Females, "M/F"; Milligram per kilogram, "mg/kg"; Micron, "μm".

Table S2m. Data extracted from the included systematic reviews for ketamine, clustered by route of administration.

	**PO**	**IN**
**POPULATION**		
Sample size	106	253
Mean age/<br>age range (yo)	5.67±1.75 (*n*=58) [16]<br>MD<br>Range: 2-9 (*n*=20) [16]<br>MD	7.24±2.36 (*n*=21) [16]<br>4.5 (*n*=113) [26]<br>MD<br>Range: 1.42-14 (*n*=253) [16,22,25-27,32]
Gender ratio<br>(M/F)	44M/42F [16,29]<br>MD	MD
Weight<br>(kg)	18.89±4.33 (*n*=28) [16]<br>MD	17.71±5.36 (*n*=21) [16]<br>MD
Comorbidities	None (*n*=58) [16]<br>MD	None (*n*=21) [16]<br>MD
**INTERVENTION**		
Dosage	5 mg/kg (*n*=20) [16],<br>8 mg/kg^{-1} (*n*=56) [16,29]<br>12.5 mg/kg (*n*=30) [16]	0.3 mg/kg (*n*=15) [32]<br>3 mg/kg (*n*=20) [16,26]<br>5 mg/kg (*n*=42) [16,27]<br>6mg/kg (*n*=68) [26]<br>5 mg/kg (*n*=21) [22]
Onset (min)	21.11±4.10 (*n*=28) [29]<br>MD	11.57±2.18 (*n*=21) [25]<br>6.80 (*n*=15) [32]<br>Range 3.6-11.6 (*n*=66) [26]<br>MD
Duration	MD	MD
Sleep	MD	MD
Recovery time/score (min)	MD	< 10 min (*n*=7) [16]<br>10-30 min (*n*=3) [16]<br>44.19±5.24 (*n*=21) [25]<br>MD
Provider	N/d research member (*n*=30) [16]	Anesthesiologist (*n*=21) [16]

	MD	Anesthesiologist and Dentist (*n*=31) [16,25]<br>MD
Setting	MD	University hospital (*n*=10) [16]<br>MD
Monitoring	Oxygen saturation, blood pressure, respiratory rate (*n*=28) [29]<br>MD	Oxygen saturation, respiratory rate, heart rate, blood pressure (*n*=42) [16,25]<br>Oxygen saturation, capnography (*n*=10) [16]<br>Oxygen saturation (*n*=10) [26]
Dental Procedure	N/d dental procedure (*n*=48) [16]<br>N/d Dental surgery (*n*=28) [29]<br>Tooth extraction (*n*=30) [16]	N/d dental procedure (*n*=222) [16,22,26-27,32]<br>Tooth extraction (*n*=31) [16,25]
**PRIMARY OUTCOME(S)**		
N. of reported successful sedations	MD	155<br>MD
Sedation score, scales	N/d, 5-point sedation scoring system (*n*= 48) [16,29<br>N/d, Modified AAPD (*n*=28) [16]	4±1, 10-item sedation score (*n*=10) [16]<br>4, 10-item sedation score (*n*=10) [26]<br>"adequate" sedation, 5-item sedation scale (*n*=106) [26]<br>"satisfactory" sedation, 5-item sedation scale (*n*=16) [26]<br>Score 4/5 (*n*=14), 5-point scale<br>N/d, Modified AAPD (*n*=42) [16,25]<br>N/d, MOAAS (*n*=21 [22]<br>N/d,sedation depth assessment scale (*n*=15) [32]
Behavior score, scales	N/d FLACC (*n*= 28) [16]<br>N/d behavior score (*n*=28) [16]<br>"good/better behavior"(*n*=28), anxiety, movement, crying, overall behavior scale<br>N/d anxiety, movement, crying, overall behavior scale (*n*=2) [16]<br>MD	3.5 ±0.7, FLACC (*n*=21) [27]<br>N/d FLACC (*n*= 42) [16]<br>N/d, 5-point behavior response to treatment (*n*=21) [25]<br>N/d, standardized tool for behavioral response (*n*=68) [26]<br>N/d, behavioral response scale (*n*=15) [32]<br>MD
Adverse events/complications	Vomiting (*n*=5) [16]<br>Hallucination (*n*=5) [16]<br>Emergency reaction (*n*=2) [16]<br>Paradoxical reaction (*n*=1) [16]<br>MD	Oxygen desaturation (*n*=3) [16,26]<br>None (*n*=41) [25,27]<br>Vomiting (*n*=1) [25]<br>vomiting (*n*=N/d) [26]<br>MD
Management of adverse events/complications	NA	None, spontaneous recovery (*n*=3) [16,26]<br>MD

Procedure completion	N/d (*n*=28) [16]<br>MD	MD
Reason for interruption	MD	MD
**SECONDARY OUTCOME(S)**		
Child/caregiver/provider acceptance: score, scale	MD/MD/MD	MD/MD/MD
Child/caregiver/provider satisfaction	MD/MD/MD	MD/MD/MD

Abbreviations: *Per os*, "PO"; Intranasal, "IN"; Missing data, "MD"; Not applicable, "NA", Not defined, "N/d"; Years old, "yo"; Number, "n"; Milligram, "mg"; Minutes, "min"; Males/Females, "M/F"; Milligram per kilogram, "mg/kg"; Micron, "μm"; Modified Observer Assessment of Alertness/Sedation scale, "MOAAS"; American Academy of Pediatric Dentistry, "AAPD"; Face, Legs, Activity, Cry, Consolability scale, "FLACC".

Table S2n. Data extracted from the included systematic reviews for nitrous oxide, clustered by route of administration.

	**INH**
**POPULATION**	
Sample size	1547<br>N/d
Mean age/<br>age range (yo)	6.94±2.34 (*n*=465) [16,23]<br>6.74 (*n*=533) [23,31]<br>MD<br>Range: 4-17 (*n*=962) [20,23,28,31-32]
Gender ratio<br>(M/F)	274M/290F [16,23]<br>MD
Weight<br>(kg)	19.44 ±3.32 (*n*=30) [16]<br>7.8 (*n*=194) [23]<br>MD
Comorbidities	None (*n*=229) [16]<br>Intellectual disability (*n*=472) [31]<br>MD
**INTERVENTION**	
Dosage	30/70 (%N_2/O_2) (*n*=297) [20,32]<br>40/60 (%N_2/O_2) (*n*=525) [16,23]<br>Up to 40/60 (%N_2/O_2) (*n*=29) [16]<br>50/50 (%N_2/O_2) (*n*=60) [28]<br>N/d (%N_2/O_2) (*n*=696) [16,23,31-32]
Onset (min)	6 (*n*=42) [20]<br>Range: 2-18 (*n*=42) [20]<br>MD
Duration	44 (*n*=133) [23]

	Range: 15-115 (*n*=194) [23]<br>22.5±6.61 (*n*=60) [23]<br>MD
Sleep	MD<br>N/d (*n*=15) [16]
Recovery time/score (min)	23.3 (*n*=42) [20]<br>15.1±1.54 (*n*=60) [23]<br>Range: 2-18 (*n*=42) [20]<br>MD
Provider	Anesthesiologist (*n*=170) [16]<br>Anesthesiologist (*n*= N/d) [16]<br>Dentist (*n*=694) [16,23,31]<br>N/d research member (*n*=15) [16]<br>MD
Setting	University dental hospital (*n*=458) [23]<br>Dental clinic (*n*=61) [23]<br>MD
Monitoring	Oxygen saturation, heart rate, pretracheal auscultation, visual assessment (*n*=170) [16]<br>Oxygen saturation, heart rate, respiratory rate, blood pressure (*n*=262) [20]<br>N/d cardiovascular and respiratory parameters (*n*=15) [16]<br>Oxygen saturation, heart rate (*n*=15) [16]<br>N/d vital signs (*n*=472) [31]
Dental Procedure	N/d dental procedure (*n*=354) [16,28,32]<br>Tooth extraction (*n*=705) [20,23]<br>N/d oral surgery (*n*=6) [23]<br>Oral examination, oral hygiene, restorative dental treatment, oral surgery (*n*=472) [31]<br>Restorative dental therapy (*n*=70) [20]
**PRIMARY OUTCOME(S)**	
N. of reported successful sedations	816 [23,28,31]<br>MD
Sedation score, scales	Mean: 1.7 ± 0.6 (*n*=15), Ramsay sedation score [16]<br>>90, Bispectral Index system (*n*=60) [28]<br>N/d, Ramsay sedation score (*n*=30) [16,32]<br>N/d Ellis sedation score (*n*=70) [20,32]<br>Score 2 (*n*=24); score 3 (*n*=41), Breitkopf and Buttner [20]<br>N/d, Breitkopf and Buttner (*n*=75) [20]<br>“Moderate”, Modified Ramsay (*n*=35) [20]<br>MD

Behavior score, scales	N/d, Dichotomous behavior assessment (*n*=15) [16]<br>Mean: 3.4 ±0.92 (*n*=29), Vehnam scale (*n*=29) [16]<br>Score 0-1 (*n*=54), Vehnam scale [16]<br>N/d, Vehnam scale (*n*=82) [16]<br>N/d, Modified Vehnam scale (*n*=472) [31]<br>Mean: 31.9, CFSS-DS (*n*=72) [20]<br>Score 4 (*n*=1); score 4/5 (*n*=37); score 5 (*n*=23); score 6 (*n*=21), Houpt [20]<br>Mean:3.1±1.70 (*n*=60), VAS [23]<br>N/d, Houpt (*n*=149) [20,32]<br>Mean: 39.4, Spielberg state anxiety inventory (*n*=36) [20]<br>range 20-73, Spielberg state anxiety inventory (*n*=36) [20]<br>"excellent" (*n*=14); "adequate" (*n*=19), N/d modified scale for behavior/response to treatment [28]<br>N/d, N/d modified scale for behavior/response to treatment (*n*=27) [28]<br>N/d, Frankl (*n*=42) [20]<br>N/d FLACC (*n*=35) [32]<br>MD
Adverse events/complications	None (*n*=382) [16,23]<br>Nausea/headache/sleepiness (*n*=14) [20]<br>Amnesia (*n*=9) [20]<br>sleepiness/dizziness/headache (*n*=5) [20]<br>Vomiting (*n*=6) [20]<br>Nausea/vomiting/hiccough (*n*=4) [16]<br>Otalgia (*n*=2) [16]<br>Nausea (*n*=10) [23]<br>Headache (*n*=10) [23]<br>Soremouth (*n*=2) [23]<br>Crying (*n*=1) [23]<br>Dizziness (*n*=4) [23]<br>Faint (*n*=1) [23]<br>Epistaxis (*n*=1) [16]<br>Vomiting (24h post-intervention) (*n*=4) [23]<br>Headache (24 h post-intervention) (*n*=24) [23]<br>Eating/swallowing difficulties (24 hpost-intervention) (*n*=30) [23]<br>MD
Management of adverse events/complications	MD
Procedure completion	Yes (*n*=633) [16,20,23,28]; yes (*n*=N/d [16]); No (*n*=171) [16,20,23,28]
Reason for interruption	Dental procedure refusal (*n*=2) [23]<br>Cooperation lack (*n*=44) [23]

	MD
**SECONDARY OUTCOME(S)**	
Child/caregiver/provider acceptance: score, scale	MD/MD/MD
Child/caregiver/provider satisfaction	"high" (*n*=221), self-reported/ "high" (*n*=221), self-reported/MD [23]<br>"satisfied" (*n*=97%, N/d sample of responded), 5-point questionnaire/"satisfied" (*n*=79%, N/d sample of responded), 5-point questionnaire/MD [23]<br>MD/MD/MD

Abbreviations: Inhalation route, "INH"; Nitrogen, "N_2"; Oxygen, "O_2"; percentage, "%"; Missing data, "MD"; Not applicable, "NA", Not defined, "N/d"; Years old, "yo"; Number, "n"; Milligram, "mg"; Minutes, "min"; Males/Females, "M/F"; Milligram per kilogram, "mg/kg"; Modified Observer Assessment of Alertness/Sedation scale, Children's Fear Survey Schedule-Dental Subscale, "CFSS-DS"; "MOAAS"; American Academy of Pediatric Dentistry, "AAPD"; Face, Legs, Activity, Cry, Consolability scale, "FLACC"; Visual Analog Scale, "VAS".
