# Supplementary material for: Single-Agent Sedation for Behavioral Management in Pediatric Dentistry: An Umbrella Review of Agents, Routes of Administration, Providers, and Clinical Settings
Source: Children (Basel). 2026 Mar 6;13(3):373. doi: 10.3390/children13030373 (PMC13025869; doi:10.3390/children13030373)
Supplement: Supplementary file 1 [file children-13-00373-s001.zip › OK Supplementary File S3 - Risk of Bias.pdf]

Supplementary File S3 – Risk of Bias and Overall Quality

Table S3a. Evaluation of the risk of bias of included studies using the AMSTAR-2. First author, year, reference, sixteen AMSTAR-2 items to be evaluated, and quality of the study.

Studies	D1	D2	D3	D4	D5	D6	D7	D8	D9	D10	D11	D12	D13	D14	D15	D16	Quality
Ashley P.F., 2018 [16]	Y	Y	Y	Y	Y	Y	Y	Y	Y	Y	Y	Y	Y	Y	Y	Y	High
Chen Z., 2019 [17]	Y	PY	Y	Y	Y	Y	N	PY	Y	N	Y	Y	Y	Y	Y	Y	Low
da Silva B.L., 2024 [18]	Y	PY	Y	Y	Y	Y	PY	PY	PY	N	NV	NV	Y	Y	NV	N	Moderate
Goswami M., 2021 [19]	N	PY	N	N	N	N	PY	PY	PY	N	Y	N	N	Y	Y	Y	Critically low
Janiani P., 2023 [20]	N	PY	N	PY	Y	Y	Y	Y	Y	N	NV	NV	Y	Y	NV	Y	Moderate
Lam S.H.F., 2018 [21]	N	PY	Y	N	Y	N	PY	Y	Y	N	NV	NV	N	N	NV	N	Critically low
Lewis J., 2019 [22]	N	Y	Y	N	Y	N	PY	N	Y	N	NV	NV	N	N	NV	Y	Critically low
Lyratzopoulos G., 2003 [23]	Y	N	Y	Y	N	N	Y	PY	PY	N	NV	NV	Y	N	NV	Y	Low
Oliveira G.H.P., 2023 [24]	Y	Y	Y	Y	Y	N	PY	Y	Y	N	NV	NV	Y	Y	NV	Y	Moderate
Oza R.R., 2022 [25]	N	N	Y	Y	Y	Y	N	PY	Y	N	Y	Y	Y	Y	Y	Y	Critically low
Poonai N., 2017 [26]	N	N	Y	Y	Y	Y	PY	Y	Y	N	NV	NV	Y	Y	NV	Y	Low
Poonai N., 2019 [27]	N	N	Y	Y	Y	Y	PY	Y	Y	N	NV	NV	Y	Y	NV	Y	Low
Preethy N.A., 2021 [28]	Y	N	Y	Y	Y	Y	Y	Y	Y	N	NV	NV	Y	N	NV	Y	Low
Qiu, J., 2019 [29]	Y	N	Y	N	N	Y	PY	Y	Y	N	Y	Y	Y	Y	Y	N	Critically low
Rathi G.V., 2022 [30]	Y	Y	Y	PY	Y	Y	PY	Y	Y	N	NV	NV	Y	N	NV	Y	Moderate
Salerno C., 2023 [31]	Y	Y	Y	Y	Y	Y	PY	Y	Y	N	Y	Y	Y	Y	Y	Y	High

Swaminathan K., 2025 [32]	Y	Y	Y	PY	N	Y	PY	Y	Y	N	Y	Y	Y	Y	Y	Y	Moderate
Zupin L., 2024 [33]	Y	N	Y	PY	N	Y	N	Y	Y	N	Y	Y	Y	Y	Y	Y	Critically low

Abbreviations: Domains, “D”; Yes, "Y"; No, "N"; Partial Yes, "PY"; Not valuable, "NV"; underlined domains are the AMSTAR-2 critical domains.
