# Supplementary material for: Single-Agent Sedation for Behavioral Management in Pediatric Dentistry: An Umbrella Review of Agents, Routes of Administration, Providers, and Clinical Settings
Source: Children (Basel). 2026 Mar 6;13(3):373. doi: 10.3390/children13030373 (PMC13025869; doi:10.3390/children13030373)
Supplement: Supplementary file 1 [file children-13-00373-s001.zip › Supplementary File S1 Data Extracted from Included Systematic Reviews.pdf]

Supplementary File S1 – Data Extracted from Included Systematic Reviews

Table S1. Data extracted from the included systematic reviews.

Systematic Review	Study	Population	Intervention	Primary Outcomes	Secondary Outcomes
First Author<br>Year<br>Journal<br>Reference<br>Nr. Of included studies<br>Meta-analysis (yes/no)	Study design	Sample size<br>Mean age/age range<br>Gender ratio<br>Weight<br>comorbidities	Sedative agent<br>Route of administration<br>Dosage<br>Onset<br>Duration<br>Sleep<br>Recovery time/score<br>Provider<br>Setting<br>Monitoring<br>Dental procedure	Nr. of reported effective sedation<br><br>scales<br><br>Behavior score and scales<br><br>adverse events/complications<br><br>Management of adverse events/complications<br><br>Procedure completion<br>Reason for interruption	Acceptability (child, caregiver, provider)<br>Satisfaction (child, caregiver, provider)
Ashley<br>2018<br>Cochrane Library<br>[16]<br>31 RCT<br>Meta-analysis					
	RCT	30<br>Range from 1.42 and 5.17 yo<br>MD<br>MD<br>MD	Ketamine (*n*=10)<br>IN (*n*=10)<br>3 mg/kg (*n*=10)<br>Onset: MD<br>: MD<br>Duration: MD<br>Sleep: MD<br>Recovery time/score: <10 min (*n*=7); 10-30 min (*n*=3)<br>Provider: anesthesiologist/dentist (*n*=10)	Nr. of reported effective sedations: success rate (%)<br>sedation level (mean score): 4±1 (*n*=10);<br><br>scales 10- item sedation score; “acceptable” (score 4); “ideal” (score 5)<br><br>Behavior score and scales: MD<br><br>adverse events/complications: oxygen desaturation (*n*=1) ; N/d (*n*=9)	MD<br>MD<br>MD

			Monitoring: desaturation; blood pressure; capnography (if necessary)<br>N/d values<br>Setting: university hospital (*n*=10)<br>Dental procedure: teeth extraction (*n*=10)<br>Sedation type:	Management of adverse events/complications: none, spontaneous recovery (*n*=1)<br><br>Procedure completion: MD<br>Reason for interruption: N/A	
			Midazolam (*n*=10)<br>IN (*n*=10)<br>0.45 mg/kg (*n*=10)<br>Onset: MD<br>Duration: MD<br>Sleep: MD<br>Recovery time/score: <10 min (*n*=10)<br>Provider: anesthesiologist/dentist (*n*=10)<br>Monitoring: desaturation; blood pressure; capnography (if necessary)<br>N/d values<br>Setting: university hospital (*n*=10)<br>Dental procedure: teeth extraction (*n*=10)<br>Sedation type:	Nr. of reported effective sedations: success rate (%)<br>sedation level (mean score): 4±1 (*n*=10);<br><br>scales 10- item sedation score; “acceptable” (score 4); “ideal” (score 5)<br><br>Behavior score and scales: MD<br><br>adverse events/complications: none (*n*=10)<br><br>Management of adverse events/complications: N/A<br><br>Procedure completion: MD<br>Reason for interruption: N/A	
			Sufentanil (*n*=5)<br>IN (*n*=5)<br>1.5 μg/kg (*n*=5)<br>Onset: MD<br>Duration: MD<br>Sleep: MD<br>Recovery time/score: <10 min (*n*=1); 10-30 min (*n*=1); >30 min (*n*=3)	Nr. of reported effective sedations: yes (*n*=4); no (*n*=1)<br>sedation level (mean score): 7±3 (*n*=5);<br><br>scales 10- item sedation score; “acceptable” (score 4); “ideal” (score 5)<br><br>Behavior score and scales: MD	

			Provider: anesthesiologist/dentist (*n*=5)<br>Monitoring: desaturation; blood pressure; capnography (if necessary)<br>N/d values<br>Setting: university hospital (*n*=5)<br>Dental procedure: teeth extraction (*n*=5)<br>Sedation type:	adverse events/complications: oxygen desaturation (*n*=4); None (*n*=1)<br><br>Management of adverse events/complications: MD<br><br>Procedure completion: MD<br>Reason for interruption: N/A	
			Sufentanil (*n*=5)<br>IN (*n*=5)<br>1.0 μg/kg (*n*=5)<br>Onset: MD<br>Duration: MD<br>Sleep: MD<br>Recovery time/score: <10 min (*n*=4); 10-30 min (*n*=1);<br>Provider: anesthesiologist/dentist (*n*=5)<br>Monitoring: desaturation; blood pressure; capnography (if necessary)<br>N/d values<br>Setting: university hospital (*n*=5)<br>Dental procedure: teeth extraction (*n*=5)<br>Sedation type:	Nr. of reported effective sedations: yes (*n*=5); no (*n*=0)<br>sedation level (mean score): 4±1 (*n*=5);<br><br>scales 10- item sedation score; “acceptable” (score 4); “ideal” (score 5)<br><br>Behavior score and scales: MD<br><br>adverse events/complications: none (*n*=5)<br><br>Management of adverse events/complications: N/A<br><br>Procedure completion: MD<br>Reason for interruption: N/A	
	RCT	38<br>4.02 ±0.72 yo; range 3-5 yo<br>18M/20F<br>MD<br>None (*n*=38)	Midazolam (*n*=12)<br>IN (*n*=12)<br>0.3 mg/kg (*n*=12)<br>Onset (mean min): 10.25± 1.34 (*n*=12)<br>Duration: MD<br>Sleep: no (*n*=12)<br>Recovery time/score (mean min):31.45 ± 4.65 (*n*=12)	Nr. of reported effective sedations: MD<br><br>Sedation score, scale: mean 3.45 ±1.28 (*n*=12)<br><br>scales: Houpt sedation scale;<br><br>Behavior score and scales: MD	MD<br>MD<br>MD

			Provider: anesthesiologist (*n*=12)<br>Setting: university hospital (*n*=12)<br>Monitoring: blood pressure; heart rate; respiratory rate; oxygen saturation N/d levels(*n*=12)<br>Dental procedure: restorative dental therapy (*n*=12)<br>Sedation type:	adverse events/complications: none (*n*=12)<br><br>Management of adverse events/complications: N/A<br><br>Procedure completion: yes (*n*=9); no (*n*=3)<br>Reason for interruption: MD	
			Midazolam (*n*=13)<br>IN (*n*=13)<br>0.4 mg/kg (*n*=13)<br>Onset (mean min): 10.7 ±0.99 (*n*=13)<br>Duration: MD<br>Sleep: none (*n*=13)<br>Recovery time/score: 42.15± 2.54 (*n*=13)<br>Provider: anesthesiologist (*n*=13)<br>Setting: university hospital (*N*=13)<br>Monitoring: Monitoring: blood pressure; heart rate; respiratory rate; oxygen saturation N/d levels(*n*=13)<br>Dental procedure: restorative dental therapy (*n*=13)<br>Sedation type:	Nr. of reported effective sedations: MD<br>Sedation score:<br>mean 3.8 ±1.30 (*n*=13)<br><br>scales: Houpt sedation scale<br><br>Behavior score and scales: MD<br><br>adverse events/complications: none (*n*=13)<br><br>Management of adverse events/complications: N/A<br><br>Procedure completion: yes (*n*=12); no (*n*=1)<br>Reason for interruption: MD	
			Midazolam (*n*=13)<br>IN (*n*=13)<br>0.5 mg/kg (*n*=13)<br>Onset (mean min): 12.45± 2.20 (*n*=13)<br>Duration: 58.1± 3.46(*n*=13)	Nr. of reported effective sedations:<br>Sedation score: MD<br>mean 5.05 ±0.73 (*n*=13)<br><br>scales: Houpt sedation scale;	

			Sleep: MD<br>Recovery time/score: 106.8± 92.2 (*n*=20)<br>Provider: anesthesiologist (*n*=13)<br>Setting: university hospital (*N*=13)<br>Monitoring: Monitoring: blood pressure; heart rate; respiratory rate; oxygen saturation N/d levels(*n*=13)<br>Dental procedure: restorative dental therapy (*n*=13)<br>Sedation type:	Behavior score and scales: MD<br><br>adverse events/complications: none (*n*=13)<br><br>Management of adverse events/complications: N/A<br><br>Procedure completion: yes (*n*=13); no (*n*=0)<br>Reason for interruption: N/A	
	RCT	40<br>2.37 ± 0.28 (*n*=40)<br>27M/13F<br>MD<br>None (*n*=40);	ChlPO hydrate (*n*= 20)<br>PO (*n*=20)<br>70 mg/kg (*n*=20)<br>Onset (mean min): MD<br>Duration: MD<br>Sleep: N/d<br>Recovery time/score: MD<br>Provider: nurse (*n*=20)<br>Setting: MD<br>Monitoring: MD<br>Dental procedure: N/d dental procedure (*n*=20)<br>Sedation type:	Nr. of reported effective sedations: MD<br>Sedation score:<br>MD<br><br>scales: N/A<br>Behavior score and scales: Houpt; mean4.9 ± 1.1 (*n*=20)<br><br>adverse events/complications: MD<br><br>Management of adverse events/complications: N/A<br><br>Procedure completion: yes (*n*=20)<br>Reason for interruption: N/A	MD<br>MD<br>MD
			ChlPO hydrate + hydroxyzine (*n*= 20)<br>PO (*n*=20)<br>70 mg/kg + 2 mg/kg (*n*=20)<br>Onset (mean min): MD<br>Duration: MD<br>Sleep: N/d<br>Recovery time/score: MD<br>Provider: nurse (*n*=20)	Nr. of reported effective sedations: MD<br>Sedation score:<br>MD<br>scales: N/A<br><br>Behavior score and scales: Houpt 5± 0.7 (*n*=20)<br><br>adverse events/complications: MD	

			Setting: MD<br>Monitoring: MD<br>Dental procedure: N/d dental procedure (*n*=20)<br>Sedation type:	Management of adverse events/complications: N/A<br><br>Procedure completion: yes (*n*=20)<br>Reason for interruption: N/A	
	RCT	20<br>MD<br>MD<br>MD<br>None (*n*=20)	Midazolam (*n*=20)<br>0.5 mg/min (*n*=20)<br>IV (*n*=20)+ IN<br>Onset (mean min): MD<br>Duration: MD<br>Sleep: MD<br>Recovery time/score: mean min 6.5 ± 2.4 (*n*=20)<br>Provider: anesthesiologist (*n*=20)<br>Setting: MD<br>Monitoring: pulse oximeter, blood pressure, ECG (N/d values) (*n*=20)<br>Dental procedure: invasive N/d dental procedure (*n*=20)	Nr. of reported effective sedations: MD<br>Sedation score: MD<br>scales: N/A<br><br>Behavior score and scales: MD<br><br>adverse events/complications: MD<br><br>Management of adverse events/complications: N/A<br><br>Procedure completion: yes (*n*=10); no (*n*=10)<br>Reason for interruption: MD	MD<br>Caregiver satisfaction: (self-reported) 4.6 ± 0.7<br>1-10 rating scale<br>MD
	RCT	174<br>MD<br>MD<br>MD<br>None (*n*=174)	Midazolam (*n*=174)<br>0.5 mg/min (*n*=174)<br>IV (*n*=174)<br>Onset (mean min): MD<br>Duration: MD<br>Sleep: MD<br>Recovery time/score: mean min 8.2 ± 5.6 (*n*=174)<br>Provider: anesthesiologist (*n*=174)<br>Setting: MD<br>Monitoring: pulse oximeter, blood pressure, ECG (N/d values) (*n*=174)<br>Dental procedure: invasive N/d dental procedure (*n*=174)	Nr. of reported effective sedations: MD<br>Sedation score: MD<br>scales: N/A<br><br>Behavior score and scales: MD<br><br>adverse events/complications: faint (*n*=1); none (*n*=173)<br><br>Management of adverse events/complications: N/A<br><br>Procedure completion: yes (*n*=94); no (*n*=80)<br>Reason for interruption: MD	MD<br>Caregiver satisfaction: (self-reported) 4.7 ± 0.7 (10-item rating scale)<br>MD

	RCT	25<br>5.36 ±1.7 (*n*=25)<br>18M/7F<br>19.068 ± 3.43 kg (*n*=25)<br>None (*n*=25);<br>Uncooperative (Frankl's 1-2) (*n*=25)	Midazolam (*n*=25)<br>0.5 mg/kg (*n*=25)<br>PO (*n*=25)<br>Onset (mean min): MD<br>Duration: MD<br>Sleep: MD<br>Recovery time/score: MD<br>Provider: MD<br>Setting: MD<br>Monitoring: oxygen saturation, blood pressure, heart rate (N/d values) (*n*=25)<br>Dental procedure: tooth extraction (*n*=25)<br>Sedation type:	Nr. of reported effective sedations:<br>Sedation score: MD<br>scales: Ramsay sedation scale (N/d levels)<br><br>Behavior score and scales: MD<br><br>adverse events/complications: disinhibitory reactions/ hypoxemia (*n*=6); vertigo/vomiting/speaking impairment (*n*=3); nausea/vomiting (*n*=2); euphoria/hypoxemia/headache (*n*=1); vertigo/nausea/ salivation (*n*=2); none (*n*=11)<br><br>Management of adverse events/complications: MD<br><br>Procedure completion: yes (*n*=25); no (*n*=0)<br>Reason for interruption: N/A	Child acceptance of administration: 4-point scale from excellent to poor): Excellent (*n*=18); good (*n*=6); moderate (*n*=1); poor (*n*=0)
		25<br>4.40 ±1.51 (*n*=25)<br>12M/13F<br>17.804 ± 3.08 kg (*n*=25)<br>None (*n*=25)	Midazolam (*n*=25)<br>0.35 mg/min (*n*=25)<br>PR (*n*=25)<br>Onset (mean min): MD<br>Duration: MD<br>Sleep: MD<br>Recovery time/score: MD<br>Provider: MD<br>Setting: MD<br>Monitoring: oxygen saturation, blood pressure, heart rate (N/d values) (*n*=25)<br>Dental procedure: tooth extraction (*n*=25)<br>Sedation type:	Nr. of reported effective sedations:<br>Sedation score: MD<br>scales: Ramsay sedation scale (N/d levels)<br><br>Behavior score and scales: MD<br><br>adverse events/complications: disinhibitory reactions/ speaking impairment/ cough/confusion (*n*=4); hiccough/ sweating/euphoria/speaking impairment/vomiting (*n*=3); diplopia/confusion/ vertigo (*n*=3); bradycardia/ vomiting/ headache/ hypoxemia (*n*=1); none (*n*=14)<br><br>Management of adverse events/complications: MD	Child acceptance of administration: 4-point scale from excellent to poor): Excellent (*n*=5); good (*n*=5); moderate (*n*=13); poor (*n*=2)

				Procedure completion: yes (*n*=25); no (*n*=0)<br>Reason for interruption: N/A	
		15<br>5.33 ±0.99 (*n*=15)<br>6M/9F<br>20.01 ± 3.99 kg (*n*=15)<br>None (*n*=15)	N_2O(*n*=15)<br>40/60 (%O_2/N_2) (*n*=15)<br>PO + inhalation (*n*=15)<br>Onset (mean min): MD<br>Duration: MD<br>Sleep: MD<br>Recovery time/score: MD<br>Provider: MD<br>Setting: MD<br>Monitoring: MD<br>Dental procedure: N/d dental procedure (*n*=15)<br>Sedation type:	Nr. of reported effective sedations:<br>Sedation score:<br>scales: Ramsay sedation scale (*n*=15)<br>satisfactory level (*n*=1)<br>mild-level (*n*=9)<br>unsatisfactory level (*n*=5)<br>mean value: 1.7 ± 0.6 (*n*=15)<br><br>Behavior score and scales: MD<br><br>adverse events/complications: nausea/vomiting (*n*=4); hicchough (*n*=5); otalgia (*n*=2); epistaxis (*n*=1); none (*n*=3)<br>Management of adverse events/complications: MD<br><br>Procedure completion: yes (*n*=15); no (*n*=0)<br>Reason for interruption: N/A	MD<br>MD<br>MD
	RCT	15<br>3 to 9 yo<br>MD<br>MD<br>None (*n*=15);	Midazolam (*n*=15)<br>0.5 mg/kg (*n*=15)<br>PO (*n*=15)<br>Onset (mean min): MD<br>Duration: MD<br>Sleep: MD<br>Recovery time/score: MD<br>Provider: anesthesiologist + dentist<br>Setting: MD<br>Monitoring: MD<br>Dental procedure: N/d dental procedure (*n*=15)<br>Sedation type:	Nr. of reported effective sedations: MD<br>Sedation score: mean value: 4.27 ± 0.46 (*n*=15)<br><br>scales: 8-point Sedation scaling rate (*n*=15)<br><br>Behavior score and scales: MD<br><br>adverse events/complications: MD<br>Management of adverse events/complications: MD<br><br>Procedure completion: MD	MD<br>MD<br>MD

				Reason for interruption: N/A	
		60<br>3 to 9 yo<br>MD<br>MD<br>None (*n*=60);	Tramadol (*n*=15)<br>2mg/kg (*n*=15)<br>PO (*n*=15)<br>Onset (mean min): MD<br>Duration: MD<br>Sleep: MD<br>Recovery time/score: MD<br>Provider: anesthesiologist + dentist<br>Setting: MD<br>Monitoring: MD<br>Dental procedure: N/d dental procedure (*n*=15)<br>Sedation type:	Nr. of reported effective sedations: MD<br>Sedation score: mean value: 4.07 ± 0.88 (*n*=15)<br><br>scales: 8-point Sedation scaling rate (*n*=15)<br><br>Behavior score and scales: MD<br><br>adverse events/complications: MD<br>Management of adverse events/complications: MD<br><br>Procedure completion: MD<br>Reason for interruption: N/A	
		60<br>3 to 9 yo<br>MD<br>MD<br>None (*n*=60)	Triclofos (*n*=15)<br>70 mg/kg (*n*=15)<br>PO (*n*=15)<br>Onset (mean min): MD<br>Duration: MD<br>Sleep: MD<br>Recovery time/score: MD<br>Provider: anesthesiologist + dentist<br>Setting: MD<br>Monitoring: MD<br>Dental procedure: N/d dental procedure (*n*=15)<br>Sedation type:	Nr. of reported effective sedations: MD<br>Sedation score: mean value: 5.00 ± 0.85 (*n*=15)<br><br>scales: 8-point Sedation scaling rate (*n*=15)<br><br>Behavior score and scales: MD<br><br>adverse events/complications: MD<br>Management of adverse events/complications: MD<br><br>Procedure completion: MD<br>Reason for interruption: N/A	
		60<br>3 to 9 yo<br>MD	Zolpidem (*n*=15)<br>0.4 mg/kg (*n*=15)<br>PO (*n*=15)	Nr. of reported effective sedations: md<br>Sedation score: mean value: 6.47 ± 1.06 (*n*=15)	

		MD<br>None (n=60)	Onset (mean min): MD<br>Duration: MD<br>Sleep: MD<br>Recovery time/score: MD<br>Provider: anesthesiologist + dentist<br>Setting: MD<br>Monitoring: MD<br>Dental procedure: N/d dental procedure (n=15)<br>Sedation type:	scales: 8-point Sedation scaling rate (n=15)<br><br>Behavior score and scales: MD<br><br>adverse events/complications: MD<br>Management of adverse events/complications: MD<br><br>Procedure completion: MD<br>Reason for interruption: N/A	
	RCT	16<br>4-10 yo<br>MD<br>None (n=16)	Midazolam (n=16)<br>7.5 mg (n=16)<br>PO (n=16)<br>Onset (mean min): MD<br>Duration: MD<br>Sleep: MD<br>Recovery time/score: MD<br>Provider: dental assistant (n=16)<br>Setting: pedodontic clinic (n=16)<br>Monitoring: MD<br>Dental procedure: N/d dental procedure (n=16)<br>Sedation type	Nr. of reported effective sedations: MD<br>Sedation score: N/d<br><br>scales: Overall sedation score<br><br>Behavior score and scales: 3- point scale (n=16)<br>mean value 2.9 ± 0.3 (n=16)<br>adverse events/complications: MD<br>Management of adverse events/complications: N/A<br><br>Procedure completion: MD<br>Reason for interruption: N/A	MD<br>MD<br>MD
	RCT	15<br>5.01 ± 1.03 yo (n=15)<br>9M/6F<br>19.73 ± 4.77 kg<br>MD	N_2O (n=15)<br>60/40 (%O_2/N_2)<br>Inhalation + PO (n=15)<br>Onset (mean min): MD<br>Duration: MD<br>Sleep: MD<br>Recovery time/score: MD<br>Provider: MD<br>Setting: MD<br>Monitoring: pulse oximeter	Nr. of reported effective sedations: MD<br>Sedation score: mean score 1.3 ± 0.6 (n=15)<br><br>scales: Ramsay sedation score (n=15)<br><br>Behavior score and scales: MD<br><br>Adverse events/complications: MD	MD<br>MD<br>MD

			Dental procedure: N/d dental procedure (*n*=15)<br>Sedation type	Management of adverse events/complications: MD<br><br>Procedure completion: MD<br>Reason for interruption: N/A	
	RCT	45<br>2.67 yo; range 1.5-3.5 yo<br>23M/22F<br>MD<br>MD	Diazepam (*n*=45)<br>0.7 mg/kg (*n*=45)<br>PR (*n*=45)<br>Onset (mean min): MD<br>Duration: MD<br>Sleep: MD<br>Recovery time/score: MD<br>Provider: Dentist (*n*=45)<br>Setting: MD<br>Monitoring: MD<br>Dental procedure: N/d dental procedure (*n*=45)<br>Sedation type	Nr. of reported effective sedations:28<br>Sedation score:<br>Good/better sedation (*n*=28)<br><br>scales: Wilton's sedation scale (4-point scale from drowsy to agitated)<br><br>Behavior score and scales: Wilton's sedation scale (4-point scale from drowsy to agitated) "agitated" (*n*=1)<br><br>Adverse events/complications: Lasting effects of aggressiveness/tiredness/uncoordinated movements (*n*=N/d)<br>Management of adverse events/complications: N/A<br><br>Procedure completion: N/d<br>Reason for interruption: N/A	MD<br>MD<br>MD
		45<br>2.42 yo; range 1.5-3.5 yo<br>23M/22F<br>MD<br>MD	Midazolam (*n*=45)<br>0.3 mg/kg (*n*=45)<br>PR (*n*=45)<br>Onset (mean min): MD<br>Duration: MD<br>Sleep: MD<br>Recovery time/score: MD<br>Provider: Dentist (*n*=45)<br>Setting: MD<br>Monitoring: MD<br>Dental procedure: N/d dental procedure (*n*=45)<br>Sedation type	Nr. of reported effective sedations: 43<br>Sedation score:<br>Good/better setation (*n*=28)<br><br>scales: Wilton's sedation scale (4-point scale from drowsy to agitated)<br><br>Behavior score and scales: Wilton's sedation scale (4-point scale from drowsy to agitated<br>"agitated" (*n*=1)	

				Adverse events/complications: unusually quiet/lively (24h post treatment)<br>Management of adverse events/complications: N/A<br><br>Procedure completion: N/d<br>Reason for interruption: N/A	
	RCT	20 per group<br>2-9 yo<br>MD<br>MD<br>MD	Midazolam (*n*=20)<br>PO (*n*=20)<br>9.5 mg/kg (*n*=20)<br>Onset (mean min): MD<br>Duration: MD<br>Sleep: MD<br>Recovery time/score: MD<br>Provider: MD<br>Setting: MD<br>Monitoring: MD<br>Dental procedure: N/d<br>dental procedure (*n*=20)<br>Sedation type	Nr. of reported effective sedations: MD<br>Sedation score:<br>Mean score: N/d<br><br>scales: Sedation scoring system (5-point scale from asleep to awake)<br><br>Behavior score and scales: MD<br><br>Adverse events/complications: None (*n*=20)<br>Management of adverse events/complications: N/A<br><br>Procedure completion: MD<br>Reason for interruption: MD	MD<br>MD<br>MD
			Ketamine (*n*=20)<br>PO (*n*=20)<br>5 mg/kg (*n*=20)<br>Onset (mean min): N/d<br>Duration: MD<br>Sleep: MD<br>Recovery time/score: MD<br>Provider: MD<br>Setting: MD<br>Monitoring: MD<br>Dental procedure: N/d<br>dental procedure (*n*=20)<br>Sedation type	Nr. of reported effective sedations: MD<br>Sedation score:<br>Mean score: N/d<br><br>scales: Sedation scoring system (5-point scale from asleep to awake)<br><br>Behavior score and scales: MD<br><br>Adverse events/complications: None (*n*=20)<br>Management of adverse events/complications: N/A<br><br>Procedure completion: MD	

				Reason for interruption: MD	
			Zolpidem (*n*=20)<br>PO (*n*=20)<br>0.4 mg/kg (*n*=20)<br>Onset (mean min): N/d<br>Duration: MD<br>Sleep: MD<br>Recovery time/score: MD<br>Provider: MD<br>Setting: MD<br>Monitoring: MD<br>Dental procedure: N/d<br>dental procedure (*n*=20)<br>Sedation type	Nr. of reported effective sedations: MD<br>Sedation score:<br>Mean score: N/d<br><br>scales: Sedation scoring system (5-point scale from asleep to awake)<br><br>Behavior score and scales: MD<br><br>Adverse events/complications: None (*n*=20)<br>Management of adverse events/complications: N/A<br><br>Procedure completion: MD<br>Reason for interruption: MD	
	RCT	170<br>6.2 ± 1.9 yo<br>MD<br>MD<br>None,(*n*=170)	N_2O (*n*=170)<br>Ihnalation (*n*=170)<br>60/40 (%O_2/N_2)<br>Onset (mean min): MD<br>Duration: MD<br>Sleep: MD<br>Recovery time/score: MD<br>Provider: anaesthesiologist (*n*=170)<br>Setting: MD<br>Monitoring: pulse oximeter, capnograph, pretracheal stethoscope, visual assessment, auscultation and visualization of chest movements<br>Dental procedure: N/d<br>dental procedure (*n*=170)<br>Sedation type	Nr. of reported effective sedations: MD<br>Sedation score:<br>effective sedation (*n*=89)<br>"relaxed" (*n*=114);<br>"Failed" (*n*=19)<br><br>Mean score: MD<br><br>scales: Venham scale, level of sedation and failure rate<br><br>Behavior score and scales:<br><br>Adverse events/complications: None (*n*=170)<br>Management of adverse events/complications: N/A<br><br>Procedure completion: yes (*n*=88); no (82)<br>Reason for interruption: MD	MD<br>MD<br>MD

	RCT	20<br>3.40 ± 0.92 yo (*n*=20)<br>11M/9F<br>17 ± 3.6 kg (*n*=20)<br>None, (*n*=20); ASA I, uncooperative (Frankl's score slightly/definitely negative)	Midazolam (*n*=20)<br>PO (*n*=20)<br>0.7 mg/kg (*n*=20)<br>Onset (mean min): 15.5 ± 5 (*n*=20)<br>Duration: MD<br>Sleep: MD<br>Recovery time/score: MD<br>Provider: dentist (*n*=20)<br>Setting: MD<br>Monitoring: oxygen saturation, heart rate, respiratory rate (*n*=20)<br>Dental procedure: N/d dental procedure (*n*=20)<br>Sedation type	Nr. of reported effective sedations: MD<br>Sedation score:<br>N/d<br><br>scales: Modified Houpt<br><br>Behavior score and scales: Modified Houpt<br><br>Adverse events/complications: None (*n*=20)<br>Management of adverse events/complications: N/A<br><br>Procedure completion: yes (*n*=20); no (*n*=0)<br>Reason for interruption: N/A	MD<br>MD<br>MD
		20<br>3.21 ± 0.82 yo (*n*=20)<br>11M/9F<br>16.2 kg (*n*=20)<br>None, (*n*=20); ASA I, uncooperative (Frankl's score slightly/definitely negative)	Midazolam (*n*=20)<br>IN (*n*=20)<br>0.3 mg/kg (*n*=20)<br>Onset (mean min): 5.55 ± 2.2 (*n*=20)<br>Duration: MD<br>Sleep: MD<br>Recovery time/score: MD<br>Provider: dentist (*n*=20)<br>Setting: MD<br>Monitoring: oxygen saturation, heart rate, respiratory rate (*n*=20)<br>Dental procedure: N/d dental procedure (*n*=20)<br>Sedation type	Nr. of reported effective sedations: MD<br>Sedation score:<br>N/d<br><br>scales: Modified Houpt<br><br>Behavior score and scales: Modified Houpt<br><br>Adverse events/complications: None (*n*=20)<br>Management of adverse events/complications: N/A<br><br>Procedure completion: yes (*n*=20); no (*n*=0)<br>Reason for interruption: N/A	
	RCT	12<br>MD<br>MD	Dexmedetomidine (*n*=12)<br>IN (*n*=12)<br>1 μg/kg + 30 mL (*n*=12)	Nr. of reported effective sedations: MD<br>Sedation score:<br>N/d	MD<br>MD<br>MD

		MD<br>None (*n*=12)	Onset (mean min): MD<br>Duration: MD<br>Sleep: MD<br>Recovery time/score: MD<br>Provider: anesthetist (*n*=12)<br>Setting: MD<br>Monitoring: blood pressure, heart rate, oxygen saturation (*n*=12)<br>Dental procedure: restorative dental treatment (*n*=12)<br>Sedation type	scales: Modified Observer Assessment of Alertness and Sedation (MOAAS) (*n*=12)<br><br>Behavior score and scales: Houpt (*n*=12)<br><br>Adverse events/complications: None (*n*=12)<br>Management of adverse events/complications: N/A<br><br>Procedure completion: MD<br>Reason for interruption: N/A	
		15<br>3.48 ± 0.25 yo, range 2-5 yo (*n*=15) MD<br>MD<br>None (*n*=15)	Meperidine (*n*=15)<br>IM (*n*=15)<br>0.5 mg/kg (*n*=15)<br>Onset (mean min): MD<br>Duration: MD<br>Sleep: N/d<br>Recovery time/score: MD<br>Provider: N/D<br>Setting: MD<br>Monitoring: precordial stethoscope, automatic sphygmomanometer, pulse oximeter (*N*=15)<br>Dental procedure: restorative dental treatment (*n*=15)<br>Sedation type	Nr. of reported effective sedations: MD<br>Sedation score:<br>N/d<br><br>scales: Modified Houpt (*n*=15)<br><br>Behavior score and scales: Dichotomous behavior scale, 10-point behavior scale, global rating scale<br><br>Adverse events/complications: sleep, drowsiness (*n*= N/d)<br>Management of adverse events/complications: MD<br><br>Procedure completion: yes (*n*=14), no (*n*=1)<br>Reason for interruption: unmanageable behavior (*n*=1)	MD<br>MD<br>MD
		15<br>2.99 ± 0.23 yo, range 2-5 yo (*n*=15)<br>MD	Meperidine (*n*=15)<br>IM (*n*=15)<br>1 mg/kg (*n*=15)<br>Onset (mean min): MD	Nr. of reported effective sedations:<br>Sedation score:<br>N/d	

		MD<br>None (*n*=15)	Duration: MD<br>Sleep: N/d<br>Recovery time/score: MD<br>Provider: N/D<br>Setting: MD<br>Monitoring: precordial stethoscope, automatic sphygmomanometer, pulse oximeter (*N*=15)<br>Dental procedure: restorative dental treatment (*n*=15)<br>Sedation type	scales: Modified Houpt (*n*=15)<br><br>Behavior score and scales: Dichotomous behavior scale, 10-point behavior scale, global rating scale<br><br>Adverse events/complications: sleep, drowsiness; nausea/vomiting; hyperexcitement (*n*= N/d)<br>Management of adverse events/complications: MD<br><br>Procedure completion: yes (*n*=15), no (*n*=0)<br>Reason for interruption: N/A	
		15<br>3.58 ± 0.23 yo, range 2-5 yo (*n*=15)<br>MD<br>MD<br>None (*n*=15), uncooperative (Frankl's/definitely negative)	Meperidine (*n*=15)<br>IM (*n*=15)<br>2 mg/kg (*n*=15)<br>Onset (mean min): MD<br>Duration: MD<br>Sleep: N/d<br>Recovery time/score: MD<br>Provider: N/D<br>Setting: MD<br>Monitoring: precordial stethoscope, automatic sphygmomanometer, pulse oximeter (*N*=15)<br>Dental procedure: restorative dental treatment (*n*=15)<br>Sedation type	Nr. of reported effective sedations:MD<br>Sedation score:<br>N/d<br><br>scales: Modified Houpt (*n*=15)<br><br>Behavior score and scales: Dichotomous behavior scale, 10-point behavior scale, global rating scale<br><br>Adverse events/complications: nausea/vomiting (*n*= N/d)<br>Management of adverse events/complications: MD<br><br>Procedure completion: yes (*n*=15), no (*n*=0)<br>Reason for interruption: N/A	
	RCT	15<br>3.6 yo<br>11M/4F<br>None (*n*=15)	N_2O (*n*=15)<br>Inhalation (*n*=15)<br>MD<br>Onset (mean min): MD	Nr. of reported effective sedations: MD<br>Sedation score:<br>MD	

			Duration: MD<br>Sleep: N/d<br>Recovery time/score: MD<br>Provider: research assistant (*n*=15)<br>Setting: MD<br>Monitoring: cardiovascular and respiratory parameters (*n*=15)<br>Dental procedure: N/d dental procedure (*n*=15)<br>Sedation type	scales: MD<br><br>Behavior score and scales: Dichotomous behavior assessment<br><br>Adverse events/complications: none (*n*=15)<br>Management of adverse events/complications: none (*n*=15)<br><br>Procedure completion: yes (*n*=15); no (*n*=0)<br>Reason for interruption: N/A	
	RCT	16<br>2.31 ±0.46 yo<br>MD<br>9M/7F<br>None (*n*=16)	Midazolam (*n*=16)<br>PO (*n*=16)<br>1 mg/kg (*n*=16)<br>Onset (mean min): MD<br>Duration: MD<br>Sleep: N/d<br>Recovery time/score: MD<br>Provider: MD<br>Setting: MD<br>Monitoring: MD<br>Dental procedure: N/d dental procedure (*n*=16)<br>Sedation type	Nr. of reported effective sedations: MD<br>Sedation score:<br>MD<br><br>scales: MD<br><br>Behavior score and scales: OSUBRS (*n*=16)<br>Adverse events/complications: none (*n*=16)<br>Management of adverse events/complications: MD<br><br>Procedure completion: yes (*n*=16); no (*n*=0)<br>Reason for interruption: N/A	MD<br>MD<br>MD
	RCT	20<br>MD<br>MD<br>MD<br>None (*n*=20)	Midazolam (*n*=20)<br>PO (*n*=20)<br>0.25 mg/kg (*n*=20)<br>Onset (mean min): MD<br>Duration: MD<br>Sleep: N/d<br>Recovery time/score: MD<br>Provider: dental nurse (*n*=20)	Nr. of reported effective sedations: MD<br>Sedation score:<br>MD<br><br>scales: MD<br><br>Behavior score and scales: Houpt (*n*=20)	MD<br>MD<br>MD

			Setting: MD<br>Monitoring: pulse oximeter, precordial stethoscope (*n*=20)<br>Dental procedure: N/d dental procedure (*n*=20)<br>Sedation type	Mean score: 5.1± 0.7<br>Adverse events/complications: none (*n*=20)<br>Management of adverse events/complications: MD<br><br>Procedure completion: yes (*n*=20); no (*n*=0)<br>Reason for interruption: MD	
	RCT	N/D<br>3-6 yo<br>MD<br>MD<br>None (*n*=20)	N_2O (*n*=N/d)<br>Ihnalation (*n*=N/d)<br>MD<br>Onset (mean min): MD<br>Duration: MD<br>Sleep: N/d<br>Recovery time/score: MD<br>Provider: anaesthesist (*n*=N/d)<br>Setting: MD<br>Monitoring: MD<br>Dental procedure: N/d dental procedure (*n*=N/d)<br>Sedation type	Sedation efficacy:<br>Sedation score:<br>MD<br><br><br>scales: Vehnahm scale<br><br>Behavior score and scales: BehaviPO screening instrument, N/d score<br>Adverse events/complications: MD<br>Management of adverse events/complications: MD<br><br>Procedure completion: yes (*n*= N/d); no (*n*=0)<br>Reason for interruption: MD	MD<br>MD<br>MD
	RCT	50<br>4.3 ± 1 yo<br>24M/26F<br>MD<br>None (*n*=50)	Midazolam (*n*=50)<br>PR (*n*=50)<br>1 mg/kg (*n*=50)<br>Onset: MD<br>Duration: MD<br>Sleep: N/d<br>Recovery time/score: N/d<br>Provider: N/d research member (*n*=50)<br>Setting: MD<br>Monitoring: pulse oximeter (*n*=50)	Nr. of reported effective sedations: MD<br><br>scales: Ramsay Sedation Score, Movement, crying, overall sedation, and behavior<br>score "orientated and calm" (*n*=7)<br><br>Behavior score and scales: Ramsay Sedation Score, Movement, crying, overall sedation, and behavior<br>score "orientated and calm" (*n*=36)<br>score "no movement" (*n*=7)<br>N/d (*n*=43)<br>Mean score: 3.6 ± 0.6	MD<br>MD<br>MD

				adverse events/complications: hallucination (*n*=21)<br><br>Management of adverse events/complications: MD<br><br>Procedure completion: yes (*n*=49); no (*n*=1)<br>Reason for interruption: NA	
	RCT	30<br>4.9 ± 1.3 yo<br>16M/14F<br>MD<br>None (*n*=30)	Ketamine (*n*=30)<br>PO (*n*=30)<br>12.5 mg/kg (*n*=30)<br>Onset: MD<br>Duration: MD<br>Sleep: N/d<br>Recovery time/score: N/d<br>Provider: N/d research member (*n*=30)<br>Setting: MD<br>Monitoring: pulse oximeter (*n*=30)<br>Dental procedure: tooth extraction (*n*=30)	Nr. of reported effective sedations: 28<br>success rate (%): MD<br><br>scales: 4-point scale for level of sedation (*n*=30)<br>"very good/ excellent sedation" (*n*=28)<br><br>Behavior score and scales: anxiety, movement, crying, overall behavior<br>"good/better behavior" (*n*=28)<br><br>adverse events/complications: hallucination (*n*=5); restless/irritation (*n*=1); N/d (*n*=29)<br><br>Management of adverse events/complications: MD<br><br>Procedure completion: yes (*n*=30)<br>Reason for interruption: NA	MD<br>MD<br>MD
	RCT	10<br>4-7 yo<br>MD<br>MD<br>None (*n*=10)	Midazolam (*n*=10)<br>IN (*n*=10)<br>0.2 mg/kg (*n*=10)<br>Onset: MD<br>Duration: MD<br>Sleep: N/d	Nr. of reported effective sedations: MD<br>success rate (%): MD<br><br>scales: MD<br>Behavior score and scales: Vehnam's clinical anxiety scale; cortisol level	MD<br>MD<br>MD

			Recovery time/score: MD<br>Provider: N/d<br>Setting: MD<br>Monitoring: MD<br>Dental procedure: N/d dental procedure requiring local anesthesia (*n*=10)	Significant decrease in anxiety scores (t1-t4)<br><br>adverse events/complications: MD<br><br>Management of adverse events/complications: MD<br><br>Procedure completion: MD<br>Reason for interruption: NA	
		10<br>4-7 yo<br>MD<br>MD<br>None (*n*=10)	Midazolam (*n*=10)<br>SL (*n*=10)<br>0.2 mg/kg (*n*=10)<br>Onset: MD<br>Duration: MD<br>Sleep: N/d<br>Recovery time/score: MD<br>Provider: N/d<br>Setting: MD<br>Monitoring: MD<br>Dental procedure: N/d dental procedure requiring local anesthesia (*n*=10)	Nr. of reported effective sedations: MD<br>success rate (%): MD<br><br>scales: MD<br>Behavior score and scales: Vehnam's clinical anxiety scale; cortisol level<br>Not significant decrease in anxiety scores (t1-t4)<br><br>adverse events/complications: MD<br><br>Management of adverse events/complications: MD<br><br>Procedure completion: MD<br>Reason for interruption: NA	
	RCT	20<br>5.1 ± 1.07 yo (*n*=20)<br>12M/8F<br>17.5 ± 4.39 (*n*=20)<br>None (*n*=20)	Midazolam (*n*=20)<br>IN (*n*=20)<br>0.2 mg/kg (*n*=20)<br>Onset: MD<br>: MD<br>Duration: MD<br>Sleep: N/d<br>Recovery time/score: MD<br>Provider: N/d<br>Setting: MD<br>Monitoring: MD	Nr. of reported effective sedations: MD<br><br>scales: MD<br>Behavior score and scales: Vehnam's clinical anxiety scale<br>Not significant decrease in anxiety scores (t1-t4)<br>Mean score: 0.35 ± 0.59<br><br>adverse events/complications: MD<br><br>Management of adverse events/complications: MD	MD<br>Child acceptance (Al-Rakaf scale): (*n*=20)<br>MD

			Dental procedure: N/d<br>dental procedure requiring local anesthesia (*n*=20)	Procedure completion: MD<br>Reason for interruption: NA	
		20<br>5.2 ± 1.15 yo (*n*=20)<br>12M/8F<br>17.4 ± 4.33 (*n*=20)<br>None (*n*=20)	Midazolam (*n*=20)<br>SL (*n*=20)<br>0.2 mg/kg (*n*=20)<br>Onset: MD<br>: MD<br>Duration: MD<br>Sleep: N/d<br>Recovery time/score: MD<br>Provider: N/d<br>Setting: MD<br>Monitoring: MD<br>Dental procedure: N/d<br>dental procedure requiring local anesthesia (*n*=20)	Nr. of reported effective sedations: success rate (%): MD<br><br>scales: MD<br>Behavior score and scales: Vehnam's clinical anxiety scale<br>Not significant decrease in anxiety scores (t1-t4)<br>Mean score: 0.45 ± 1.10<br><br>adverse events/complications: MD<br><br>Management of adverse events/complications: MD<br><br>Procedure completion: MD<br>Reason for interruption: NA	Child acceptance (Al-Rakaf scale): (*n*=8)<br>MD<br>MD
	RCT	20<br>3.4 ± 0.6 yo<br>11M/9F<br>12.2 ±1.2 kg<br>MD	Midazolam (*n*=20)<br>IM (*n*=20)<br>0.2 mg/kg (*n*=20)<br>Onset: mean onset: 15.7 ± 2.0 min (*n*=20)<br>Duration: MD<br>Sleep: N/d<br>Recovery time/score: MD<br>Provider: N/d<br>Setting: MD<br>Monitoring: heart rate, respiratory rate (*n*=20)<br>Dental procedure: N/d<br>dental procedure (*n*=20)	Nr. of reported effective sedations:<br><br>scales: MD<br>Behavior score and scales: Houpt, Fukuta scale (7-point scale ranging from asleep to violent)<br>Mean score: 2.2 ± 0.5<br><br>adverse events/complications: sneezing/coughing/hiccups (*n*=2); n7D (*N*=18)<br>Management of adverse events/complications: MD<br><br>Procedure completion: MD<br>Reason for interruption: NA	MD<br>MD<br>MD

		20<br>3.5 ± 0.7 yo<br>8M/12F<br>12.6 ±1.4 kg<br>MD	Midazolam (*n*=20)<br>IN (*n*=20)<br>0.2 mg/kg (*n*=20)<br>Onset: mean onset: 10.8 ± 2.0 min (*n*=20)<br>: MD<br>Duration: MD<br>Sleep: N/d<br>Recovery time/score: MD<br>Provider: N/d<br>Setting: MD<br>Monitoring: heart rate, respiratory rate (*n*=20)<br>Dental procedure: N/d<br>dental procedure (*n*=20)	Nr. of reported effective sedations: MD<br><br>scales: MD<br>Behavior score and scales: Houpt, Fukuta scale (7-point scale ranging from asleep to violent)<br>Mean score: 2.2 ± 0.6<br><br>adverse events/complications: sneezing/coughing/hiccups (*n*=6); N/d (*n*=14)<br>Management of adverse events/complications: MD<br><br>Procedure completion: MD<br>Reason for interruption: NA	
	RCT	30<br>3-9 yo<br>MD<br>MD<br>None (*n*=30)	Midazolam (*n*=30)<br>PO (*n*=30)<br>0.5 mg/kg (*n*=30)<br>Onset: mean onset: MD<br>: MD<br>Duration: MD<br>Sleep: MD<br>Recovery time/score: MD<br>Provider: N/d<br>Setting: MD<br>Monitoring: blood pressure, heart rate, respiratory rate (*n*=20)<br>Dental procedure: N/d<br>dental short procedure (*n*=20)	Nr. of reported effective sedations: MD<br><br>scales: Degree of sedation<br>Mean score: 3.3 ± 0.7 (*n*=30)<br>Behavior score and scales: MD<br><br>adverse events/complications: MD<br>Management of adverse events/complications: NA<br><br>Procedure completion: MD<br>Reason for interruption: NA	MD<br>MD<br>MD
		30<br>3-9 yo<br>MD<br>MD<br>None (*n*=30)	Triclofos (*n*=30)<br>PO (*n*=30)<br>70 mg/kg (*n*=30)<br>Onset: mean onset: MD<br>: MD<br>Duration: MD<br>Sleep: MD	Nr. of reported effective sedations: md<br><br>scales: Degree of sedation<br>Mean score: 3.07 ± 0.6<br>Behavior score and scales: MD<br><br>adverse events/complications: MD	

			Recovery time/score: MD<br>Provider: N/d<br>Setting: MD<br>Monitoring: blood pressure, heart rate, respiratory rate (*n*=20)<br>Dental procedure: N/d dental short procedure (*n*=20)	Management of adverse events/complications: NA<br><br>Procedure completion: MD<br>Reason for interruption: NA	
		30<br>3-9 yo<br>MD<br>MD<br>None (*n*=30)	Promethazine (*n*=30)<br>PO (*n*=30)<br>12.2 mg/kg (*n*=30)<br>Onset: mean onset: MD<br>: MD<br>Duration: MD<br>Sleep: MD<br>Recovery time/score: MD<br>Provider: N/d<br>Setting: MD<br>Monitoring: blood pressure, heart rate, respiratory rate (*n*=20)<br>Dental procedure: N/d dental short procedure (*n*=20)	Nr. of reported effective sedations: success rate (%): MD<br><br>scales: Degree of sedation<br>Mean score: 2.73 ± 0.5 (*n*=30)<br>Behavior score and scales: MD<br><br>adverse events/complications: MD<br>Management of adverse events/complications: NA<br><br>Procedure completion: MD<br>Reason for interruption: NA	
	RCT	28<br>6.54 ±1.79 yo (*n*=28)<br>14M/14F<br>18.89 ± 4.33 (*n*=28)<br>None (*n*=28)	Ketamine (*n*=28)<br>PO (*n*=28)<br>8 mg/kg^{-1} (*n*=28)<br>Onset: mean onset: N/d<br>: MD<br>Duration: MD<br>Sleep: MD<br>Recovery time/score: N/d<br>Provider: N/d<br>Setting: MD<br>Monitoring: oxygen saturation, respiratory rate, blood pressure, respiratory rate (*n*=28)	Nr. of reported effective sedations: MD<br><br>scales: Sedation rating scale modified AAPD<br>Behavior score and scales: FLACC, behavior score<br><br>adverse events/complications: vomiting (*n*=5); emergency reaction (*n*=2)<br>Management of adverse events/complications: MD<br><br>Procedure completion: N/d<br>Reason for interruption: NA	MD<br>MD<br>MD

			Dental procedure: N/d<br>dental procedure (*n*=28)		
		28<br>6.93 ± 2.05 yo (*n*=28)<br>13M/15F<br>17.04 ± 5.33 (*n*=28)<br>None (*n*=28)	Dexmedetomidine (*n*=28)<br>PO (*n*=28)<br>3 μg/kg^{-1} (*n*=28)<br>Onset: mean onset: N/d<br>: MD<br>Duration: MD<br>Sleep: MD<br>Recovery time/score: N/d<br>Provider: N/d<br>Setting: MD<br>Monitoring: oxygen saturation, respiratory rate, blood pressure, respiratory rate (*n*=28)<br>Dental procedure: N/d<br>dental procedure (*n*=28)	Nr. of reported effective sedations: success rate (%): MD<br><br>scales: Sedation rating scale modified AAPD<br>Behavior score and scales: FLACC, behavior score<br><br>adverse events/complications: none (*n*=28)<br>Management of adverse events/complications: NA<br><br>Procedure completion: N/d<br>Reason for interruption: NA	
		28<br>7.21 ± 1.98 yo (*n*=28)<br>11M/17F<br>16.93 ± 4.22 (*n*=28)<br>None (*n*=28)	Dexmedetomidine (*n*=28)<br>PO (*n*=28)<br>4 μg/kg^{-1} (*n*=28)<br>Onset: N/d<br>: MD<br>Duration: MD<br>Sleep: MD<br>Recovery time/score: mean MD<br>Provider: N/d<br>Setting: MD<br>Monitoring: oxygen saturation, respiratory rate, blood pressure, respiratory rate (*n*=28)<br>Dental procedure: N/d<br>dental procedure (*n*=28)	Nr. of reported effective sedations:<br><br>scales: Sedation rating scale modified AAPD<br>Behavior score and scales: FLACC, behavior score<br><br>adverse events/complications: none (*n*=28)<br>Management of adverse events/complications: NA<br><br>Procedure completion: N/d<br>Reason for interruption: NA	
		28<br>6.82 ± 2.22 yo (*n*=28)<br>14M/14F<br>16.61 ± 4.92 (*n*=28)	Dexmedetomidine (*n*=28)<br>PO (*n*=28)<br>5 μg/kg^{-1} (*n*=28)<br>Onset: mean onset: MD	Nr. of reported effective sedations: 23<br>scales: Sedation rating scale modified AAPD	

		None (*n*=28)	: MD<br>Duration: MD<br>Sleep: MD<br>Recovery time/score: N/d<br>Provider: MD<br>Setting: MD<br>Monitoring: oxygen saturation, respiratory rate, blood pressure (*n*=28)<br>Dental procedure: N/d dental procedure (*n*=28)	Behavior score and scales: FLACC, behavior score<br><br>adverse events/complications: vomiting (*n*=1)<br>Management of adverse events/complications: MD<br><br>Procedure completion: N/d<br>Reason for interruption: NA	
	RCT	30<br>5.6 ± 1.85 yo, range 3-10 yo (*n*=30)<br>MD<br>19.2 ± 3.68 kg (*n*=30)<br>MD	Midazolam (*n*=30)<br>PO (*n*=30)<br>0.5 mg/kg (*n*=30)<br>Onset: MD<br>Duration: MD<br>Sleep: MD<br>Recovery time/score: N/d<br>Provider: MD<br>Setting: MD<br>Monitoring: oxygen saturation, respiratory rate, blood pressure (*n*=30)<br>Dental procedure: N/d dental procedure (*n*=30)	Nr. of reported effective sedations: MD<br><br>scales: Wisconsin sedation scale<br>Behavior score and scales: Houpt behaviPO rating scale (N/d)<br><br>adverse events/complications: MD<br>Management of adverse events/complications: NA<br><br>Procedure completion: yes (*n*=24); no (*n*=6)<br>Reason for interruption: MD	MD<br>MD<br>MD
		30<br>5.6 ± 1.67 yo, range 3-10 yo (*n*=90)<br>MD<br>19.7 ± 3.38 kg (*n*=30)<br>MD	Midazolam (*n*=30)<br>PO (*n*=30)<br>0.75 mg/kg (*n*=30)<br>Onset: MD<br>Duration: MD<br>Sleep: MD<br>Recovery time/score: N/d<br>Provider: MD<br>Setting: MD<br>Monitoring: oxygen saturation, respiratory rate, blood pressure (*n*=28)<br>Dental procedure: N/d dental procedure (*n*=30)	Nr. of reported effective sedations:<br><br>scales: Wisconsin sedation scale<br>Behavior score and scales: Houpt behaviPO rating scale (N/d)<br><br>adverse events/complications: MD<br>Management of adverse events/complications: NA<br><br>Procedure completion: yes (*n*=28); no (*n*=2)<br>Reason for interruption: MD	

		30<br>6.2 ± 2.00 yo, range 3-10 yo (*n*=30)<br>MD<br>20.3 ± 3.65 kg (*n*=30)<br>MD	Midazolam (*n*=30)<br>PO (*n*=30)<br>1 mg/kg (*n*=30)<br>Onset: MD<br>Duration: MD<br>Sleep: MD<br>Recovery time/score: N/d<br>Provider: N/d<br>Setting: MD<br>Monitoring: oxygen saturation, respiratory rate, blood pressure (*n*=30)<br>Dental procedure: N/d dental procedure (*n*=30)	Nr. of reported effective sedations: MD<br><br>scales: Wisconsin sedation scale<br>Behavior score and scales: Houpt behaviPO rating scale (N/d)<br><br>adverse events/complications: MD<br>Management of adverse events/complications: NA<br><br>Procedure completion: MD<br>Reason for interruption: NA	
	RCT	21<br>7.34 ± 2.34 yo; 4-12 yo (*n*=21)<br>MD<br>18.29 ± 3.04 kg (*n*=21)<br>None (*n*=21)	Dexmedetomidine (*n*=21)<br>IN (*n*=21)<br>1μg/kg (*n*=21)<br>Onset: N/d<br>Duration: MD<br>Sleep: MD<br>Recovery time/score: N/d<br>Provider: anaesthesist (*n*=21)<br>Setting: MD<br>Monitoring: oxygen saturation, respiratory rate, blood pressure (*n*=21)<br>Dental procedure: N/d dental procedure (*n*=21)	Nr. of reported effective sedations: MD<br><br>scales: Modified AAPD Sedation record<br>Behavior score and scales: FLACC scale (N/d)<br><br>adverse events/complications: MD<br>Management of adverse events/complications: NA<br><br>Procedure completion: MD<br>Reason for interruption: NA	MD<br>MD<br>MD
		21<br>6.71 ± 2.31 yo; 4-11 yo (*n*=21)<br>MD<br>16.52 ± 3.87 kg (*n*=21)<br>None (*n*=21)	Dexmedetomidine (*n*=21)<br>IN (*n*=21)<br>1.5 μg/kg (*n*=21)<br>Onset: MD<br>Duration: MD<br>Sleep: MD<br>Recovery time/score: N/d<br>Provider: anaesthesist (*n*=21)<br>Setting: MD	Nr. of reported effective sedations: success rate (%): MD<br><br>scales: Modified AAPD Sedation record<br>Behavior score and scales: FLACC scale (N/d)<br><br>adverse events/complications: MD<br>Management of adverse events/complications: NA	

			Monitoring: oxygen saturation, respiratory rate, blood pressure (*n*=21)<br>Dental procedure: N/d dental procedure (*n*=21)	Procedure completion: MD<br>Reason for interruption: NA	
		21<br>7.76 ± 2.26 yo; 4-11 yo (*n*=21)<br>MD<br>18.57 ± 4.17 kg (*n*=21)<br>None (*n*=21)	Midazolam (*n*=21)<br>IN (*n*=21)<br>0.2 mg/kg (*n*=21)<br>Onset: MD<br>Duration: MD<br>Sleep: MD<br>Recovery time/score: N/d<br>Provider: anaesthesist (*n*=21)<br>Setting: MD<br>Monitoring: oxygen saturation, respiratory rate, blood pressure (*n*=21)<br>Dental procedure: N/d dental procedure (*n*=21)	Nr. of reported effective sedations: success rate (%): MD<br><br>scales: Modified AAPD Sedation record<br>Behavior score and scales: FLACC scale (N/d)<br><br>adverse events/complications: MD<br>Management of adverse events/complications: NA<br><br>Procedure completion: MD<br>Reason for interruption: NA	
		21<br>7.24 ± 2.36 yo; 4-11 yo (*n*=21)<br>MD<br>17.71 ± 5.36 kg (*n*=21)<br>None (*n*=21)	Ketamine (*n*=21)<br>IN (*n*=21)<br>5 mg /kg (*n*=21)<br>Onset: MD<br>Duration: MD<br>Sleep: MD<br>Recovery time/score: N/d<br>Provider: anaesthesist (*n*=21)<br>Setting: MD<br>Monitoring: oxygen saturation, respiratory rate, blood pressure (*n*=21)<br>Dental procedure: N/d dental procedure (*n*=21)	Nr. of reported effective sedations: MD<br><br>scales: Modified AAPD Sedation record<br>Behavior score and scales: FLACC scale (N/d)<br><br>adverse events/complications: MD<br>Management of adverse events/complications: NA<br><br>Procedure completion: MD<br>Reason for interruption: NA	
	RCT	18<br>3.9 yo, 4-9 yo (*n*=18)<br>11M/7F<br>Mean 18.1 kg<br>MD	Hydroxyzine (*n*=18)<br>PO (*n*=18)<br>2 mg/kg (2h before); 1 mg/kg (20 min before)<br>Onset: MD<br>Duration: MD	Nr. of reported effective sedations: MD<br><br>scales: MD<br>Behavior score and scales: Ohio State BehaviPO Rating Scale	MD<br>MD<br>MD

			Sleep: MD<br>Recovery time/score: MD<br>Provider: anaesthesist (*n*=18)Setting: MD<br>Monitoring: respiratory rate, heart rate (mean value n152) (*n*=18)<br>Dental procedure: N/d dental procedure (*n*=18)	adverse events/complications: MD<br>Management of adverse events/complications: NA<br><br>Procedure completion: MD<br>Reason for interruption: NA	
	RCT	40<br>2-10 yo<br>MD<br>MD<br>MD	Midazolam (*n*=10)<br>PO (*n*=10)<br>0.5 mg/kg<br>Onset: MD<br>Duration: MD<br>Sleep: MD<br>Recovery time/score: MD<br>Provider: anaesthesist (*n*=10)<br>Setting: MD<br>Monitoring: respiratory rate, oxygen saturation, blood pressure (*n*=10)<br>Dental procedure: N/d dental procedure (*n*=10)	Nr. of reported effective sedations: MD<br><br>scales: MD<br>Behavior score and scales: Houpt, Child behavior question<br>mean Houpt score: 4.9 ± 0.3 (*n*=10)<br><br>adverse events/complications: MD<br>Management of adverse events/complications: NA<br><br>Procedure completion: MD<br>Reason for interruption: NA	MD<br>MD<br>MD
			Diazepam (*n*=10)<br>PO (*n*=10)<br>0.5 mg/kg<br>Onset: MD<br>Duration: MD<br>Sleep: MD<br>Recovery time/score: MD<br>Provider: anaesthesist (*n*=10)<br>Setting: MD<br>Monitoring: respiratory rate, oxygen saturation, blood pressure (*n*=10)<br>Dental procedure: N/d dental procedure (*n*=10)	Nr. of reported effective sedations: success rate (%): MD<br><br>scales: MD<br>Behavior score and scales: Houpt, mean Houpt score: 4.5 ± 0.5 (*n*=10)<br><br>adverse events/complications: MD<br>Management of adverse events/complications: NA<br><br>Procedure completion: MD<br>Reason for interruption: NA	
			Midazolam (*n*=10)<br>IV (*n*=10)	Nr. of reported effective sedations: success rate (%): MD	

			0.06 mg/kg<br>Onset: MD<br>:<br>Duration: MD<br>Sleep: MD<br>Recovery time/score: MD<br>Provider: anaesthesist (*n*=10)<br>Setting: MD<br>Monitoring: respiratory rate, heart rate (mean value 137) (*n*=18)<br>Dental procedure: N/d dental procedure (*n*=10)	scales: MD<br>Behavior score and scales: Houpt, Child behavior question<br>mean Houpt score: 5.8 ± 0.4<br><br>adverse events/complications: MD<br>Management of adverse events/complications: NA<br><br>Procedure completion: MD<br>Reason for interruption: NA	
	RCT	56<br>6-11 yo<br>MD<br>MD<br>None (*n*=56)	N_2O (*n*=29)<br>Ihnalation (*n*=29)<br>Up to 40/60 (%N_2/O_2) (*n*=29)<br>Onset: MD<br>Duration: MD<br>Sleep: MD<br>Recovery time/score: MD<br>Provider: dentist (*n*=29)<br>Setting: MD<br>Monitoring: MD<br>Dental procedure: N/d dental procedure (*n*=29)	Nr. of reported effective sedations: success rate (%): MD<br><br>scales: Vehnam scale<br>mean value: 3.45 ± 0.92 (*n*=29)<br>Behavior score and scales: MD<br><br>adverse events/complications: None (*n*=27)<br>Management of adverse events/complications: NA<br><br>Procedure completion: yes (*n*=29)<br>Reason for interruption: NA	MD<br>MD<br>MD
	RCT	20<br>7.3 yo, 5-10 yo<br>MD<br>MD<br>None (*n*=20)	Midazolam (*n*=20)<br>PO (*n*=20)<br>Onset: MD<br>Duration: MD<br>Sleep: MD<br>Recovery time/score: MD<br>Provider: MD<br>Setting: MD<br>Monitoring: MD<br>Dental procedure: N/d dental procedure (*n*=20)	Nr. of reported effective sedations: success rate (%): MD<br><br>scales: Ramsay scale<br>Behavior score and scales: Brietkopf and Buttner, Frankl, Houpt<br>mean Houpt: 4.7 ± 1.2 (*n*=20)<br>adverse events/complications: None (*n*=20)<br>Management of adverse events/complications: NA	MD<br>MD<br>MD

				Procedure completion: MD<br>Reason for interruption: NA	
Chen<br>2020<br>Dovepress<br>[17]<br>2 RCT<br>Meta-analysis					
	RCT	20<br>3-7 yo<br>MD<br>16 kg<br>MD	ChlPO hydrate (*n*=9)<br>PO (*n*=9)<br>40 mg/kg (*n*=9)<br>Onset: MD<br>Duration: MD<br>Sleep: MD<br>Recovery: MD<br>Provider: MD<br>Setting: MD<br>Dental procedure: dental examination	Nr. of reported effective sedations: (*n*=6);<br><br>Sedation score and scale: N/d<br><br>Behavior score and scale: MD<br><br>Adverse events/complications: MD<br><br>Management of adverse events/complications: MD<br><br>Procedure completion: MD<br>Reason for interruption: MD	MD<br>MD<br>MD
			Diazepam (*n*=11)<br>PO (*n*=11)<br>5mg (*n*=11)<br>Onset: MD<br>Duration: MD<br>Sleep: MD<br>Recovery: MD<br>Provider: MD<br>Setting: MD<br>Dental procedure: dental examination	Nr. of reported effective sedations: (*n*=5);<br>Sedation score and scale: N/d<br><br>Behavior score and scale: MD<br><br>Adverse events/complications: MD<br><br>Management of adverse events/complications: MD<br><br>Procedure completion: MD	

				Reason for interruption: MD	
	RCT	232<br>0.1-6 yo<br>MD<br>MD<br>MD	ChlPO hydrate (*n*=120)<br>PO (*n*=120),<br>0.8-1.0 mL/µg (*N*=120),<br>Onset: MD<br>Duration: MD<br>Sleep: MD<br>Recovery: MD<br>Provider: MD<br>Setting: MD<br>Dental procedure: dental examination	Effective sedations (*n*=90)<br><br>Sedation score and scale: N/d<br><br>Behavior score and scale: MD<br><br>Adverse events/complications: MD<br><br>Management of adverse events/complications: MD<br><br>Procedure completion: MD<br>Reason for interruption: MD	MD<br>MD<br>MD
			Phenobarbital (*n*=112)<br>Intramuscular (*n*=112)<br>5mg/kg (*n*=112)<br>Onset: MD<br>Duration: MD<br>Sleep: MD<br>Recovery: MD<br>Provider: MD<br>Setting: MD<br>Dental procedure: dental examination	Nr. of reported effective sedations: (*n*=89)<br><br>Sedation score and scale: N/d<br><br>Behavior score and scale: MD<br><br>Adverse events/complications: MD<br><br>Management of adverse events/complications: MD<br><br>Procedure completion: MD<br>Reason for interruption: MD	

Da Silva<br>2024<br>Revista contribuciones a las ciencias sociales<br>[18]<br>1 RCT<br>No Meta-analysis	RCT	30<br>2-10 yo<br>MD<br>MD<br>MD<br>MD	Midazolam (*n*=10)<br>PO (*n*=10)<br>0.5 mg/kg<br>Onset: MD<br>Duration: MD<br>Sleep: MD<br>Recovery: MD<br>Provider: MD<br>Setting: Dental office<br>N/d dental procedure	Nr. of reported effective sedations: MD<br><br>Sedation score and scale: N/d<br><br>Behavior score and scale: MD<br><br>Adverse events/complications: MD<br><br>Management of adverse events/complications: MD<br><br>Procedure completion: MD<br>Reason for interruption: MD	MD<br>MD<br>MD
			Diazepam (*n*=10)<br>PO (*n*=10)<br>0.5 mg/kg (*n*=10)<br>Onset: MD<br>Duration: MD<br>Sleep: MD<br>Recovery: MD<br>Provider: MD<br>Setting: Dental office<br>N/d dental procedure	Nr. of reported effective sedations: MD<br><br>Sedation score and scale: N/d<br><br>Behavior score and scale: MD<br><br>Adverse events/complications: MD<br><br>Management of adverse events/complications: MD<br><br>Procedure completion: MD<br>Reason for interruption: MD	
			Midazolam (*n*=10)<br>IV (*n*=10)<br>0.06 mg/kg (*n*=10)<br>Onset: MD<br>Duration: MD<br>Sleep: MD<br>Recovery: MD<br>Provider: MD<br>Setting: Dental office	Nr. of reported effective sedations: MD<br><br>Sedation score and scale: N/d<br><br>Behavior score and scale: MD<br><br>Adverse events/complications: MD	

			N/d dental procedure	Management of adverse events/complications: MD<br><br>Procedure completion: MD<br>Reason for interruption: MD	
Goswami<br>2021<br>JISPPD<br>[19]<br>1<br>observational study<br>Meta-analysis					
	Observational study	30<br>MD<br>MD<br>MD<br>None (*n*=30)	Dexmedetomidine (*n*=30)<br>IV (*n*=30)<br>2µg/kg (induction dose, over 5 min), followed by 0.4µg/kg (maintenance dose) (*n*=30)<br>Onset (mean min) MD<br>Duration (mean min MD<br>Recovery time/score (mean, min): N/d<br>Provider: anesthesiologist (*n*=30)<br>Setting: outpatient dental clinic (*n*=30)<br>Monitoring: N/d vital parameters (*n*=30)<br>Dental procedure: teeth extraction (*n*=13);<br>Restorative dental therapy (*n*=17)	Nr. of reported effective sedations:<br>Success rate (%): N/D<br><br>Scales: score 5 of Ramsay sedation scale (*N*=30)<br><br>Behavior score, scales MD<br><br>Adverse events/complications: None (*n*=30)<br><br>Management of adverse events/complications: NA<br><br>Procedure completion: MD<br>Reason for interruption: MD	Acceptability (child/caregiver): MD<br>Satisfaction (child/caregiver/dentist): MD/MD/3-point satisfaction scale (score 1, *n*=0; score 2, *n*=7; score 3, *n*=23)

			Midazolam (*n*=30)<br>IV (*n*=30)<br>0.05 mg/kg (induction dose, over 5 min), followed by 0.06-0.12 mg/kg (maintenance dose) (*n*=30)<br>Onset: MD<br>: MD<br>Duration: MD<br>Recovery time/score: N/d<br>Provider: anesthesiologist (*n*=30)<br>Setting: outpatient dental clinic (*n*=30)<br>Monitoring: N/d vital parameters (*n*=30)<br>Dental procedure: teeth extraction (*n*=11);<br>Restorative dental therapy (*n*=19)	Nr. of reported effective sedations:<br>Success rate (%): N/D<br><br>Scales: score 5 of Ramsay sedation scale (*n*=30)<br><br>Behavior scales, score: MD<br>Adverse events/complications: None (*n*=30)<br><br>Management of adverse events/complications: NA<br><br>Procedure completion: MD<br>Reason for interruption: MD	Acceptability (child/caregiver): MD<br>Satisfaction (child/caregiver/provider): MD/MD/3-point satisfaction scale (score 1, *n*=0; score 2, *n*=9; score 3, *n*=21)

Janiani<br>Pesqui Bras Odontopediatria Clín Integr.<br>[20]<br>7 RCT<br>No meta-analysis					
	RCT	46<br>10-16 yo<br>MD<br>MD<br>MD	Midazolam (*n*=46)<br>PO (*n*=46)<br>0.5 mg/kg (*n*=46)<br>Onset: MD<br>Duration: MD<br>Recovery time/score: MD<br>Provider: MD	MD<br>MD<br><br>Children’s fear survey<br>Schedule dental subscale: baseline (mean): 31<br>Spielberger state anxiety	MD<br>MD<br>MD

			Setting: MD<br>Monitoring: oxygen saturation (*n*=46); respiratory rate (*n*=46)<br>Dental procedure: Tooth extraction (*n*=46)	Inventory: from 20 to 73 (*n*=46)<br>Adverse events/complications: Amnesia (*n*=39)<br>Management of adverse events/complications: MD<br>Procedure completion: Yes (*n*=45); no (*n*=1)<br>Reason for interruption: N/D	
		46<br>10-16 yo<br>MD<br>MD<br>MD	N_2O (*n*=46)<br>Mask (*n*=46)<br>30/70 (N_2O/O_2 %) (*n*=46)<br>Onset: MD<br>: MD<br>Duration: MD<br>Recovery time/score: MD<br>Provider: MD<br>Setting: MD<br>Monitoring: oxygen saturation (*n*=46); respiratory rate (*n*=46)<br>Dental procedure: Tooth extraction (*n*=46)	MD<br>MD<br>Children's fear survey<br>Schedule dental subscale: baseline (mean): 28<br>Spielberger state anxiety<br>Inventory: from 20 to 73<br>Adverse events/complications: Amnesia (*n*=6)<br><br>Management of adverse events/complications: MD<br><br>Procedure completion: Yes (*n*=45); no (*n*=1)<br>Reason for interruption: N/D	
	RCT	26<br>10-16 yo<br>MD<br>MD<br>MD	Midazolam (*n*=26)<br>PO (*n*=26)<br>0.5 mg/kg (*n*=26)<br>Onset: MD<br>: MD<br>Duration: MD<br>Recovery time/score: MD<br>Provider: MD<br>Setting: MD<br>Monitoring: oxygen saturation (*n*=26); respiratory rate (*n*=26)<br>Dental procedure: Tooth extraction (*n*=26)	Reported Nr. of reported effective sedations:MD<br><br>Sedation score, scale : Breitkopf and Buttner-Classification (Level of sedation): score 2, awake and calm (*n*=7); score 3, (*n*=13); score 4 (*n*=4)<br><br>Behavior score, scale: score 6 (excellent, *n*=18); score 5 (very good, *n*=3); score 4 (good, *n*=2); score 3 (fair, *n*=2), Houpt<br><br>Adverse events/complications: amnesia (*n*=20)	MD<br>MD<br>MD

				sleepiness/dizziness/slight headache (*n*=5, 19%)<br>paradoxical reaction (*n*=1)<br>treatment abortion (*n*=1) due to paradoxical reaction<br>Management of adverse events/complications: MD<br>Procedure completion: yes (*n*=25); no (*n*=1)<br>Reason for interruption: paradoxical reaction (*n*=1)	
			N_2O (*n*=26)<br>Mask (*n*=26)<br>30/70 (N_2O/O_2 %) (*n*=26)<br>Onset: MD<br>Duration: MD<br>Recovery time/score: MD<br>Provider: MD<br>Setting: MD<br>Monitoring: oxygen saturation (*n*=26); respiratory rate (*n*=26)<br>Dental procedure: Tooth extraction (*n*=26)	Reported Nr. of reported effective sedations:MD<br>Sedation score, scale: Breitkopf and Buttner-Classification (Level of sedation): score 2, awake and calm (*n*=24); score 3, tired and hardly moving (*n*=2)<br>Reported Nr. of reported effective sedations:MD<br><br>Behavior score, scale: : score 6 (excellent, *n*=21); score 5 (very good, *n*=3); score 4 (good, *n*=1), Houpt<br><br>adverse events/complications: amnesia (*n*=3)<br>sleepiness/dizziness/slight headache (*n*=5, 19%)<br><br>Management of adverse events/complications: MD<br><br>Procedure completion: yes (*n*=25); no (*n*=1)<br>Reason for interruption: MD	

	RCT	42<br>12-16 yo<br>MD<br>MD<br>MD	Midazolam (*n*=42)<br>IV (*n*=42)<br>0.5 mg min, max 5 mg (*n*=42)<br>Median onset to max sedation: 8 min, range 4-20 min (*n*=42)<br>Onset: MD<br>Duration: MD<br>Recovery time/score: MD<br>Provider: MD<br>Setting: MD<br>Mean recovery: 51.6 min (*n*=42)<br>Provider: Specialist trained in pediatric sedation and life support (*n*=42)<br>Monitoring: N/d oxygen saturation; N/d blood pressure; N/d pulse rate; N/d ventilatory frequency (*n*=42)<br>Dental procedure: tooth extraction (*n*=42)	Reported Nr. of reported effective sedations:MD<br>Sedation score, scale:<br>Breitkopf and Buttner-Classification (Level of sedation) N/d scores<br><br>Behavior score, scale: Houpt, Frankl scale N/d scores<br><br>adverse events/complications: Nausea, vomiting, drowsiness, headache, sore mouth (*n*=14)<br><br>Management of adverse events/complications: MD<br><br>Procedure completion: MD<br>Reason for interruption: MD	MD<br>MD<br>MD
			N_2O (*n*=42)<br>Inhalation (*n*=42)<br>30/70 (N_2O/O_2 %) (*n*=42)<br>Median onset to max sedation: 6 min, range 2-18 min (*n*=42)<br>Duration: MD<br>Recovery time/score: MD<br>Provider: MD<br>Setting: MD<br>Mean recovery: 23.3 min, range 2-18 min (*n*=42)<br>MD<br>MD	Reported Nr. of reported effective sedations:MD<br>Sedation score, scale:<br>Breitkopf and Buttner-Classification (Level of sedation) N/d scores<br><br>Behavior score, scale: Houpt, Frankl scale N/d scores<br><br>Adverse events/complications: Nausea, vomiting, drowsiness, headache, sore mouth (*n*=11)	

			Monitoring: N/d oxygen saturation; N/d blood pressure; N/d pulse rate; N/d respiratory rate (*n*=42)<br>Dental procedure: tooth extraction (*n*=42)	Management of adverse events/complications: MD<br><br>Procedure completion: MD<br>Reason for interruption: MD	
	RCT	42<br>5-10 yo<br>MD<br>MD<br>MD	Midazolam (*n*=42)<br>PO (*n*=42)<br>0.3 mg/kg (*n*=42)<br>Onset: MD<br>Duration: MD<br>Recovery time/score: MD<br>Provider: MD<br>Setting: MD<br>Monitoring: heart rate, respiratory rate, oxygen saturation, blood pressure<br>Dental procedure: Tooth extraction (*n*=42)	Reported Nr. of reported effective sedations:MD<br>Sedation score, scale:<br>Breitkopf and Buttner-Classification (Level of sedation): score 4, (*n*=30)<br><br>Behavior score, scale: Houpt, Frankl scale N/d scores<br><br>Adverse events/complications: Drowsiness, headache (*n*=9); procedural amnesia (N/d)<br><br>Management of adverse events/complications: MD<br><br>Procedure completion: Yes (*n*=37); no (*n*=5)<br>Reason for interruption: Unability to tolerate PO midazolam (*n*=5)	MD<br>MD<br>MD
			N_2O (*n*=42)<br>Ihnalation (*n*=42)<br>30/70 (N_2O/O_2 %) (*n*=42)<br>Onset: MD<br>Duration: MD<br>Recovery time/score: MD<br>Provider: MD	Reported Nr. of reported effective sedations:MD<br>Sedation score, scale: Breitkopf and Buttner-Classification (Level of sedation): score 3,(*n*=36)	

			Setting: MD<br>Monitoring: heart rate, respiratory rate, oxygen saturation, blood pressure<br>Dental procedure: Tooth extraction (*n*=42)	Behavior score, scale: no disruptive behavior (*n*=37), Houpt<br><br>Adverse events/complications: Drowsiness, headache (*n*=9); procedural amnesia (N/d)<br><br>Management of adverse events/complications: MD<br><br>Procedure completion: Yes (*n*=37); no (*n*=5)<br>Reason for interruption: Inability to tolerate PO midazolam (*n*=5)	
	RCT	36<br>10-15 yo<br>MD<br>MD<br>MD	Midazolam (*n*=36)<br>Transmucosal (buccal) (*n*=36)<br>Onset: MD<br>Duration: MD<br>Recovery time/score: MD<br>Provider: MD<br>Setting: MD<br>Monitoring: heart rate, respiratory rate, oxygen saturation, blood pressure<br>Dental procedure: Tooth extraction (*n*=36)	Reported Nr. of reported effective sedations:MD<br>Sedation score, scale: Breitkopf and Buttner-Classification (Level of sedation): score 3 (*n*=36)<br><br>Behavior score, scale: N/d Houpt, Children's fear survey schedule dental subscale (dental anxiety): mean score 39.4 (*n*=36)<br>Spielberg state anxiety inventory (general anxiety): mean score 39.4 (*n*=36)<br><br>Adverse reactions: Sleepiness, headache, slight nausea (*n*=16)<br>Management of adverse reactions/complications: MD<br><br>Procedure completion: MD<br>Reason for interruption: MD	MD<br>MD

			N_2O (*n*=36)<br>Mask (*n*=36)<br>30/70 (N_2O/O_2 %) (*n*=36)<br>Onset: MD<br>Duration: MD<br>Recovery time/score: MD<br>Provider: MD<br>Setting: MD<br>Monitoring: heart rate, respiratory rate, oxygen saturation, blood pressure<br>Dental procedure: Tooth extraction (*n*=36)	Reported of effective sedation: MD<br><br>Sedation score, scale: Breitkopf and Buttner-Classification (Level of sedation): score 3 (*n*=3)<br><br>Behavior scale, score: N/d Houpt;<br>Children's fear survey schedule dental subscale (dental anxiety): score 39.4 (*n*=36);<br>Spielberg state anxiety inventory (general anxiety): mean post-op score 39.4 (*n*=36)<br><br>Adverse reactions/ complications: Sleepiness, headache, slight nausea (*n*=14)<br>Management of adverse reactions/complications: MD<br><br>Procedure completion: MD<br>Reason for interruption: MD	
	RCT	35<br>4-7 yo<br>MD<br>MD<br>MD	Midazolam (*n*=35)<br>Intranasal (*n*=35)<br>0.3 mg/kg (*n*=35)<br>Onset: MD<br>Duration: MD<br>Recovery time/score: MD<br>Provider: MD<br>Setting: MD<br>Monitoring: heart rate, respiratory rate, oxygen saturation, blood pressure<br>Dental procedure: restorative dental therapy (*n*=35)	Nr. of reported effective sedations:MD<br><br>Sedation score, scale: Ellis sedation scale N/d scores (*n*=35)<br>Behavior score/scale: Excellent (*n*=19), Houpt<br>FLACC mean score 2.77 (*n*=35)<br><br>Adverse events/complications: Sneezing, coughing, hiccups (*n*=4)<br>Management of adverse events/complications: MD<br><br>Procedure completion: Yes(*n*=35)<br>Reason for interruption: NA	MD<br>MD<br>MD

			N_2O (*n*=35)<br>Mask (*n*=35)<br>30/70 (N_2O/O_2 %) (*n*=35)<br>Onset: MD<br>Duration: MD<br>Recovery time/score: MD<br>Provider: MD<br>Setting: MD<br>Monitoring: heart rate, respiratory rate, oxygen saturation, blood pressure<br>Dental procedure: restorative dental therapy, (*n*=35)	Nr. of reported effective sedations:MD<br><br>Sedation score, scale: Ellis sedation scale N/d scores (*n*=35)<br>Behavior score, scale: Excellent (*n*=20), Houpt<br>FLACC mean score 1.57 ±2.6 (*n*=35)<br><br>Adverse events/complications: vomiting (*n*=1)<br>Management of adverse events/complications:MD<br><br>Procedure completion: Yes (*n*=35)<br>Reason for interruption: MD	
	RCT	35<br>4-8 yo<br>MD<br>MD<br>MD	Midazolam (*n*=35)<br>Intranasal (*n*=35)<br>0.3 mg/kg (*n*=35)<br>MD<br>Onset: MD<br>Duration: MD<br>Recovery time/score: MD<br>Provider: MD<br>Setting: MD<br>Monitoring: heart rate, respiratory rate, oxygen saturation, blood pressure<br>Dental procedure: restorative dental therapy (*n*=35)	Nr. of reported effective sedations:MD<br><br>Sedation score, scale: Modified Ramsay sedation scale: moderate (*n*=35)<br><br>Behavior score, scale: MD<br><br>Adverse events/complications: Sneezing, coughing, hiccups (*n*=4)<br>Management of adverse events/complications: MD<br><br>Procedure completion: MD<br>Reason for interruption MD	MD<br>MD<br>MD
			N_2O (*n*=35)<br>Ihnalation (*n*=35)<br>30/70 (N_2O/O_2 %) (*n*=35)<br>Onset: MD<br>Duration: MD<br>Recovery time/score: MD<br>Provider: MD<br>Setting: MD	Nr. of reported effective sedations:MD<br><br>Sedation score, scale: Modified Ramsay sedation scale: moderate (*n*=35)<br><br>Behavior score, scale: MD	

			Monitoring: heart rate, respiratory rate, oxygen saturation, blood pressure<br>Dental procedure: restorative dental therapy (*n*=35)	Adverse events/complications: vomiting (*n*=5)<br>Management of adverse events/complications: MD<br><br>Procedure completion: MD<br>Reason for interruption: MD	
Lam<br>2018<br>Journal of emergency medicine<br>[21]<br>4 RCT, 3 observational<br>No Meta-analysis					
	RCT	24<br>2-7 yo<br>MD<br>MD<br>MD	Midazolam (*n*=24)<br>PR (*n*=24)<br>0.3 mg/kg (*n*=24)<br>Onset: MD<br>duration: 180 min<br>Recovery time/score: MD<br>Provider: MD<br>Outpatient dental clinic (*n*=24)<br>Oxygen saturation (*n*=24)<br>N/d dental procedure (*n*=24)	Nr. of reported effective sedations:(*n*=16)<br><br>Sedation score, scale: MD<br><br>Behavior score, scale: MD<br><br>Adverse events/ complications: Amnesia/drowsiness (*n*=N/d); desaturation (<89%) (*n*=2)<br><br>Management of adverse events/complications: MD<br><br>Procedure completion: MD<br>Reason for interruption: MD	MD<br>MD<br>MD
	RCT	100<br>2-7 yo (*n*=100)<br>MD<br>MD<br>MD	Midazolam (*n*=50)<br>PR (*n*=50)<br>1 mg/kg (*n*=50)<br>Onset: MD<br>duration: 180 min	Nr. of reported effective sedations:(*n*=46)<br><br>Sedation score, scale: MD	MD<br>MD<br>MD

			Recovery time/score: MD<br>Provider: MD<br>Outpatient dental clinc (*n*=50)<br>N/d dental procedure (*n*=50)	Behavior score, scale: MD<br><br>Adverse events/ complications: None (*n*=50)<br><br>Management of adverse events/complications: MD<br><br>Procedure completion: MD<br>Reason for interruption: MD	
	Observational study	182<br>1.3-9.3 yo<br>MD<br>MD<br>MD	Midazolam (*n*=91)<br>PR (*n*=91)<br>0.6 mg/kg (*n*=91)<br>Mean onset: 15-33 min (*n*=91)<br>Mean Duration: 45-79 min<br>Recovery time/score: MD<br>Provider: MD<br>Setting: Outpatient dental clinic (*n*=91)<br>Monitoring: oxygen saturation (*n*=91)<br>N/d dental procedure (*n*=50)	Nr. of reported effective sedations:(*n*=59)<br><br>N/d scale: "satisfactory/ better" sedation level (*n*=59)<br><br>Behavior score, scale: MD<br><br>Adverse events/ complications: None (*n*=91)<br><br>Management of adverse events/complications: MD<br><br>Procedure completion: MD<br>Reason for interruption: MD	MD<br>MD<br>MD
			Midazolam (*n*=91)<br>PO (*n*=91)<br>0.7 mg/kg (*n*=91)<br>Mean onset: 15-33 min (*n*=91)<br>MD<br>Mean Duration: 45-79 min<br>Recovery time/score: MD<br>Provider: MD<br>Setting: Outpatient dental clinic (*n*=91)<br>Monitoring: oxygen saturation (*n*=91)	Nr. of reported effective sedations:(*n*=68)<br><br>Sedation score, scale: MD<br><br>Behavior score, scale: MD<br><br>Adverse events/ complications: Paradoxical reactions (*n*=3); oxygen desaturation (*n*=2)	

			N/d dental procedure (*n*=50)	Management of adverse events/complications: Oxygen application (*n*=2)<br><br>Procedure completion: MD<br>Reason for interruption: MD	
	RCT	90<br>1.5-3.5 yo (*n*=90)<br>MD<br>MD<br>MD	Midazolam (*n*=45)<br>PR (*n*=45)<br>0.3 mg/kg (*n*=45)<br>Onset: 10 min (*n*=34)<br>Duration: MD<br>Recovery: MD<br>Provider: MD<br>Outpatient dental clinic (*n*=45)<br>Monitoring: MD<br>Dental procedure: N/d dental procedure (*n*=45)	Sedation success: MD<br><br>Sedation score, scale: MD<br><br>Behavior score, scale: MD<br><br>Adverse events/ complications: MD<br><br>Management of adverse events/complications: MD<br><br>Procedure completion: MD<br>Reason for interruption: MD	MD<br>MD<br>MD
			Diazepam (*n*=45)<br>PR (*n*=45)<br>0.7 mg/kg (*n*=45)<br>Onset: 10 min (*n*=36)<br>Duration: MD<br>Recovery: MD<br>Provider: MD<br>Outpatient dental clinc (*n*=45)<br>MD<br>N/d dental procedure (*n*=45)	Nr. of reported effective sedations:MD<br><br>Sedation score, scale: MD<br><br>Behavior score, scale: MD<br><br>Adverse events/complications: Agitation at 60 min (*n*= N/d); residual aggressiveness and tiredness at 24 h (*n*= N/d)<br><br>Management of adverse events/complications: MD<br><br>Procedure completion: MD<br>Reason for interruption: MD	
	Observational study	225<br>1.4- 10.5 yo (*n*=120)<br>MD<br>MD	Midazolam (*n*=225)<br>PR (*n*=225)<br>0.3 mg/kg, (*n*=225)<br>Onset: MD	Success: MD<br><br>Sedation score, scale: MD	MD<br>MD<br>MD

		MD	Duration: MD<br>Sleep: MD<br>Recovery: MD<br>Provider: MD<br>Setting: Outpatient dental clinc (*n*=225)<br>Saturation level (*n*=225)<br>N/d dental procedure (*n*=225)	Behavior score, scale: MD<br><br>Adverse events/complications: None (*n*=225)<br><br>Management of adverse events/complications: NA<br><br>Procedure completion: Treatment completed without difficulty (*n*=135); with some difficulty (*n*=89)<br>Reason for interruption: MD	
	RCT	50<br>2-9 yo (*n*=50)<br>MD<br>MD<br>MD	Midazolam (*n*=25)<br>PR (*n*=25)<br>0.35 mg/kg (*n*=25)<br>Onset: MD<br>Duration: MD<br>Sleep: MD<br>Recovery: MD<br>Provider: MD<br>Outpatient dental clinic (*n*=25)<br>N/d dental procedure (*n*=25)	Effective sedations(*n*=12)<br>Sedation score, scale: MD<br><br>Behavior score, scale: MD<br><br>Adverse events/complications: MD<br><br>Management of adverse events/complications MD<br><br>Procedure completion: MD<br>Reason for interruption: MD	N/d child acceptance<br>MD<br>MD
			Midazolam (*n*=25)<br>PO (*n*=25)<br>0.5 mg/kg (*n*=25)<br>Onset: MD<br>Duration: MD<br>Sleep: MD<br>Recovery: MD<br>Provider: MD<br>Outpatient dental clinic (*n*=25)<br>N/d dental procedure (*n*=25)	Effective sedations(*n*=12)<br>Sedation score, scale: MD<br><br>Behavior score, scale: MD<br><br>Adverse events/complications: MD<br><br>Management of adverse events/complications: MD<br><br>Procedure completion: MD	

	Observational study	680<br>2-6 yo (*n*=680)<br>MD<br>MD<br>MD	Midazolam (*n*=171)<br>PR (*n*=171)<br>0.3 mg/kg (*n*=171)<br>Onset: MD<br>Duration: MD<br>Sleep: MD<br>Recovery: MD<br>Provider: MD<br>Outpatient dental clinc (*n*=171)<br>N/d dental procedure (*n*=171)	Effective sedations(*n*=12)<br>Sedation score, scale: MD<br><br>Behavior score, scale: MD<br><br>Adverse events/complications: MD<br><br>Management of adverse events/complications: MD<br><br>Procedure completion: MD	MD<br>MD<br>MD
			Midazolam (*n*=509)<br>PO (*n*=509)<br>0.5 mg/kg (*n*=509)<br>Onset: MD<br>Duration: MD<br>Sleep: MD<br>Recovery: MD<br>Provider: MD<br>Outpatient dental clinc (*n*=509)<br>N/d dental procedure (*n*=509)	Effective sedations (*n*=12)<br>Sedation score, scale: MD<br><br>Behavior score, scale: MD<br><br>Adverse events/complications: MD<br><br>Management of adverse events/complications: MD<br><br>Procedure completion: MD	
Lewis<br>2019<br>J Periop practice<br>[22]<br>2 RCT<br>No Meta-analysis					
	RCT	36<br>3-9 yo<br>MD<br>MD<br>MD	Dexmedetomidine (*n*=13)<br>IN (*n*=13)<br>1 µg/kg (*n*=13)<br>Onset: MD<br>Duration: MD<br>Sleep: MD	Nr. of reported effective sedations: (*n*=7)<br><br>Sedation score, scale: Modified Observer’s Assessment of Alertness/ Sedation scale (*n*=13)	MD<br>MD<br>MD

			Recovery: MD<br>Provider:MD<br>Setting: MD<br>Dental procedure: N/d dental procedure (*n*=13)	Behavior score and scale: MD<br><br>Adverse events/complications: MD<br><br>Management of adverse events/complications: MD<br><br>Procedure completion: MD<br>Reason for interruption: MD	
			Midazolam (*n*=12)<br>PO (*n*=12)<br>0.5 mg/kg (*n*=12)<br>Onset: MD<br>Duration: MD<br>Sleep: MD<br>Recovery: MD<br>Provider:MD<br>Setting: MD<br>N/d dental procedure (*n*=12)	Nr. of reported effective sedations: (*n*=8)<br><br>Sedation score, scale: Modified Observer's Assessment of Alertness/ Sedation scale (*n*=12)<br><br>Behavior score and scale: MD<br><br>Adverse events/complications: MD<br><br>Management of adverse events/complications: MD<br><br>Procedure completion: MD<br>Reason for interruption: MD	
	RCT	84<br>4-14 yo (*n*=84)<br>MD<br>MD<br>MD	Dexmedetomidine (*n*=21)<br>IN (*n*=21)<br>1 µg/kg (*n*=21)<br>Onset: MD<br>Duration: MD<br>Sleep: MD<br>Recovery: MD<br>Provider: MD<br>Setting: MD<br>N/d dental procedure (*n*=21)	Nr. of reported effective sedations: (*n*=17)<br><br>Sedation score, scale: Modified Observer's Assessment of Alertness/ Sedation scale (*n*=21)<br>Behavior score and scale: MD<br><br>Adverse events/complications: MD<br><br>Management of adverse events/complications: MD	MD<br>MD<br>MD

				Procedure completion: MD<br>Reason for interruption: MD	
			Dexmedetomidine (*n*=21)<br>IN (*n*=21)<br>1.5 μg/kg (*n*=21)<br>Onset: MD<br>Duration: MD<br>Sleep: MD<br>Recovery: MD<br>Provider:MD<br>Setting: MD<br>N/d dental procedure (*n*=21)	Nr. of reported effective sedations:(*n*=18)<br><br>Sedation score, scale: Modified Observer's Assessment of Alertness/ Sedation score, scale (*n*=21)<br><br>Behavior score and scale: MD<br><br>Adverse events/complications: MD<br><br>Management of adverse events/complications: MD<br><br>Procedure completion: MD<br>Reason for interruption: MD	
			Midazolam (*n*=21)<br>IN (*n*=21)<br>0.2 mg/kg (*n*=21)<br>Onset: MD<br>Duration: MD<br>Sleep: MD<br>Recovery: MD<br>Provider:MD<br>Setting: MD<br>N/d dental procedure (*n*=21)	Nr. of reported effective sedations: (*n*=13)<br><br>Modified Observer's Assessment of Alertness/<br>Sedation scale (*n*=21)<br><br>Behavior score and scale: MD<br><br>Adverse events/complications: MD<br><br>Management of adverse events/complications: MD<br><br>Procedure completion: MD<br>Reason for interruption: MD	
			Ketamine (*n*=21)<br>IN (*n*=21)<br>5 μg/kg (*n*=21)<br>Onset: MD	Nr. of reported effective sedations: (*n*=14)	

			Duration: MD<br>Sleep: MD<br>Recovery: MD<br>Provider:MD<br>Setting: MD<br>N/d dental procedure (*n*=21)	Modified Observer's Assessment of Alertness/<br>Sedation scale (*n*=21)<br><br>Behavior score and scale: MD<br><br>Adverse events/complications: MD<br><br>Management of adverse events/complications: MD<br><br>Procedure completion: MD<br>Reason for interruption: MD	
Lyratzopoulos 2003<br>Journal of Public Health Medicine<br>[23]<br>4<br>observational study<br>No Meta-analysis					
	Observational study	60<br>11.9 ±1.78 (*n*=60)<br>18M, 40F<br>MD<br>MD	N_2O (*n*=60)<br>Inhalation (*n*=60)<br>40/60 (N_2O/O_2%) max concentration (*n*=60)<br>Onset: MD<br>: MD<br>Duration (mean min): MD<br>Sleep: MD<br>Recovery time/score: 15.1 ± 1.54<br>Provider: Dentist<br>Setting: University dental Hospital (*n*=60)<br>Monitoring: MD	Nr. of reported effective sedations: (*n*=58)<br><br>Sedation score, Scales: N/d<br><br>Behavior scales score : VAS face scale, preoperative score 4.7 ± 1.91 (*n*=60); post-operative score 3.1 ±1.70 (*n*=60)<br><br>Adverse events/complications: immediate post-operative<br>-nausea (*n*=6); headache (*n*=6); sore mouth (*n*=2); crying (*n*=1)<br>24h post-operative	Satisfaction (child/caregiver): self-reported high satisfaction for child and caregiver

			Dental procedure: Tooth extraction (*n*=240)	-vomiting (*n*=4); headache (*n*=24); difficulties in eating/swallowing (*n*=30);<br><br>Management of adverse events/complications: MD<br><br>Procedure completion: yes (*n*=58); no (*n*=2)<br>Reason for interruption: extraction refusal (*n*=2)	
	Observational study	265<br>7.63 ± 2.45, range 3-16 yo (*n*=265)<br>143M, 122F<br>MD<br>MD	N_2O (*n*=265)<br>Mask (*n*=265)<br>40/60 (N_2O/O_2%) (*n*=265)<br>Onset: MD<br>: MD<br>Duration: MD<br>Sleep: MD<br>Recovery time/score: MD<br>Provider: MD<br>Setting: University Dental Hospital (*n*=265)<br>Dental procedure: tooth extraction (*n*= 265)	Nr. of reported effective sedations: (*n*=259); not successful (*n*=6)<br><br>Scales: N/d<br><br>Behavior score and scales: MD<br><br>Adverse events/complications: MD<br><br>Management of adverse events/complications: MD<br><br>Procedure completion: yes (*n*=221); no (*n*=44)<br>Reason for interruption: lack of cooperation (*n*=44)	Acceptability (child, caregiver): MD<br>Satisfaction (child/caregiver): self-reported high satisfaction for child (*n*=221) and caregiver (*n*=221)
	Observational study	133<br>7.8 yo, range 4-17 yo (*n*=133)<br>63M, 70F<br>MD<br>MD	N_2O (*n*=133)<br>Mask (*n*=133)<br>MD (N_2O/O_2%) (*n*=133)<br>Onset: MD<br>: MD<br>Duration (mean min): 44, range 15-115<br>Sleep: MD<br>Recovery time/score: MD<br>Provider: dentist<br>Setting: University Dental Hospital (*n*=133)	Nr. of reported effective sedations: (*n*=120)<br><br>Scales: N/d<br><br>Behavior score and scales: MD<br><br>Adverse events/complications: MD<br><br>Procedure completion: yes (*n*=120); no (*n*=13)<br>Reason for interruption: MD	Acceptability (child, caregiver): MD<br>Satisfaction (child, caregiver): 5-pointed questionnaire,<br>Child satisfied with procedure (*n*= 97/100); child reporting better satisfaction than general anesthesia (*n*=51/100) caregiver satisfied with procedure (*n*=97/100);

			Dental procedure: tooth extractions (n=127); N/d dental surgeries (n=6)		caregiver reporting better satisfaction than general anesthesia (n=79/100)
	Observational study	61<br>7.8 yo (n=61)<br>26M, 37F<br>MD<br>MD	N_2O (n=61)<br>mask (n=61)<br>MD (N_2O/O_2%) (n=61)<br>Onset: MD<br>: MD<br>Duration (range min) 22-44 (n=61)<br>Sleep: MD<br>Recovery time/score: MD<br>Provider: MD<br>Setting: Community clinic<br>Monitoring: MD<br>Dental procedure: teeth extraction (n=61)	Nr. of reported effective sedations: (n=53);<br>Scales: MD<br>Behavior score and scales: MD<br><br>Adverse events/complications: MD<br><br>Management of adverse events/complications: NA<br><br>Procedure completion: yes (n=53); no (n=8)<br>Reason for interruption: lack of cooperation (n=8)	Acceptability (child, caregiver): MD<br>Satisfaction (child/caregiver): MD
Oliveira 2023 Pesquisa Brazileira [24]<br>1 RCT<br>No meta-analysis					
	RCT	11<br>3.4 yo, (n=11)<br>MD<br>None (n=11)	Midazolam (n=11)<br>PO (n=11)<br>1.0 mg/kg (n=11)<br>Onset: MD<br>Duration (mean min): 55.2<br>Sleep: MD<br>Recovery time/score: MD<br>Provider: MD<br>Setting: dental clinic (n=11)<br>Monitoring: MD	Nr. of reported effective sedations:N=8<br><br>Scales: Houpt scale, score 5/6 (n=8); <5/6 (n=3)<br>Behavior score and scales: Houpt behavior scale; N/d scores (n=11)<br><br>Adverse events/complications: None (n=11)<br>Management of adverse events/complications: NA	MD<br>MD

			Dental procedure:<br>restorative dental Therapy (*n*=11)	Procedure completion: MD<br>Reason for interruption: NA	
Oza<br>2022<br>Cureus<br>[25]<br>2 RCT<br>Meta-analysis					
	RCT	72<br>3-6 yo (*n*=72)<br>MD<br>Weight range: 14.6 - 22.3 kgs (*n*=72)<br>None (*n*=72)	Dexmedetomidine (*n*=36)<br>IN (*n*=36)<br>1µg/kg (*n*=36)<br>Onset time (mean): 16.9 ± 4 min (*n*=36)<br>Duration: MD<br>Sleep: MD<br>Provider: MD<br>Recovery time (mean): 24.5 ± 5.1 min (*n*=36)<br>Setting: Dental clinic (in subjects with 4-point Sedation Scale ≥ 2, transfer to Operating room (N/d)<br>MD<br>N/d Dental surgery (*n*=36)	Nr. of reported effective sedations:N/d<br><br>Sedation score, scale: MD<br><br>Behavior score/scale: MD<br><br>Adverse reactions/complications: None (*n*=36)<br><br>Management of adverse events/complications: NA<br><br>Procedure completion: MD<br>Reason for interruption: NA	MD<br>MD
			Midazolam (*n*=36)<br>IN (*n*=36)<br>0.2 mg/kg (*n*=36)<br>Onset time (mean): 28.3 ± 5.8 min (*n*=36)<br>Duration: MD<br>Recovery time (mean): 24.5 ± 4.6 min<br>MD<br>Dental clinic transfer to Operating room (N/d)<br>MD<br>N/d Dental surgery (*n*=36)	Nr. of reported effective sedations:N/d<br><br>Sedation score, scale: MD<br><br>Behavior score/scale: MD<br><br>Adverse reactions/complications: None (*n*=36)<br><br>Management of adverse events/complications: NA	

				Procedure completion: MD<br>Reason for interruption: NA	
			Midazolam (*n*=37)<br>PO (*n*=37)<br>0.5mg/kg (*n*=37)<br>Onset: MD<br>Sleep: MD<br>Duration: MD<br>Recovery time (mean): 108.62± 58.7 min<br>N/d Dental surgery (*n*=37)	Nr. of reported effective sedations:N/d<br><br>Sedation score, scale: MD<br><br>Behavior score/scale: MD<br><br>Adverse reactions/complications: None (*n*=36)<br><br>Management of adverse events/complications: NA<br><br>Procedure completion: MD<br>Reason for interruption: NAMD	
	RCT	84<br>4-14 yo (*n*=84)<br>43 M, 41 F<br>Weight ranges: 9-27 kgs (*n*=84)<br>None (*n*=84); ASA I physical status (*n*=84)	Dexmedetomidine (*n*=21)<br>IN (*n*=21)<br>1µg/kg (*n*=21)<br>Onset (min): 18.24 ± 2.00 (*n*=21)<br>: MD<br>Duration: MD<br>Recovery time (min): 59.81 ±5.89 (*n*=21)<br>Provider: Dentist and anesthesiologist supervision (*n*=21)<br>Setting: MD<br>Monitoring: Oxygen saturation (*n*=21); respiratory rate (*n*=21);	Nr. of reported effective sedations:MD<br>5-point sedation scale level (modified AAPD scale), N/d<br><br>Behavior score and scales:<br>5-point behavior response to treatment N/d<br>Management of adverse events/complications: MD<br><br>Procedure completion: MD<br>Reason for interruption: NA	MD<br>MD<br>MD

			blood pressure (*n*=21);<br>pulse rate (*n*=21)<br>Dental procedure: teeth extraction (*n*=21)		
			Dexmedetomidine (*n*=21)<br>IN (*n*=21)<br>1.5µg/kg (*n*=21)<br>Onset (min): 18.10 ± 2.00 (*n*=21)<br>Duration: MD<br>Recovery time (min): 62.24±7.17 (*n*=21)<br>Provider: Dentist and anesthesiologist supervision (*n*=21)<br>Setting: MD<br>Monitoring: O_2 saturation (*n*=21); respiratory rate (*n*=21); DBP (*n*=21); pulse rate (*n*=21)<br>Dental procedure: teeth extraction (*n*=21)	Nr. of reported effective sedations:MD<br>5-point sedation scale level (modified AAPD scale), N/d<br><br>Behavior score and scales:<br>5-point behavior response to treatment N/d<br>Management of adverse events/complications: MD<br><br>Procedure completion: MD<br>Reason for interruption: NA	
			Midazolam (*n*=21)<br>IN (*n*=21)<br>0.2 mg/kg (*n*=21)<br>Onset (min): 10.43±1.83 (*n*=21)<br>Duration: MD<br>Recovery time (min): 40.71±2.45 (*n*=21)<br>Provider: Dentist and anesthesiologist supervision (*n*=21)<br>Setting: MD<br>Monitoring: O_2 saturation (*n*=21); respiratory rate *n*=21); DBP (*n*=21); pulse rate (beats/min, mean) (*n*=21)	Nr. of reported effective sedations:MD<br>5-point sedation scale level (modified AAPD scale), N/d<br><br>Behavior score and scales:<br>5-point behavior response to treatment N/d<br>Management of adverse events/complications: MD<br><br>Procedure completion: MD<br>Reason for interruption: NA	

			Dental procedure: teeth extraction (*n*=21)		
			Ketamine (*n*=21)<br>IN (*n*=21)<br>5 mg/kg (*n*=21)<br>Onset (min): 11.57±2.18 (*n*=21)<br>Duration: MD<br>Recovery time (min): 44.19±5.24 (*n*=21)<br>Provider: Dentist and anesthesiologist supervision (*n*=21)<br>Setting: MD<br>Monitoring: O_2 saturation (*n*=21); respiratory rate (*n*=21); DBP (*n*=21); pulse rate (*n*=21)<br>Dental procedure: teeth extraction (*n*=21)	Nr. of reported effective sedations:MD<br>5-point sedation scale level (modified AAPD scale), N/d<br><br>Behavior score and scales:<br>5-point behavior response to treatment N/d<br>Adverse reactions/complications: MD<br>Management of adverse events/complications: MD<br><br>Procedure completion: MD<br>Reason for interruption: NA	

Poonai<br>2017<br>Plos one<br>[26]<br>4 RCT<br>No Meta-analysis	RCT	30<br>1.5-6 yo (*n*=30)<br>MD<br>MD<br>MD	Ketamine (*n*=10)<br>IN (*n*=10)<br>3 mg/kg (*n*=10)<br>Onset: MD<br>Duration: MD<br>Sleep: MD<br>Recovery: MD<br>Provider: MD<br>Setting: MD<br>Monitoring: oxygen saturation, N/d levels<br>N/d dental procedure (*n*=10)	Nr. of reported effective sedations:MD<br><br>Sedation score, scale: (10-item scale): mean sedation level 4 (range 3-6) (*n*=10)<br><br>Behavior score, scale: MD<br><br>Adverse events/complications: Transient oxygen desaturation, spontaneously resolving (*n*=2)<br>Management of adverse events/complications: None (*n*=2)<br><br>Procedure completion: MD<br>MD	MD<br>MD<br>MD
			Sufentanil (*n*=10)<br>IN (*n*=10)<br>1 μg/kg (*n*=5) and 1.5 μg/kg (*n*=5)<br>Onset: MD<br>Duration: MD<br>Sleep: MD<br>Recovery: MD<br>Provider: MD<br>Setting: MD<br>Monitoring: MD<br>N/d dental procedure (*n*=10)	Nr. of reported effective sedations:MD<br><br>Depth of sedation (10-item scale): mean sedation level 7 (range 2-9) in 1.5 μg/kg; 4 (range 3-5) in 1 μg/kg (*n*=10)<br><br>Behavior score, scale: MD<br><br>Adverse events/complications: Transient oxygen desaturation, spontaneously resolving (*n*=2)<br>Management of adverse events/complications: None (*n*=2)<br><br>Procedure completion: MD<br>MD	

			Midazolam (*n*=10)<br>IN (*n*=10)<br>0.4 mg/kg<br>Onset: MD<br>Duration: MD<br>Sleep: MD<br>Recovery: MD<br>Provider: MD<br>Setting: MD<br>Monitoring: MD<br>N/d dental procedure (*n*=10)	Nr. of reported effective sedations:MD<br><br>Sedation score, scale: Depth of sedation (10-item scale): mean sedation level 4 (range 2-5) (*n*=10)<br><br>Behavior score, scale: MD<br><br>Adverse events/complications: Transient oxygen desaturation, spontaneously resolving (*n*=2)<br>Management of adverse events/complications: None (*n*=2)<br><br>Procedure completion: MD<br>Reason for interruption: MD	
	RCT	45<br>4.6 yo, range 2-6 yo<br>MD<br>MD<br>MD	Ketamine (*n*=45)<br>IN (*n*=45)<br>6 mg/kg (*n*=45)<br>Mean onset: from 7 to 69 min. range 3.6 to 11.6 min (*n*=45)<br>Onset: MD<br>Duration: MD<br>Sleep: MD<br>Recovery: MD<br>Provider: MD<br>Setting: MD<br>Monitoring: MD<br>N/d dental procedure (*n*=45)	Nr. of reported effective sedations:*n*=42<br><br>Sedation scale, score: 5- item sedation scale, N/d<br><br>Behavior score, scale: MD<br><br>Adverse events/complications: Vomiting (N/d)<br><br>Management of adverse events/complications: MD<br><br>Procedure completion: MD<br>Reason for interruption: MD	Child acceptance: IN ketamine “well tolerated” (*n*=45)<br>MD<br>MD
			Midazolam (*n*=45)<br>IN (*n*=45)<br>0.3 mg/kg (*n*=45)<br>Mean onset: from 7 to 69 min. range 3.6 to 11.6 min (*n*=45)<br>Onset: MD<br>Duration: MD	Nr. of reported effective sedations:*n*=42<br><br>Sedation scale, score: 5- item sedation scale, N/d<br><br>Behavior score, scale: MD	

			Sleep: MD<br>Recovery: MD<br>Provider: MD<br>Setting: MD<br>Monitoring: MD<br>N/d dental procedure (*n*=45)	Adverse events/complications: MD<br><br>Management of adverse events/complications: MD<br><br>Procedure completion: MD<br>Reason for interruption: MD	
	RCT	34<br>4.4 yo range 2-6 yo<br>MD<br>MD<br>MD	Ketamine (*n*=34)<br>IN (*n*=34)<br>6 mg/kg (*n*=34)<br>Mean onset: range 3.6 to 11.6 min (*n*=34)<br>Onset: MD<br>Duration: MD<br>Sleep: MD<br>Recovery: MD<br>Provider: MD<br>Setting: MD<br>Monitoring: MD<br>N/d dental procedure (*n*=34)	Nr. of reported effective sedations:*n*=32<br><br>Sedation scale, score: 5- item sedation scale, N/d<br><br>Behavior score, scale: MD<br><br>Adverse events/complications: MD<br><br>Management of adverse events/complications: MD<br><br>Procedure completion: MD<br>Reason for interruption: MD	
			Ketamine (*n*=34)<br>IN (*n*=34)<br>6 mg/kg (*n*=34)<br>Onset: MD<br>Duration: MD<br>Sleep: MD<br>Recovery: MD<br>Provider: MD<br>Setting: MD<br>Monitoring: MD<br>N/d dental procedure (*n*=34)	Nr. of reported effective sedations: (*n*=29)<br>Sedation scale, score: 5- item sedation scale, N/d<br><br>Behavior score, scale: MD<br><br>Adverse events/complications: MD<br><br>Management of adverse events/complications: MD<br><br>Procedure completion: MD<br>Reason for interruption: MD	

	RCT	84<br>7.3 yo, range 4-14 yo<br>MD<br>MD<br>MD	Ketamine (*n*=21)<br>IN (*n*=21)<br>5 mg/kg (*n*=21)<br>Mean onset: from 7 to 69 min. range 3.6 to 11.6 min (*n*=84)<br>Duration: MD<br>Sleep: MD<br>Recovery: MD<br>Provider: MD<br>Setting: MD<br>Monitoring: MD<br>N/d dental procedure (*n*=21)	Nr. of reported effective sedations:(*n*=14)<br><br>Sedation scale, score: 5- item sedation scale, N/d<br><br>Behavior score, scale: MD<br><br>Adverse events/complications: MD<br><br>Management of adverse events/complications: MD<br><br>Procedure completion: MD<br>Reason for interruption: MD	MD<br>MD<br>MD
			Midazolam (*n*=21)<br>IN (*n*=21)<br>0.2 mg/kg (*n*=21)<br>Onset: MD<br>Duration: MD<br>Sleep: MD<br>Recovery: MD<br>Provider: MD<br>Setting: MD<br>Monitoring: MD<br>N/d dental procedure (*n*=21)	Nr. of reported effective sedations:(*n*=13);<br>Sedation scale, score: 5- item sedation scale, N/d<br><br>Behavior score, scale: MD<br><br>Adverse events/complications: MD<br><br>Management of adverse events/complications: MD<br><br>Procedure completion: MD<br>Reason for interruption: MD	
			Dexmedetomidine (*n*=21)<br>IN (*n*=21)<br>1 μg/kg (*n*=21)<br>Onset: MD<br>Duration: MD<br>Sleep: MD<br>Recovery: MD<br>Provider: MD	Nr. of reported effective sedations:(*n*=17);<br><br>Sedation scale, score: 5- item sedation scale, N/d<br><br>Behavior score, scale: MD	

			Setting: MD<br>Monitoring: MD<br>N/d dental procedure<br>(*n*=21)	Adverse events/complications: MD<br><br>Management of adverse events/complications: MD<br><br>Procedure completion: MD<br>Reason for interruption: MD	
			Dexmedetomidine (*n*=21)<br>IN (*n*=21)<br>1.5 μg/kg (*n*=21)<br>Onset: MD<br>Duration: MD<br>Sleep: MD<br>Recovery: MD<br>Provider: MD<br>Setting: MD<br>Monitoring: MD<br>N/d dental procedure<br>(*n*=21)	Nr. of reported effective sedations:(*n*=18)<br><br>Sedation scale, score: 5- item sedation scale, N/d<br><br>Behavior score, scale: MD<br><br>Adverse events/complications: MD<br><br>Management of adverse events/complications: MD<br><br>Procedure completion: MD<br>Reason for interruption: MD	

Poonai<br>2020<br>Pediatrics<br>[27]<br>3 RCT<br>No-Meta analysis	RCT	44<br>4-9 yo<br>MD<br>MD<br>MD	Dexmedetomidine (*n*=11)<br>IN (*n*=11)<br>2.5 µg/kg (*n*=11)<br>7-20.6 min (*n*=11)<br>Onset: MD<br>Duration: MD<br>Sleep: MD<br>Recovery: MD<br>Provider: MD<br>Setting: MD<br>Monitoring: MD<br>N/d dental procedure (*n*=11)	Successful 82%, (*n*=9); not successful (*n*=2)<br><br>5-point scale: "safe and successful" (*n*=9)<br><br>Behavior score, scale: MD<br><br>Adverse events/complications: MD<br><br>Management of adverse events/complications: MD<br><br>Procedure completion: MD<br>Reason for interruption: MD	Child acceptance of IN dexmedetomidine: "fair to excellent" (*n*=16) (16/22, 72.7%)<br>MD<br>MD
			Dexmedetomidine (*n*=11)<br>IN (*n*=11)<br>2 µg/kg (*n*=11)<br>8.8–25 min (*n*=11)<br>Onset: MD<br>Duration: MD<br>Sleep: MD<br>Recovery: MD<br>Provider: MD<br>Setting: MD<br>Monitoring: MD<br>N/d dental procedure (*n*=11)	Successful 27%, (*n*=3); not successful (*n*=8)<br><br>Sedation score, scale: 5-point scale: score 5"safe and successful" (*n*=3)<br><br>Behavior score, scale: MD<br><br>Adverse events/complications: MD<br><br>Management of adverse events/complications: MD<br><br>Procedure completion: MD<br>Reason for interruption: MD	
			Dexmedetomidine (*n*=11)<br>PO (*n*=11)<br>4 µg/kg (*n*=11)<br>Onset: MD<br>Duration: MD<br>Sleep: MD<br>Recovery: MD	Successful 0% (*n*=0); not successful 100% (*n*=11)<br><br>Sedation score, scale: MD<br><br>Behavior score, scale: MD	

			Provider: MD<br>Setting: MD<br>Monitoring: MD<br>N/d dental procedure (*n*=11)	Adverse events/complications: MD<br><br>Management of adverse events/complications: MD<br><br>Procedure completion: MD<br>Reason for interruption: MD	
			Dexmedetomidine (*n*=11)<br>PO (*n*=11)<br>5 µg/kg (*n*=11)<br>Onset: MD<br>Duration: MD<br>Sleep: MD<br>Recovery: MD<br>Provider: MD<br>Setting: MD<br>Monitoring: MD<br>N/d dental procedure (*n*=11)	Successful 0% (*n*=0); not successful 100% (*n*=11)<br><br>Sedation score, scale: MD<br><br>Behavior score, scale: MD<br><br>Adverse events/complications: MD<br><br>Management of adverse events/complications: MD<br><br>Procedure completion: MD<br>Reason for interruption: MD	
	RCT	84<br>4-14 yo<br>MD<br>MD<br>MD	Dexmedetomidine (*n*=21)<br>IN (*n*=21)<br>1.5 µg/kg (*n*=21)<br>Onset: MD<br>Duration: MD<br>Sleep: MD<br>Recovery: MD<br>Provider: MD<br>Setting: MD<br>Monitoring: MD<br>N/d dental procedure (*n*=21)	Nr. of reported effective sedations: (*n*=18)<br><br>Sedation score, scale: 5-point scale: score 4 or 5 "satisfactory sedation" (*n*=18)<br><br>Behavior score, scale: FLACC score: 3.8 ± 0.8 (*n*=21)<br><br>Adverse events/complications: None (*n*=21)<br><br>Management of adverse events/complications: NA	Child acceptance:<br>IN Dexmedetomidine: "well accepted" (*n*=42);<br>IN midazolam: "well accepted" (*n*=21)<br>IN ketamine "well accepted" (*n*=21)<br><br>MD<br>MD

				Procedure completion: MD<br>Reason for interruption: MD	
			Dexmedetomidine (*n*=21)<br>IN (*n*=21)<br>1 μg/kg (*n*=21)<br>Onset: MD<br>Duration: MD<br>Sleep: MD<br>Recovery: MD<br>Provider: MD<br>Setting: MD<br>Monitoring: MD<br>N/d dental procedure (*n*=21)	Nr. of reported effective sedations: (*n*=17)<br><br>Sedation score, scale: 5-point scale: score 4 or 5 "satisfactory sedation" (*n*=17)<br><br>FLACC score: 3.7 ± 0.9 (*n*=21)<br><br>Adverse events/complications: None (*n*=21)<br><br>Management of adverse events/complications: NA<br><br>Procedure completion: MD<br>Reason for interruption: MD	
			Midazolam (*n*=21)<br>IN (*n*=21)<br>0.2 mg/kg (*n*=21)<br>Onset: MD<br>Duration: MD<br>Sleep: MD	Nr. of reported effective sedations: (*n*=13)<br><br>Sedation score, scale: 5-point scale: score 4 or 5 "satisfactory sedation" (*n*=13)	

			Recovery: MD<br>Provider: MD<br>Setting: MD<br>Monitoring: MD<br>N/d dental procedure (*n*=21)	Behavior score, scale: FLACC score: 5.6 ± 1.1 (*n*=21)<br><br>Adverse events/complications: None (*n*=21)<br><br>Management of adverse events/complications: NA<br><br>Procedure completion: MD	
			Ketamine (*n*=21)<br>IN (*n*=21)<br>5 mg/kg (*n*=21)<br>Onset: MD<br>Duration: MD<br>Sleep: MD<br>Recovery: MD<br>Provider: MD<br>Setting: MD<br>Monitoring: MD<br>N/d dental procedure (*n*=21	Nr. of reported effective sedations:(*n*=14)<br>Sedation score, scale : 5-point scale: score 4 or 5 "satisfactory sedation" (*n*=14)<br><br>Behavior score, scale: FLACC score: 3.5 ± 0.7 (*n*=21)<br><br>Adverse events/complications: None (*n*=21)<br><br>Management of adverse events/complications: NA<br><br>Procedure completion: MD	
Salerno 2023 Environ. Res. Public Health [31]<br>1 RCT, 1 Observational study<br>No Meta-analysis					

	Observational study	472<br>6.6 yo, range 4-17 yo<br>MD<br>MD<br>Intellectual disability	N_2O (*n*=472)<br>Mask (*n*=472)<br>Various concentration (N_2O/O_2) (*n*=472)<br>Onset: MD<br>Duration: MD<br>Recovery:<br>Provider: Dentist<br>Setting: MD<br>Monitoring: N/d vital signs (*n*=472)<br>PO examination, PO hygiene, restorative dental therapy, N/d PO surgery (*n*=472)	Nr. of reported effective sedations: (*n*=354)<br><br>Sedation score/scale:MD<br>Behavior score/scale: Modified Venham Scale (behavior) N/d scores (*n*=472)<br><br>Adverse reactions/complications: MD<br>Management of adverse events/complications: MD<br><br>Procedure completion: MD<br>Reason for interruption: NA	
	RCT	31<br>10 yo, range 3-18<br>16M/15F<br>MD<br>Intellectual disability	Midazolam (*n*=31)<br>PO (*n*=31)<br>0.3mg/kg (*n*=16) 0.5 mg/kg (*n*=15)<br>Onset: MD<br>Duration: MD<br>Sleep: MD<br>Recovery: MD<br>Dentist and anesthesiologist<br>Setting: MD<br>Monitoring: N/d vital signs<br>N/d dental procedures	Nr. of reported effective sedations: (*n*=12 in 0.3 mg/kg), (*n*=9 in 0.5 mg/kg)<br><br>Sedation score, scale: MD<br>Behavior score, scale: Modified Frankl Scale: score 3 (12 of participants of 0.3 mg/kg; 9 of 0.5 mg/kg)<br><br>Adverse reactions/complications: MD<br>Management of adverse events/complications: MD<br><br>Procedure completion: MD<br>Reason for interruption: NA	MD<br>MD<br>MD

Preethy 2021					

J Contemp Clin Med. [28]<br>9 RCT<br>No Meta-analysis					
	RCT	30<br>2-8 yo<br>MD<br>MD<br>MD	Midazolam (*n*=30)<br>Buccal via aerosol mouth spray (*n*=30)<br>0.25 mg/kg<br>Onset: MD<br>Duration: MD<br>Sleep: MD<br>Recovery: MD<br>Dentist and anesthesiologist<br>Setting: MD<br>Monitoring: MD<br>N/d dental treatment (*n*=30)	Nr. of reported effective sedations:(*n*=20)<br>Sedation score, scale: MD<br><br>Behavior score, scale: Houpt behavior rating scale: (66.7%) “acceptable behavior, score 3-4) (*n*=20)<br><br>Adverse reactions/complications: MD<br>Management of adverse events/complications: MD<br><br>Procedure completion: Yes (*n*=20)<br>Reason for interruption: N/d	MD<br>MD<br>MD
			Midazolam (*n*=30)<br>IN via spray (*n*=30)<br>0.25 mg/kg<br>Onset: MD<br>Duration: MD<br>Sleep: MD<br>Recovery: MD<br>Dentist and anesthesiologist<br>Setting: MD<br>Monitoring: MD<br>N/d dental treatment (*n*=30)	Nr. of reported effective sedations: (*n*=17)<br>Sedation score, scale: MD<br><br>Behavior score, scale: Houpt behavior rating scale: (60%) “acceptable behavior”, score 3-4) (*n*=20)<br><br>Adverse reactions/complications: MD<br>Management of adverse events/complications: MD<br><br>Procedure completion: Yes (*n*=17)<br>Reason for interruption: N/d	
	RCT	100<br>1.5-6 yo<br>MD<br>MD<br>MD	Midazolam (*n*=50)<br>IN (*n*=50)<br>0.2 mg/kg (*n*=50)<br>Onset: MD<br>Duration: MD	Nr. of reported effective sedations:(*n*=33)<br><br>Sedation score, scale: N/d scale	MD<br>MD<br>MD

			Sleep: MD<br>Recovery: MD<br>Provider: Dentist and anesthesiologist<br>Setting: MD<br>Monitoring: MD<br>N/d dental treatment (*n*=50)	Behavior score, scale: Global Behavior Rating Scale: "satisfactory rate" (*n*=33)<br><br>Adverse reactions/complications: MD<br>Management of adverse events/complications: MD<br>MD<br><br>Procedure completion: Yes (*n*=33)<br>Reason for interruption: MD	
	RCT	40<br>2-7 yo (*n*=40)<br>MD<br>MD<br>MD	Midazolam (*n*=20)<br>PO (*n*=20)<br>0.7 mg/kg (*n*=20)<br>Onset: MD<br>Duration: MD<br>Sleep: MD<br>Recovery: 30-35 min<br>Provider: MD<br>Setting: MD<br>Monitoring: MD<br>N/d dental treatment (*n*=20)	Effecrive sedations: (*n*=20)<br><br>Sedation score, scale: N/d<br><br>Behavior score, scale: Modified- Houpt Behavior Rating Scale: N/d scores<br><br>Adverse reactions/complications: MD<br>Management of adverse events/complications: MD<br><br>Procedure completion: Yes (*n*=20)<br>Reason for interruption: NA	MD<br>MD<br>MD
			Midazolam (*n*=20)<br>IN (*n*=20)<br>0.3 mg/kg (*n*=20)<br>Onset: MD<br>Duration: MD<br>20-35 min<br>Provider: MD<br>Setting: MD<br>Monitoring: N/d<br>N/d dental treatment (*n*=20)	Nr. of reported effective sedations: (*n*=20)<br><br>Sedation score, scale: N/d<br><br>Behavior score, scale: Modified- Houpt Behavior Rating Scale: N/d scores<br><br>Adverse reactions/complications: MD<br>Management of adverse events/complications: MD<br><br>Procedure completion: Yes (*n*=20)<br>Reason for interruption: NA	
	RCT	240<br>4-6 yo (*n*=240)	N_2O (*n*=60)<br>Ihnalation (*n*=60)	Nr. of reported effective sedations: (*n*= 33)	MD<br>MD

		MD<br>MD<br>MD	50/50 (N_2O/O_2 %) (*n*=60)<br>Onset: MD<br>Duration: MD<br>Sleep: MD<br>Recovery: MD<br>Provider: MD<br>Setting: MD<br>Monitoring: MD<br>N/d dental treatment (*n*=60)	Sedation score, scale: Bispectral Index System (*n*=60), N/d score<br><br>Behavior score, scale: Modified scale to classify behavior/response to treatment/sedation:<br>Excellent: 23% (*n*=14)<br>Adequate: 32% (*n*=19)<br><br>Adverse reactions/complications: MD<br>Management of adverse events/complications: MD<br><br>Procedure completion Yes (*n*=33)<br>Reason for interruption: MD	MD
			Midazolam (*n*=60)<br>IN (*n*=60)<br>0.20 mg/kg (*n*=60)<br>Onset: MD<br>Duration: MD<br>Sleep: MD<br>Recovery: MD<br>Provider: MD<br>Setting: MD<br>Monitoring: MD<br>N/d dental treatment (*n*=60)	Nr. of reported effective sedations: (*n*= 52)<br><br>Sedation score, scale: Bispectral Index System: at 15 min > 90 (*n*=60)<br><br>Behavior score, scale: Modified scale to classify behavior/response to treatment/sedation:<br>Excellent: 72% (*n*=43)<br>Adequate: 15% (*n*=9)<br><br>Adverse reactions/complications: MD<br>Management of adverse events/complications: MD<br><br>Procedure completion: Yes (*n*=52)<br>Reason for interruption: MD	
	RCT	40<br>3-7 yo (*n*=20)<br>MD<br>MD<br>MD	Midazolam (*n*=20)<br>SL (*n*=20)<br>0.3 mg/kg (*n*=20)<br>Onset: MD<br>Duration: MD	Effective sedation: MD<br><br>Sedation score,scale: MD	MD<br>MD<br>MD

			Sleep: MD<br>Recovery: MD<br>Provider: MD<br>Setting: MD<br>Monitoring: MD<br>N/d dental treatment (*n*=20)	Behavior score, scale: Modified Houpt's Behavior Rating Scale:<br>Fair 10% (*n*=2)<br>Good 5% (*n*=1)<br>Excellent 85% (*n*=17)<br><br>Adverse reactions/complications: MD<br>Management of adverse events/complications: MD<br><br>Procedure completion: MD<br>Reason for interruption: MD	
			Midazolam (*n*=20)<br>IN (*n*=20)<br>0.3 mg/kg (*n*=20)<br>Onset: MD<br>Duration: MD<br>Sleep: MD<br>Recovery: MD<br>Provider: MD<br>Setting: MD<br>Monitoring: MD<br>N/d dental treatment (*n*=20)	Effective sedation: MD<br><br>Sedation score, scale: MD<br><br>Behavior score, scale: Modified Houpt's Behavior Rating Scale:<br>Fair 5% (*n*=1)<br>Good 10% (*n*=2)<br>Excellent 85% (*n*=17)<br><br>Adverse reactions/complications: MD<br>Management of adverse events/complications: MD<br><br>Procedure completion: MD<br>Reason for interruption: MD	
	RCT	40<br>3-7 yo<br>MD<br>MD<br>MD	Midazolam (*n*=20)<br>SL (*n*=20)<br>0.2 mg/kg (*n*=20)<br>Onset: MD<br>Duration: MD<br>Sleep: MD<br>Recovery: MD<br>Provider: MD<br>Setting: MD<br>Monitoring: MD	Nr. of reported effective sedations:MD<br><br>Sedation score, scale: MD<br><br>Behavior score, scale: Venham's Clinical Anxiety Scale (*n*=20); N/d (*n*=20)<br><br>Adverse reactions/complications: MD<br>Management of adverse events/complications: MD	N/d acceptability scales and scores (*n*=20)<br>MD<br>MD

			N/d dental procedure<br>(*n*=20)	Procedure completion: MD<br>Reason for interruption: MD	
			Midazolam (*n*=20)<br>IN (*n*=20)<br>0.2 mg/kg (*n*=20)<br>Onset: MD<br>Duration: MD<br>Sleep: MD<br>Recovery: MD<br>Provider: MD<br>Setting: MD<br>Monitoring: MD<br>N/d dental procedure<br>(*n*=20)	Nr. of reported effective sedations:MD<br><br>Sedation score, scale: MD<br><br>Behavior score, scale: Venham's Clinical Anxiety Scale (*n*=20); N/d (*n*=20)<br><br>Adverse reactions/complications: MD<br>Management of adverse events/complications: MD<br><br>Procedure completion: MD<br>Reason for interruption: MD	
	RCT	20<br>3-7 yo<br>MD<br>MD<br>MD	Midazolam (*n*=10)<br>SL (*n*=10)<br>0.2 mg/kg (*n*=10)<br>Onset: MD<br>Duration: MD<br>Sleep: MD<br>Recovery: MD<br>Provider: MD<br>Setting: MD<br>Monitoring: MD<br>N/d dental procedure<br>(*n*=10)	Nr. of reported effective sedations:MD<br><br>Sedation score, scale: MD<br><br>Behavior score, scale: Venham's Clinical Anxiety Scale (*n*=10); N/d (*n*=10)<br><br>Adverse reactions/complications: MD<br>Management of adverse events/complications: MD<br><br>Procedure completion: MD<br>Reason for interruption: MD	MD<br>MD<br>MD
			Midazolam (*n*=10)<br>IN spray via MAD (*n*=10)<br>0.2 mg/kg (*n*=10)<br>Onset: MD<br>Duration: MD<br>Sleep: MD<br>Recovery: MD<br>Provider: MD<br>Setting: MD<br>Monitoring: MD	Nr. of reported effective sedations:MD<br><br>Sedation score, scale: MD<br><br>Behavior score, scale: Venham's Clinical Anxiety Scale (*n*=10); N/d (*n*=10)<br><br>Adverse reactions/complications: MD<br>Management of adverse events/complications: MD	

			N/d dental procedure<br>(*n*=10)	Procedure completion: MD<br>Reason for interruption: MD	
	RCT	40<br>2-5 yo<br>MD<br>MD<br>MD	Midazolam (*n*=20)<br>IM (*n*=20)<br>0.2 mg/kg (*n*=20)<br>Onset: MD<br>Duration: MD<br>Sleep: MD<br>Recovery: MD<br>Provider: MD<br>Setting: MD<br>Monitoring: MD<br>N/d dental procedure<br>(*n*=20)	Nr. of reported effective sedations:MD<br><br>Sedation score, scale: MD<br><br>Behavior score, scale: Houpt scale; Fukuta *et al*. Modified Behavior Rating Scale (N/d scores) (*n*=20)<br><br>Adverse reactions/complications: MD<br>Management of adverse events/complications: MD<br><br>Procedure completion: MD<br>Reason for interruption: MD	MD<br>MD<br>MD
			Midazolam (*n*=20)<br>IN (*n*=20)<br>0.2 mg/kg (*n*=20)<br>Onset: MD<br>Duration: MD<br>Sleep: MD<br>Recovery: MD<br>Provider: MD<br>Setting: MD<br>Monitoring: MD<br>N/d dental procedure<br>(*n*=20)	Nr. of reported effective sedations:MD<br><br>Sedation score, scale: MD<br><br>Behavior score, scale: Houpt scale; Fukuta *et al*. Modified Behavior Rating Scale (N/d scores) (*n*=20)<br><br>Adverse reactions/complications: MD<br>Management of adverse events/complications: MD<br><br>Procedure completion: MD<br>Reason for interruption: MD	
	RCT	25<br>3-6 yo (*n*=25)<br>MD<br>MD<br>MD	Midazolam (*n*=25)<br>Buccal route (*n*=25)<br>N/d onset (*n*=25)<br>Onset: MD<br>Duration: MD<br>Sleep: MD<br>Recovery: MD<br>Provider: MD	Nr. of reported effective sedations:(*n*=22)<br><br>Sedation score, scale: MD<br>Behavior score, scale: Houpt's Behavior rating scale:	N/d acceptability scales<br>MD<br>MD

			Setting: MD<br>Monitoring: MD<br>N/d dental procedure (*n*=25)	Excellent, 12% (*n*=3); Very good, 32% (*n*=8); Good, 24% (*n*=6); Fair, 20% (*n*=5); Poor ,12% (*n*=3)<br><br>Adverse reactions/complications: MD<br>Management of adverse events/complications: MD<br><br>Procedure completion: Yes (*n*=22); no (3)<br>Reason for interruption: MD	
			Midazolam (*n*=25)<br>IN(*n*=25)<br>N/d onset (*n*=25)<br>Onset: MD<br>Duration: MD<br>Sleep: MD<br>Recovery: MD<br>Provider: MD<br>Setting: MD<br>Monitoring: MD<br>N/d dental procedure (*n*=25)	Nr. of reported effective sedations:(*n*=24)<br><br>Sedation score, scale: MD<br><br>Behavior score, scale: Houpt's Behavior rating scale: Excellent, 16% (*n*=4); Very good, 52% (*n*=13); Good, 20% (*n*=5); Fair, 4% (*n*=1); Poor,8% (*n*=2)<br><br>Adverse reactions/complications: MD<br>Management of adverse events/complications: MD<br><br>Procedure completion: Yes (*n*=24), no (*n*=1)<br>Reason for interruption: MD	
Qiu<br>2019<br>Medicine<br>[29]<br>3 RCT<br>Meta-analysis					
	RCT	14<br>range 3-9 yo (*n*=14)<br>MD<br>MD<br>None (*n*=14)	Dexmedetomidine (*n*=14)<br>IN (*n*=14)<br>1 μg/kg (*n*=14)<br>Onset: MD<br>: MD	Nr. of reported effective sedations:(*n*=9)<br><br>Sedation score,scales: MOASS scale<br>Baseline:<br>3.61 ±1.71 (*n*=14)	MD<br>MD<br>MD

			Duration: MD<br>Sleep: yes (*n*=14)<br>Recovery time/score: MD<br>Provider: pediatric dentist<br>Setting: operatory room (*n*=14)<br>Monitoring: blood pressure, heart rate, oxygen saturation (*n*=14)<br>Dental procedure: dental surgery (*n*=14)	Behavior score and scales: MD<br><br>Adverse events/complications: none (*n*=14)<br><br>Management of adverse events/complications: MD<br><br>Procedure completion: MD<br>Reason for interruption: MD	
	RCT	42<br>MD<br>MD<br>MD<br>MD	Dexmedetomidine (*n*=21)<br>IN (*n*=21)<br>1.5 µg/kg (*n*=21)<br>Onset (mean min): 18.24±2.00 (*n*=21)<br>Recovery time (mean min): 62.24±7.17 (*n*=21)<br>Provider: pediatric dentist<br>Setting: MD<br>Monitoring: Oxygen saturation; respiratory rate, heart rate, blood pressure (*n*=21)<br>Dental procedure: Dental surgery (*n*=21)<br>Sedation type:	Nr. of reported effective sedations: MD<br>Success rate: MD<br><br>Behavior score and scales: MD<br>Adverse events/complications: MD<br>Management of adverse events/complications: NA<br><br>Procedure completion: MD<br>Reason for interruption: NA	MD<br>MD<br>MD
			Ketamine (*n*=21)<br>IN (*n*=21)<br>5 mg/kg (*n*=21)<br>Onset (mean min): 11.57±2.18 (*n*=21)<br>Recovery time (mean min): 44.19±5.24 (*n*=21)<br>Provider: pediatric dentist<br>Setting: MD<br>Monitoring: Oxygen saturation; respiratory	Nr. of reported effective sedations: MD<br>Success rate: MD<br><br>Behavior score and scales: MD<br>Adverse events/complications: MD<br>Management of adverse events/complications: MD<br><br>Procedure completion: MD<br>Reason for interruption: NA	

			rate, heart rate, blood pressure (n=21)Dental procedure: N/d dental surgery (n=21)<br>Sedation type:		
	RCT	56<br>MD<br>13M/13F<br>MD	Dexmedetomidine (n=28)<br>PO (n=28)<br>5mg/kg (n=28)<br>Onset (mean min): 23.61 ± 4.12 (n=28)<br>: MD<br>Duration: MD<br>Sleep: MD<br>Recovery time/score: 108.64 ± 15.15 (n=28)<br>Provider: MD<br>Setting: MD<br>Monitoring: SpO_2 (mean %) 98.65 ± 0.24 (n=28);<br>Pulse rate; blood pressure respiratory rate<br>Dental Procedure: dental surgery (n=28)<br>Sedation type: moderate sedation (n=28)	Nr. of reported effective sedations:MD<br>Scales: 5-point AAPD, N/d (n=13)<br><br>Behavior score and scales: MD<br><br>Adverse events/complications: MD<br><br>Management of adverse events/complications: MD<br><br>Procedure completion: MD<br>Reason for interruption: MD	MD<br>MD<br>MD
			Ketamine (n=28)<br>PO (n=28)<br>8 mg/kg (n=28)<br>Onset (mean min): 21.11 ± 4.10 (n=28)<br>Duration: MD<br>Sleep: MD<br>Recovery time/score: 108.64 ± 15.15 (n=28)<br>Provider: MD<br>Setting: MD<br>Monitoring: oxygen saturation, heart rate	Nr. of reported effective sedations:MD<br>Sedation score, scales: 5-point AAPD, N/d<br><br>Behavior score and scales: MD<br><br>Adverse events/complications: MD<br><br>Management of adverse events/complications: MD<br><br>Procedure completion: MD<br>Reason for interruption: MD	

			blood pressure;<br>respiratory rate (*n*=28)<br>Dental Procedure: dental surgery (*n*=28)<br>Sedation type: moderate sedation (*n*=28)		
Rathi<br>2022<br>Int J Clin Pediatr Dent<br>[30]<br>4 RCT<br>No Meta-analysis					
	RCT	60<br>2-9 yo (*n*=60)<br>MD<br>MD<br>None (*n*=60)	Midazolam (*n*=60)<br>PO (*n*=60)<br>0.5 mg/kg (*n*=60)<br>Onset: N/D<br>Duration: MD<br>Sleep: MD<br>Recovery time/score: N/D<br>Provider: MD<br>Setting: MD<br>Monitoring: MD<br>Dental procedure: restorative dental treatment (*n*=60)	Nr. of reported effective sedations:MD<br><br>Sedation score,scales: MD<br><br>Behavior score and scales: MD<br><br>Adverse events/complications: None (*n*=60)<br><br>Management of adverse events/complications: NA<br><br>Procedure completion: MD<br>Reason for interruption: NA	MD<br>MD<br>MD
	RCT	45<br>2-6 yo (*n*=45)<br>22M, 23F<br>MD<br>None (*n*=45)	Midazolam (*n*=45)<br>IN (*n*=45)<br>0.3 mg/kg (*n*=45)<br>Onset: range 5-10 (*n*=45)<br>Duration: MD<br>Sleep: MD<br>Recovery time/score (mean, min): 31.69±3.37, range 26-40 (*n*=45)<br>Provider: MD<br>Setting: MD	Nr. of reported effective sedations:(*n*=38)<br><br>Sedation score and scales: 5- point sedation rating scale , N/d score<br><br>Behavior score and scales: 5- point behavior/response to treatment (‘Satisfactory” -rating score of 4/5 in the first 30 minutes; “Unsatisfactory”- score less than 4/5 in the first 30 min):	MD<br>MD<br>MD

			Monitoring: MD<br>Dental procedure: restorative dental treatment without pulp involvement (*n*=45)	"successful " 69% (*n*=31); "unsuccessful" 31% (*n*=14)<br><br>Adverse events/complications: none (*n*=45)<br>Management of adverse events/complications: NA<br><br>Procedure completion: yes (*n*=37); no (*n*=8)<br><br>Reason for interruption: uncooperative behavior (*n*=8)	
	RCT	16<br>Under 3 years of age (*n*=16)<br>MD<br>MD<br>MD	Midazolam (*n*=16)<br>PO (*n*=16)<br>1.0 mg/kg (*n*=16)<br>Onset: MD<br>Duration : MD<br>Sleep: MD<br>Recovery time/score: MD<br>Provider: MD<br>Setting: MD<br>Monitoring: MD<br>Dental procedure: restorative dental treatment (*n*=16)	Nr. of reported effective sedations: Success rate (%): MD<br><br>Scales: NA<br><br>Behavior score and scales: OSUBRS 14.0 ± 3.8<br>Adverse events/complications: None (*n*=16)<br>Management of adverse events/complications: NA<br><br>Procedure completion: yes (*n*=16)<br><br>Reason for interruption: NA	MD<br>MD<br>MD

	RCT	28<br>under 7 years of age (*n*=28)<br>MD<br>MD<br>None (*n*=28)	Midazolam (*n*=28)<br>PO (n =28)<br>1.0 mg/kg, max 20.0 mg (*n*=28)<br>Onset: MD<br>Duration MD<br>Sleep: MD<br>Recovery time: MD<br>Provider: anesthesiologist<br>Setting: university outpatient clinic (*n*=28)<br>Monitoring:<br>Dental procedure: restorative treatment (*n*=28)<br>Sedation type:	Nr. of reported effective sedations:<br>Success rate: MD<br><br>Scales: NA<br><br>Behavior score and scales: OSUBRS (*n*=28), intraoperative:<br>restless (*n*=6); quiet a few times (*n*=11); quiet most time (*n*=8); often quiet (*n*=3)<br><br>Adverse events/complications: yes (*n*=11):<br>vomiting (*n*=2); prolonged recovery (*n*=3); nausea/vomiting (*n*=6); irritability (*n*=5); agitation (*n*=2); drowsiness (*n*=1); hallucination (*n*=1)<br><br>Management of adverse events/complications: MD<br><br>Procedure completion: yes (*n*=24, of which *n*=21 with physical restraint); no (*n*=4)<br>Reason for interruption: MD	MD<br>MD<br>MD
Swaminatan 2025<br>J Dent Anesth Pain Med<br>[32]<br>14 RCT<br>Meta-analysis					
	RCT	15<br>MD<br>MD<br>MD<br>MD	Dexmedetomidine (*n*=15)<br>IN (*n*=15)<br>1μg/kg<br>Onset: MD<br>Duration MD<br>Sleep: MD<br>Recovery time: MD<br>Provider: MD	Nr. of reported effective sedations:MD<br><br>Sedation score, scales: Ramsay Sedation Scale (N/d scores) (*n*=15)<br><br>Behavior score, scales: Houpt Behavior scale (*n*=15)	MD<br>MD<br>MD

			Setting: MD<br>Monitoring: MD<br>N/d dental procedure (*n*=15)	Adverse events/complications: MD<br><br>Management of adverse events/complications: MD<br><br>Procedure completion: MD<br>Reason for interruption: MD	
			Midazolam (*n*=15)<br>IN (*n*=15)<br>0.3 mg/kg (*n*=15)<br>Onset: MD<br>Duration MD<br>Sleep: MD<br>Recovery time: MD<br>Provider: MD<br>Setting: MD<br>Monitoring: MD<br>N/d dental procedure (*n*=15)	Nr. of reported effective sedations:MD<br><br>Sedation score, scales: Ramsay Sedation Scale (N/d scores) (*n*=15)<br><br>Behavior score, scales: Houpt Behavior scale (*n*=15)<br><br>Adverse events/complications: MD<br><br>Management of adverse events/complications: MD<br><br>Procedure completion: MD<br>Reason for interruption: MD	
			N_2O (*n*=15)<br>Ihnalation (*n*=15)<br>N/d (N_2O/O_2 %) (*n*=15)<br>Onset: MD<br>Duration MD<br>Sleep: MD<br>Recovery time: MD<br>Provider: MD<br>Setting: MD<br>Monitoring: MD<br>N/d dental procedure (*n*=15)	Nr. of reported effective sedations:MD<br><br>Sedation score, scales: Ramsay Sedation Scale (N/d scores) (*n*=15)<br><br>Behavior score, scales: Houpt Behavior scale (*n*=15)<br><br>Adverse events/complications: MD<br><br>Management of adverse events/complications: MD<br><br>Procedure completion: MD<br>Reason for interruption: MD	

	RCT	42<br>5-7 yo (*n*=42)<br>N/D gender ratio<br>MD<br>MD	Dexmedetomidine (*n*=42)<br>IN (*n*=42)<br>1µg/kg (*n*=42)<br>Onset: MD<br>Duration MD<br>Sleep: MD<br>Recovery time: MD<br>Provider: MD<br>Setting: MD<br>Monitoring: MD<br>N/d dental procedure (*n*=42)	Nr. of reported effective sedations:MD<br><br>Sedation score, scales: MD<br><br>Behavior score, scales : Venham's rating scale for anxiety (N/d scores) (*n*=42);<br><br>Adverse events/complications: MD<br><br>Management of adverse events/complications: MD<br><br>Procedure completion: MD<br>Reason for interruption: MD	N/d acceptance scores (*n*=42)<br>N/d parental report (*n*=42)<br>MD
			Dexmedetomidine (*n*=42)<br>SL (*n*=42)<br>1µg/kg (*n*=42)<br>Onset: MD<br>Duration MD<br>Sleep: MD<br>Recovery time: MD<br>Provider: MD<br>Setting: MD<br>Monitoring: MD<br>N/d dental procedure (*n*=42)	Nr. of reported effective sedations:MD<br><br>Sedation score, scales: MD<br><br>Behavior score, scales: Venham's rating scale for anxiety (N/d scores) (*n*=42);<br><br>Adverse events/complications: MD<br><br>Management of adverse events/complications: MD<br><br>Procedure completion: MD<br>Reason for interruption: MD	
	RCT	92<br>4-6 yo (*n*=92)<br>MD<br>MD<br>MD	Dexmedetomidine (*n*=46)<br>IN (*n*=46)<br>1µg/kg (*n*=46)<br>Onset: MD<br>Duration MD<br>Sleep: MD<br>Recovery time: MD<br>Provider: MD<br>Setting: MD<br>Monitoring: MD	Nr. of reported effective sedations:MD<br><br>Sedation score, scales: Houpt Sedation Rating Scale (N/d scores) (*n*=46)<br><br>Behavior score, scales: CFSS-DS: "acceptable behavior" (47.7%) (*n*=22);<br><br>Adverse events/complications: MD	MD<br>MD<br>MD

			N/d dental procedure<br>(*n*=46)	Management of adverse events/complications: MD<br><br>Procedure completion: MD<br>Reason for interruption: MD	
			Midazolam (*n*=46)<br>IN (*n*=46)<br>1µg/kg (*n*=46)<br>Onset: MD<br>Duration MD<br>Sleep: MD<br>Recovery time: MD<br>Provider: MD<br>Setting: MD<br>Monitoring: MD<br>N/d dental procedure<br>(*n*=46)	Nr. of reported effective sedations:MD<br><br>Sedation score, scales: Houpt Sedation Rating Scale (N/d scores) (*n*=46)<br><br>Behavior score, scales: CFSS-DS: “acceptable behavior” (64%) (*n*=29);<br>Adverse events/complications: MD<br>Management of adverse events/complications: MD<br><br>Procedure completion: MD<br>Reason for interruption: MD	
	RCT	35<br>4-7 yo (*n*=35)<br>MD<br>MD<br>MD	Midazolam (*n*=35)<br>IN (*n*=35)<br>0.3 mg/kg (*n*=35)<br>Onset: MD<br>Duration MD<br>Sleep: MD<br>Recovery time: MD<br>Provider: MD<br>Setting: MD<br>Monitoring: MD<br>N/d dental procedure<br>(*n*=35)	Nr. of reported effective sedations:MD<br><br>Sedation score, scales: Ellis Sedation Scale (N/d scores) (*n*=35)<br><br>Behavior score, scales: Houpt Behavior Rating Scale (N/d scores) (*n*=35);<br>FLACC score (N/d scores) (*n*=35)<br><br>Adverse events/complications: MD<br>Management of adverse events/complications: MD<br><br>Procedure completion: MD<br>Reason for interruption: MD	MD<br>MD<br>MD
			N_2O (*n*=35)<br>Ihnalation (*n*=35)<br>30/70 (N_2O/O_2 %) (*n*=35)<br>Onset: MD<br>Duration MD	Nr. of reported effective sedations:MD<br><br>Sedation score, scales: Ellis Sedation Scale (N/d scores) (*n*=35)	

			Sleep: MD<br>Recovery time: MD<br>Provider: MD<br>Setting: MD<br>Monitoring: MD<br>N/d dental procedure (*n*=35)	Behavior score, scales: Houpt Behavior Rating Scale (N/d scores) (*n*=35);<br>FLACC score (N/d scores) (*n*=35)<br><br>Adverse events/complications: MD<br>Management of adverse events/complications: MD<br><br>Procedure completion: MD<br>Reason for interruption: MD	
	RCT	28<br>MD<br>MD<br>MD<br>MD	Midazolam (*n*=28)<br>PO (*n*=28)<br>1.0 mg/kg (*n*=28)<br>Onset: MD<br>Duration MD<br>Sleep: MD<br>Recovery time: MD<br>Provider: MD<br>Setting: MD<br>Monitoring: MD<br>N/d dental procedure (*n*=28)	Nr. of reported effective sedations:MD<br><br>Sedation score, scales: N/d<br><br>Behavior score, scales: Ohio State University BehaviPO Rating Scale (OSUBRS): “quite behavior” (32.1%), (*n*=9)<br><br>Adverse events/complications: MD<br>Management of adverse events/complications: MD<br><br>Procedure completion: MD<br>Reason for interruption: MD	MD<br>MD<br>MD
	RCT	20<br>3-7 yo (*n*=20)<br>MD<br>MD<br>MD	Midazolam (*n*=10)<br>IN (*n*=10)<br>0.2 mg/kg (*n*=10)<br>Onset: MD<br>Duration MD<br>Sleep: MD<br>Recovery time: MD<br>Provider: MD<br>Setting: MD<br>Monitoring: MD<br>N/d dental procedure (*n*=10)	Nr. of reported effective sedations:MD<br><br>Sedation score, scales: N/d<br><br>Behavior score, scales: Venham’s Anxiety Scale (N/d score) (*n*=10); salivary cortisol levels (N/d score) (*n*=10)<br>Adverse events/complications: MD<br>Management of adverse events/complications: MD	MD<br>MD<br>MD

				Procedure completion: MD<br>Reason for interruption: MD	
			Midazolam (*n*=10)<br>SL (*n*=10)<br>0.2 mg/kg (*n*=10)<br>Onset: MD<br>Duration MD<br>Sleep: MD<br>Recovery time: MD<br>Provider: MD<br>Setting: MD<br>Monitoring: MD<br>N/d dental procedure (*n*=10)	Nr. of reported effective sedations:MD<br><br>Sedation score, scales: N/d<br><br>Behavior score, scales: Venham's Anxiety Scale (N/d score) (*n*=10); salivary cortisol levels (N/d score) (*n*=10)<br>Adverse events/complications: MD<br>Management of adverse events/complications: MD<br><br>Procedure completion: MD<br>Reason for interruption: MD	
	RCT	20<br>3-7 yo (*n*=20)<br>MD<br>MD<br>MD	Midazolam (*n*=10)<br>IN (*n*=10)<br>0.2 mg/kg (*n*=10)<br>Onset: MD<br>Duration MD<br>Sleep: MD<br>Recovery time: MD<br>Provider: MD<br>Setting: MD<br>Monitoring: MD<br>N/d dental procedure (*n*=10)	Nr. of reported effective sedations:MD<br><br>Sedation score, scales: N/d<br><br>Behavior score, scales: Venham's Anxiety Scale (N/d score) (*n*=10); salivary cortisol levels (N/d score) (*n*=10)<br>Adverse events/complications: MD<br>Management of adverse events/complications: MD<br><br>Procedure completion: MD<br>Reason for interruption: MD	MD<br>MD<br>MD
			Midazolam (*n*=10)<br>SL (*n*=10)<br>0.2 mg/kg (*n*=10)<br>Onset: MD<br>Duration MD<br>Sleep: MD<br>Recovery time: MD	Nr. of reported effective sedations:MD<br><br>Sedation score, scales: N/d<br><br>Behavior score, scales: Venham's Anxiety Scale (N/d score) (*n*=10);	

			Provider: MD<br>Setting: MD<br>Monitoring: MD<br>N/d dental procedure (*n*=10)	salivary cortisol levels (N/d score) (*n*=10)<br>Adverse events/complications: MD<br>Management of adverse events/complications: MD<br><br>Procedure completion: MD<br>Reason for interruption: MD	
	RCT	40<br>3-7 yo (*n*=40)<br>MD<br>MD<br>MD	Midazolam (*n*=20)<br>IN (*n*=20)<br>0.2 mg/kg (*n*=20)<br>Mean onset: 9.40 ± 1.84 min (*n*=20)<br>Onset: MD<br>Duration MD<br>Sleep: MD<br>Recovery time: MD<br>Provider: MD<br>Setting: MD<br>Monitoring: MD<br>N/d dental procedure (*n*=20)	Nr. of reported effective sedations:MD<br><br>Sedation score, scales: MD<br><br>Behavior score, scales: Modified Houpt Behavior rating scale (N/d score) (*n*=20)<br><br>Adverse events/complications: MD<br>Management of adverse events/complications: MD<br><br>Procedure completion: MD<br>Reason for interruption: MD	MD<br>MD<br>MD
			Midazolam (*n*=20)<br>SL (*n*=20)<br>0.2 mg/kg (*n*=20)<br>Mean onset: 13.80 ± 2.04 min (*n*=20)<br>Onset: MD<br>Duration MD<br>Sleep: MD<br>Recovery time: MD<br>Provider: MD<br>Setting: MD<br>Monitoring: MD<br>N/d dental procedure (*n*=20)	Nr. of reported effective sedations:MD<br><br>Sedation score, scales: MD<br><br>Behavior score, scales: Modified Houpt Behavior rating scale (N/d score) (*n*=20)<br><br>Adverse events/complications: MD<br>Management of adverse events/complications: MD<br><br>Procedure completion: MD<br>Reason for interruption: MD	
	RCT	25<br>3-6 yo (*n*=25)<br>MD	Midazolam (*n*=25)<br>Buccal (*n*=25)<br>0.3 mg/kg	Nr. of reported effective sedations:MD	N/d acceptability (*n*=25)<br>MD<br>MD

		MD<br>MD	onset: up to 20 minutes (*n*=25)<br>Duration MD<br>Sleep: MD<br>Recovery time: MD<br>Provider: MD<br>Setting: MD<br>Monitoring: MD<br>N/d dental procedure (*n*=25)	Sedation score, scales: Houpt Behavior Rating Scale for sedation success and Behavior control (N/d scores) (*n*=25)<br><br>Behavior score, scales: Houpt Behavior Rating Scale for sedation success and Behavior control (N/d scores) (*n*=25)<br><br>Adverse events/complications: MD<br>Management of adverse events/complications: MD<br><br>Procedure completion: MD<br>Reason for interruption: MD	
			Midazolam (*n*=25)<br>IN via atomizer (*n*=25)<br>0.3 mg/kg<br>Onset: 10-15 minutes (*n*=25)<br>Duration MD<br>Sleep: MD<br>Recovery time: MD<br>Provider: MD<br>Setting: MD<br>Monitoring: MD<br>N/d dental procedure (*n*=25)	Nr. of reported effective sedations:MD<br><br>Sedation score, scales: Houpt Behavior Rating Scale for sedation success and Behavior control (N/d scores) (*n*=25)<br><br>Behavior score, scales: Houpt Behavior Rating Scale for sedation success and Behavior control (N/d scores) (*n*=25)<br><br>Adverse events/complications: MD<br>Management of adverse events/complications: MD<br><br>Procedure completion: MD<br>Reason for interruption: MD	
	RCT	30<br>2-8 yo (*n*=30)<br>MD<br>MD<br>MD	Midazolam (*n*=30)<br>IN (*n*=30)<br>0.25 mg/kg<br>Onset: MD<br>Duration MD<br>Sleep: MD<br>Recovery time: MD<br>Provider: MD<br>Setting: MD	Nr. of reported effective sedations:MD<br><br>Sedation score, scales: Houpt Behavior Rating Scale for sedation success and Behavior control (N/d scores) (*n*=30)<br><br>Behavior score, scales: Houpt Behavior Rating Scale for sedation success and Behavior control (N/d scores) (*n*=30)	Acceptance: “no complaints” (16.7%) (*n*=4), self-reported<br>MD<br>MD

			Monitoring: MD<br>N/d dental procedure<br>(*n*=30)	Adverse events/complications: MD<br>Management of adverse events/complications: MD<br><br>Procedure completion: MD<br>Reason for interruption: MD	
			Midazolam (*n*=30)<br>Buccal via spray (*n*=30)<br>0.25 mg/kg<br>Onset: MD<br>Duration MD<br>Sleep: MD<br>Recovery time: MD<br>Provider: MD<br>Setting: MD<br>Monitoring: MD<br>N/d dental procedure<br>(*n*=30)	Nr. of reported effective sedations:MD<br><br>Sedation score, scales: Houpt Behavior Rating Scale for sedation success and Behavior control (N/d scores) (*n*=30)<br><br>Behavior score, scales: Houpt Behavior Rating Scale for sedation success and Behavior control (N/d scores) (*n*=30)<br><br>Adverse events/complications: MD<br>Management of adverse events/complications: MD<br><br>Procedure completion: MD<br>Reason for interruption: MD	Acceptance: “no complaints” (83.3%), self-reported (*n*=21)<br>MD<br>MD
	RCT	45<br>2-6 yo<br>MD<br>MD<br>MD	Midazolam (*n*=15)<br>IN (*n*=15)<br>0.3 mg/kg (*n*=15)<br>Onset (mean): 6.80 min (*n*=15)<br>Onset: MD<br>Duration MD<br>Sleep: MD<br>Recovery time: MD<br>Provider: MD<br>Setting: MD<br>Monitoring: MD<br>N/d dental procedure<br>(*n*=15)	Nr. of reported effective sedations: (*n*=10)<br><br>Sedation score, scales: Sedation depth assessment scale (N/d scores) (*n*=15)<br><br>Behavior score, scales: BehaviPO response scale (N/d scores);<br><br>Adverse events/complications: MD<br>Management of adverse events/complications: MD<br><br>Procedure completion: MD<br>Reason for interruption: MD	MD<br>MD<br>MD

			Ketamine (*n*=15)<br>IN (*n*=15)<br>6 mg/kg (*n*=15)<br>onset (mean): 5.79 min (*n*=15)<br>Duration MD<br>Sleep: MD<br>Recovery time: MD<br>Provider: MD<br>Setting: MD<br>Monitoring: MD<br>N/d dental procedure (*n*=15)	Nr. of reported effective sedations: (*n*=14)<br><br>Sedation score, scales: Sedation depth assessment scale (N/d scores) (*n*=15)<br><br>Behavior score, scales: BehaviPO response scale (rating ease of treatment completion) (N/d scores) (*n*=15)<br><br>Adverse events/complications: MD<br>Management of adverse events/complications: MD<br><br>Procedure completion: MD<br>Reason for interruption: MD	
	RCT	31<br>3.6- 7 yo (*n*=31)<br>MD<br>MD<br>MD	Midazolam (*n*=31)<br>IN (*n*=31)<br>0.3 mg/kg (*n*=31)<br>Onset: MD<br>Duration MD<br>Sleep: MD<br>Recovery time: MD<br>Provider: MD<br>Setting: MD<br>Monitoring: MD<br>N/d dental procedure (*n*=31)	Nr. of reported effective sedations: MD<br><br>Sedation score, scales: MD<br><br>Behavior score, scales: Modified Houpt (*n*=31)<br>Adverse events/complications: MD<br>Management of adverse events/complications: MD<br><br>Procedure completion: MD<br>Reason for interruption: MD	MD acceptability<br>MD<br>"Effective" by providers, self-reported (*n*=31)
			Midazolam (*n*=31)<br>PO (*n*=31)<br>0.5 mg/kg (*n*=31)<br>Onset: MD<br>Duration MD<br>Sleep: MD<br>Recovery time: MD<br>Provider: MD<br>Setting: MD<br>Monitoring: MD	Nr. of reported effective sedations: MD<br><br>Sedation score, scales: MD<br><br>Behavior score, scales: Modified Houpt (*n*=31)<br>Adverse events/complications: MD<br>Management of adverse events/complications: MD<br><br>Procedure completion: MD	MD acceptability<br>MD<br>"Very Effective" by providers, self-reported (*n*=31)

			N/d dental procedure (*n*=31)	Reason for interruption: MD	
	RCT	40<br>2-5 yo (*n*=40)<br>MD<br>MD<br>MD	Midazolam (*n*=20)<br>IN (*n*=20)<br>0.2 mg/kg (*n*=20)<br>Onset: (mean): 10.8 min (*n*=20)<br>Duration MD<br>Sleep: MD<br>Recovery time: MD<br>Provider: MD<br>Setting: MD<br>Monitoring: MD<br>N/d dental procedure (*n*=20)	Nr. of reported effective sedations: MD<br><br>Sedation score, scales: MD<br><br>Behavior score, scales: Modified Houpt (*n*=20)<br>Adverse events/complications: MD<br>Management of adverse events/complications: MD<br><br>Procedure completion: MD<br>Reason for interruption: MD<br>MD	MD<br>MD<br>MD
			Midazolam (*n*=20)<br>IM (*n*=20)<br>0.2 mg/kg (*n*=20)<br>Onset: (mean) 15.7 min (*n*=20)<br>Duration MD<br>Sleep: MD<br>Recovery time: MD<br>Provider: MD<br>Setting: MD<br>Monitoring: MD<br>N/d dental procedure (*n*=20)	Nr. of reported effective sedations: MD<br><br>Sedation score, scales: MD<br><br>Behavior score, scales: Modified Houpt (*n*=20)<br>Adverse events/complications: MD<br>Management of adverse events/complications: MD<br><br>Procedure completion: MD<br>Reason for interruption: MD<br>MD	
		40<br>2-6 yo (*n*=40)<br>MD<br>MD<br>MD	Midazolam (*n*=20)<br>IN (*n*=20)<br>0.3 mg/kg (*n*=20)<br>Onset: (mean): 5.55 min (*n*=20)<br>Duration: MD<br>Recovery: 29.3 min (*n*=20)<br>N/d sleep (*n*=20)<br>Setting: MD<br>Provider: MD	Nr. of reported effective sedations: MD<br><br>Sedation score, scales: MD<br><br>Behavior score, scales: Houpt (*n*=20)<br>Adverse events/complications: MD<br>Management of adverse events/complications: MD<br><br>Procedure completion: MD	MD<br>MD<br>MD

			Monitoring: MD<br>N/d dental procedure (*n*=20)	Reason for interruption: MD	
			Midazolam (*n*=20)<br>PO (*n*=20)<br>0.7 mg/kg (*n*=20)<br>onset (mean): 15.5 min (*n*=20)<br>Duration: MD<br>Recovery: 38.1 min (*n*=20)<br>N/d sleep (*n*=20)<br>Setting: MD<br>Provider: MD<br>Monitoring: MD<br>N/d dental procedure (*n*=20)	Nr. of reported effective sedations: MD<br><br>Sedation score, scales: MD<br><br><br>Modified Houpt Behavior Rating Scale (N/d scores) (*n*=20)<br><br>Adverse events/complications: MD<br>Management of adverse events/complications: MD<br><br>Procedure completion: MD<br>Reason for interruption: MD	
	RCT	120<br>4-6 yo (*n*=120)<br>MD<br>MD<br>MD	Midazolam (*n*=60)<br>IN (*n*=60)<br>0.2 mg/kg (*n*=60)<br>Onset: 5-10 min (*n*=60)<br>Duration: MD<br>Recovery: MD<br>N/d sleep MD<br>Setting: MD<br>Provider: MD<br>Monitoring: MD<br>N/d dental procedure (*n*=60)	Nr. of reported effective sedations: MD<br><br>Sedation score, scales: MD<br><br><br>Behavior score,scale: Houpt Behavior Rating Scale (N/d scores) (*n*=60)<br><br>Adverse events/complications: MD<br>Management of adverse events/complications: MD<br><br>Procedure completion: MD<br>Reason for interruption: MD	MD<br>MD<br>MD
			Midazolam (*n*=60)<br>PO (*n*=60)<br>0.5 mg/kg (*n*=60)<br>Onset: (mean): 15-20 min (*n*=60)<br>Duration: MD<br>Recovery: MD	Nr. of reported effective sedations: MD<br><br>Sedation score, scales: MD<br><br><br>Behavior score,scale: Houpt Behavior Rating Scale (N/d scores) (*n*=60)	

			N/d sleep MD<br>Setting: MD<br>Provider: MD<br>Monitoring: MD<br>N/d dental procedure (*n*=60)	Adverse events/complications: MD<br>Management of adverse events/complications: MD<br><br>Procedure completion: MD<br>Reason for interruption: MD	
Zupin 2024 Acta pediatrica [33]<br>1 RCT<br>No Meta-analysis					
	RCT	13<br>8.68 yo, range 5.8-14.7 yo<br>MD<br>Autism (*n*=13)	Diazepam (*n*=13)<br>PO (*n*=13)<br>0.3 mg/kg (*n*=13)<br>Onset: (mean) MD<br>Duration MD<br>Sleep: MD<br>Recovery time: MD<br>Provider: MD<br>Setting: MD<br>Monitoring: MD<br>N/d dental procedure (*n*=13)	Nr. of reported effective sedations:(*n*=13)<br>Sedation score, scales: MD<br><br>Modified Houpt Behavior Rating Scale (N/d scores) (*n*=20)<br><br>Adverse events/complications: None (*n*=13)<br>Management of adverse events/complications: MD<br><br>Procedure completion: MD<br>Reason for interruption: MD	MD<br>MD<br>MD
			Midazolam (*n*=13)<br>PO (*n*=13)<br>0.5 mg/kg (*n*=13)<br>MD<br>Onset: (mean) MD<br>Duration MD<br>Sleep: MD<br>Recovery time: MD<br>Provider: MD	Nr. of reported effective sedations:(*n*=13)<br>Sedation score, scales: MD<br><br>Modified Houpt Behavior Rating Scale (N/d scores) (*n*=20)	

			Setting: MD<br>Monitoring: MD<br>N/d dental procedure (*n*=13)	Adverse events/complications: None (*n*=13)<br>Management of adverse events/complications: MD<br><br>Procedure completion: MD<br>Reason for interruption: MD	

Abbreviations: Per os, "PO"; SL, "SL"; Intravenous, "IV"; Intranasal, "IN"; Intramuscular, "IM"; Per Rectum, "PR"; Missing data, "MD"; Not applicable, "NA"; Not defined, "N/d"; Years old, "yo"; Number, "n"; Milligram, "mg"; Minutes, "min"; Males/Females, "M/F"; Milligram per kilogram, "mg/kg"; Electrocardiogram, "ECG"; Modified Observer Assessment of Alertness/Sedation scale, "MOAAS"; Face, Legs, Activity, Cry, Consolability scale, "FLACC"; Children's Fear Survey Schedule – Dental Subscale, "CFSS-DS"; Ohio State University Behavior Rating Scale, "OSUBRS"; milliliters, "ml"; Micron, "μm"; American Academy of Pediatric Dentistry, "AAPD"; Inhalation route, "INH"; Nitrogen, "N_2"; Oxygen, "O_2"; percentage, "%"; Visual Analog Scale, "VAS".
